# A new type of C_2_H_2_ binding site in a *cis*-bridging hexafluorosilicate ultramicroporous material that offers trace C_2_H_2_ capture†

**DOI:** 10.1039/d5sc00697j

**Published:** 2025-04-23

**Authors:** Bai-Qiao Song, Mei-Yan Gao, Lisa Mercene van Wyk, Cheng-Hua Deng, Alan C. Eaby, Shi-Qiang Wang, Shaza Darwish, Dan Li, Shao-Jie Qin, Yun-Lei Peng, Qing-Yuan Yang, Leonard J. Barbour, Michael J. Zaworotko

**Affiliations:** a College of Materials and Chemistry & Chemical Engineering, Chengdu University of Technology Chengdu 610059 China bqsong@cdut.edu.cn; b Department of Chemical Sciences and Bernal Institute, University of Limerick Limerick V94 T9PX Republic of Ireland xtal@ul.ie; c Department of Chemistry and Polymer Science, University of Stellenbosch Matieland 7602 South Africa; d Department of Applied Chemistry, College of Science, China University of Petroleum-Beijing Beijing 102249 China ylpeng@cup.edu.cn; e School of Chemical Engineering and Technology, Xi'an Jiaotong University Xi'an 710049 China

## Abstract

Hybrid ultramicroporous materials (HUMs) comprising hexafluorosilicate (SiF_6_^2−^, SIFSIX) and their variants are promising physisorbents for trace acetylene (C_2_H_2_) capture and separation, where the inorganic anions serve as *trans*-bridging pillars. Herein, for the first time, we report a strategy of fluorine binding engineering in these HUMs *via* switching the coordination mode of SIFSIX from traditional *trans* to rarely explored *cis*. The first example of a rigid HUM involving *cis*-bridging SIFSIX, SIFSIX-bidmb-Cu (bidmb = 1,4-bis(1-imidazolyl)-2,5-dimethylbenzene), is reported. The resulting self-interpenetrated network is found to be water stable and exhibits strong binding to C_2_H_2_ but weak binding to C_2_H_4_ and CO_2_, affording a high *Q*_st_ of 55.7 kJ mol^−1^ for C_2_H_2_, a high C_2_H_2_ uptake of 1.86 mmol g^−1^ at 0.01 bar and high Δ*Q*_st_ values. Breakthrough experiments comprehensively demonstrate that SIFSIX-bidmb-Cu can efficiently capture and recover C_2_H_2_ from 50/50 or 1/99 C_2_H_2_/CO_2_ and C_2_H_2_/C_2_H_4_ binary mixtures. *In situ* single crystal X-ray diffraction (SCXRD) combined with dispersion-corrected density functional theory (DFT-D) calculations reveals that the C_2_H_2_ binding site involves two *cis*-SiF_6_^2−^ anions in close proximity (F⋯F distance of 7.16 Å), creating a new type of molecular trap that affords six uncoordinated fluoro moieties to chelate each C_2_H_2_*via* sixfold C–H⋯F hydrogen bonds. This work therefore provides a new strategy for binding site engineering with selective C_2_H_2_ affinity to enable trace C_2_H_2_ capture.

## Abstract 2

Highly selective acetylene binding over CO_2_ and ethylene is observed in the first example of a rigid sorbent that is composed of *cis*-bridging hexafluorosilicate anions.

## Graphical abstract

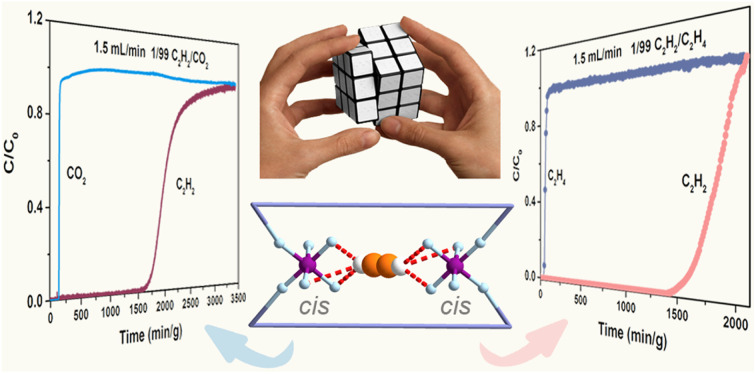

## Introduction

Acetylene (C_2_H_2_) is a high volume feedstock used for the production of various commodity chemicals.^1^ The production of C_2_H_2_ involves partial combustion of methane or thermal cracking of hydrocarbons, inevitably generating impurities, for example, carbon dioxide (CO_2_).^2^ As a result, purification of C_2_H_2_ is a needed prerequisite for its downstream use.^3^ Capture of trace C_2_H_2_ impurities from gas mixtures is also of importance: recovery of trace C_2_H_2_ is desirable in a circular economy and for safety, given that C_2_H_2_ is a flammable, explosive and toxic chemical;^4^ removal of trace C_2_H_2_ is a necessary procedure to produce polymer-grade ethylene (C_2_H_4_).^5^ Traditional purification technologies, such as organic solvent extraction (*e.g.*, *N*,*N*-dimethylformamide or acetone) or catalytic partial hydrogenation using noble metal catalysts,^6^ have high energy footprints and costs for waste solvent disposal. New approaches for trace C_2_H_2_ capture and separation are therefore desirable.^7^

Physisorption using porous materials is generally recognized as an efficient and energy-saving approach to C_2_H_2_ capture and separation.^4,8^ In this context, metal–organic materials such as metal–organic frameworks (MOFs)^9^ or coordination polymers (CPs)^10^ offer potential utility, thanks to their high surface area and modularity that allow for fine-tuning of the pore size, shape and chemistry.^11–22^ In this context, hybrid ultramicroporous materials (HUMs) composed of inorganic (*e.g.* SiF_6_^2−^ – SIFSIX) and organic linker ligands have been reported to show benchmark trace C_2_H_2_ capture performance, thanks to their sub-nanometer pore size (<0.7 nm). In these HUMs, the C_2_H_2_ molecules are bound to the pore surface *via* strong hydrogen bonds because of the relatively protic nature of the CH moieties of acetylene. For example, the strong C–H⋯F hydrogen bonds between C_2_H_2_ and the fluoro atom(s) of SiF_6_^2−^ (SIFSIX) anions^23–25^ exemplify how molecular recognition can drive selectivity towards C_2_H_2_.^26–28^

**Fig. 1:**
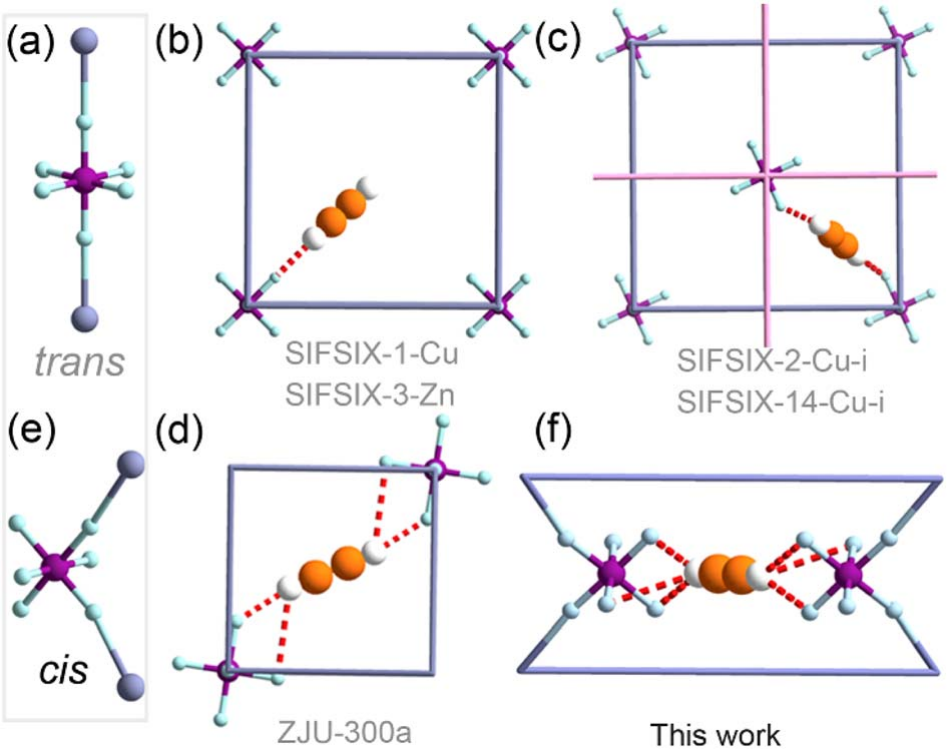
SIFSIX anions can bridge metals in either *trans*- (a) or *cis*- (e) mode. C_2_H_2_ binding sites in *trans*-bridging materials can involve (b) a single C–H⋯F interaction, (c) dual C–H⋯F interactions or (d) quadruple C–H⋯F interactions. Herein, we report (f) a new type of binding site with sixfold C–H⋯F interactions involving three free fluoro atoms from each of the two *cis*-SIFSIX anions.

In such HUM materials, SIFSIX anions invariably adopt a *trans*-bridging coordination mode (Fig. 1a, abbreviated hereafter as *trans*-SIFSIX).^29–32^ C_2_H_2_ binding in C_2_H_2_ selective *trans*-SIFSIX HUMs can be classified into one of the three modes (Fig. 1b–d):^33^ a single C–H⋯F interaction (*e.g.*, SIFSIX-1-Cu and SIFSIX-3);^23^ dual C–H⋯F interactions from two SIFSIX anions (*e.g.* SIFSIX-2-Cu-i and SIFSIX-14-Cu-i);^24^ quadruple C–H⋯F interactions from two SIFSIX anions involving bifurcated hydrogen bonds (*e.g.* ZJU-300a).^33^ The *trans*-bridging coordination geometry of the SIFSIX anion in fact limits the free (uncoordinated) fluoro sites accessible for each C_2_H_2_ molecule (up to two per anion, as shown in Fig. 1d).

Recently, the first *cis*-bridging coordination mode of SIFSIX anions was observed in a flexible HUM (Fig. 1e).^34–36^ This *cis*-bridging exposes four identically oriented fluoro moieties to the pore walls^28^ and promotes the possibility of formation of sixfold C–H⋯F interactions stemming from trifurcated hydrogen bonds (Fig. 1e and f). Herein, we report the first rigid HUM composed of *cis*-SIFSIX anions, [CuSiF_6_(bidmb)_2_]_*n*_ (SIFSIX-bidmb-Cu, where bidmb = 1,4-bis(1-imidazol-yl)-2,5-dimethyl benzene), and its sorption performance for C_2_H_2_ and related sorbates. As detailed herein, SIFSIX-bidmb-Cu is water stable with a binding site involving six fluoro moieties that offer outstanding separation performance for 50/50 or 1/99 mixtures of C_2_H_2_/CO_2_ and C_2_H_2_/C_2_H_4_.

## Results and discussion

### Synthesis and crystal structures

Slow diffusion of a methanol solution of bidmb into a water solution of CuSiF_6_·6H_2_O after one month generated SIFSIX-bidmb-Cu as purple block crystals suitable for single-crystal X-ray diffraction (SCXRD). Microcrystalline powders can be produced by a direct mixing method (Fig. S1†). The purity of bulk samples was confirmed by powder X-ray diffraction (PXRD) (Fig. S2†).

**Fig. 2:**
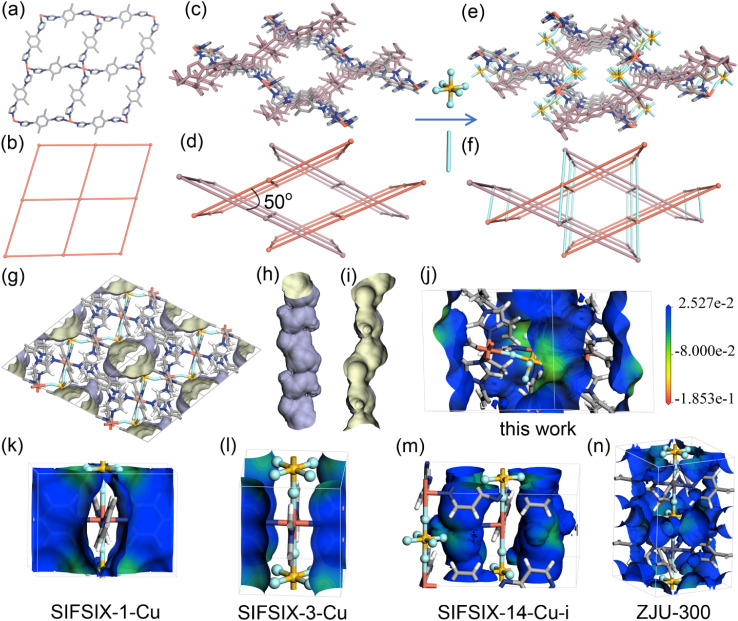
(a–f) Structural and topological representation of the Cu(bidmb)_2_**sql** layer (a and b), inclined interpenetrated **sql** nets (c and d) and the 3D self-catenated network formed by *cis*-SiF_6_^2−^ anions pillaring interpenetrated **sql** nets (e and f). (g) The 1D channels along the *b*-axis, (h) the exterior, and (i) interior pore wall of a single 1D channel, represented by the Connolly surface with a probe radius of 1.7 Å. (j–n) Electrostatic potential (ESP) of SIFSIX-bidmb-Cu (j), SIFSIX-1-Cu (k), SIFSIX-3-Cu (l), SIFSIX-14-Cu-i (m) and ZJU-300 (n) mapped onto the Connolly surface. The gradation on the scale bar is in Ha* electron (−1).

SCXRD analysis revealed that SIFSIX-bidmb-Cu crystallized in the triclinic space group *P*1̄ (Table S1†). All Cu^2+^ cations adopt the same octahedral coordination geometry with four imidazolyl nitrogen atoms from four bidmb ligands at the equatorial positions and two fluorine atoms from SIFSIX at the axial positions (Fig. S3†). Cu^2+^ and bidmb generate the expected square lattice (**sql**) topology networks of formula Cu(bidmb)_2_ (Fig. 2a and b and S4†). These **sql** layers exhibit inclined interpenetration subtended by an angle of *ca*. 50° (Fig. 2c and d). SIFSIX anions crosslink the resulting networks through a *cis*-bridging mode to form a 3D self-catenated network with the simplified topological point symbol of 4^8^.5^2^.6^5^ (Fig. 2e and f), a topology that was first observed in SIFSIX materials (Fig. S5†). In SIFSIX-bidmb-Cu, the *cis*-SIFSIX anions and Cu centers generate an undulating CuSIFSIX chain (Fig. S6a†). Hydrogen bonds between fluorine atoms and hydrogen atoms of bidmb (*d*_F⋯H_ = 2.3–3.4 Å) are present (Fig. S7 and S8†).

The structure contains one dimensional (1D) channels parallel to the crystallographic *b*-axis with a void space of 23.1% as determined by PLATON (Fig. 2g–i)^37^ occupied by disordered MeOH and water molecules (Fig. S9†). The diameter of the 1D channel is *ca*. 4.6 Å (Fig. S10†) and is inclined relative to the CuSiF_6_ chain at an angle of 62° (Fig. S6b and c†). The connectivity and pore structure are therefore distinct from **pcu** topology SIFSIX materials in which 1D channels align parallel to MSIFSIX chains. The inclined structure herein results in SIFSIX anions exposed to the channel with F⋯F distances of 7.16 Å (Fig. S11†). The electrostatic potential (ESP) of SIFSIX-bidmb-Cu and other benchmark SIFSIX materials was calculated to investigate the difference in pore chemistry arising from the coordination mode of SIFSIX anions (*trans vs. cis*). The ESP was mapped onto the Connolly surface with a probe radius of 1.7 Å, and the same color gradation scale was used for comparison (Fig. 2j–n). Thanks to the strong electronegativity of fluorine atoms, the most negative electrostatic potential (ESP) is distributed in the region close to the SIFSIX anions in all SIFSIX materials. However, in SIFSIX-bidmb-Cu, the ESP is more negative than that of other SIFSIX materials.

In the first flexible *cis*-SIFSIX HUM, the **sql** layers are also composed of bis-imidazolyl ligands, but Cu centers are parallel to each other and pillared by *cis*-SIFSIX anions to form a **pcu** topology network.^34–36^ In SIFSIX-bidmb-Cu, **sql** layers are inclined to interpenetrate, reducing the pore size and changing the pore chemistry. HUMs formed by angular inorganic anions that pillar interpenetrated **sql** nets are unusual, but as exemplified by **mmo** nets containing tetrahedral anions (XO_4_^2−^, where X = W and Mo)^38^ that bridge three sets of mutually perpendicular **sql** nets (Fig. S5†), exceptional sorption performance is possible.

### Characterization of SIFSIX-bidmb-Cu

TGA analysis revealed that SIFSIX-bidmb-Cu is thermally stable up to 230 °C with guest molecules released by *ca.* 120 °C (Fig. S12†). Variable-temperature PXRD (VT-PXRD) data are consistent with SIFSIX-bidmb-Cu being a rigid sorbent (Fig. S13†). SCXRD analysis of the fully activated form, SIFSIX-bidmb-Cu′, also suggests rigidity (Table S1†).

**Fig. 3:**
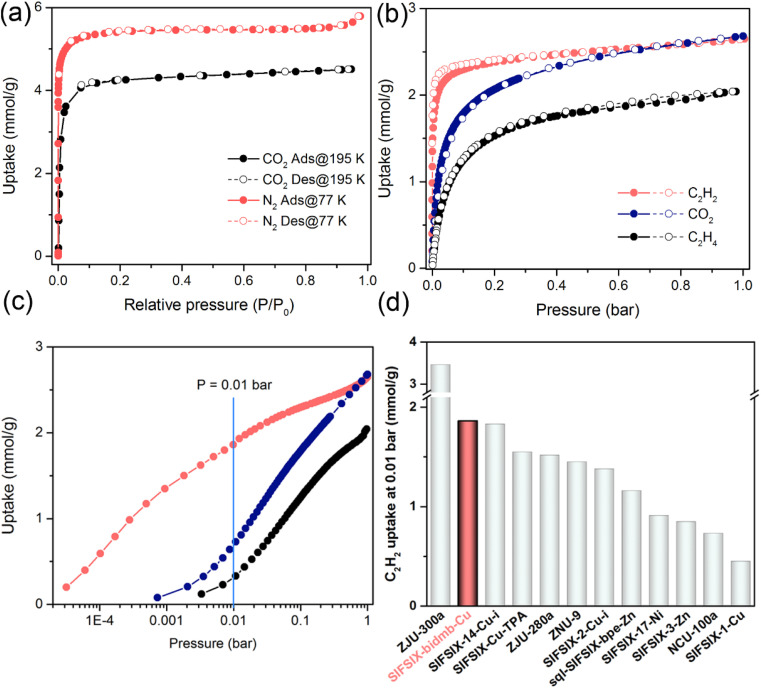
(a) CO_2_ (195 K) and N_2_(77 K) sorption isotherms. (b) and (c) C_2_H_2_, C_2_H_4_ and CO_2_ sorption isotherms collected at 298 K. (d) Comparison of C_2_H_2_ uptake at 0.01 bar for SIFSIX-bidmb-Cu and other SIFSIX materials.

The permanent porosity of SIFSIX-bidmb-Cu′ was tested by N_2_ (77 K) and CO_2_ (195 K) sorption experiments. Both gases exhibited type-I sorption isotherms with saturated uptake values of 5.79 and 4.51 mmol g^−1^, respectively (Fig. 3a). Based on the 77 K N_2_ adsorption isotherm, the Langmuir and Brunauer–Emmett–Teller surface areas were calculated to be 543.9 and 480 m^2^ g^−1^, respectively (Fig. S14 and S15†). The calculated pore volume, 0.16 cm^3^ g^−1^, is close to the theoretical value (0.18 cm^3^ g^−1^) on the basis of SCXRD. To our knowledge, SIFSIX-bidmb-Cu is the first rigid HUM (and only the second SIFSIX material) comprising *cis*-SIFSIX anions.^34–36^

### Single-component adsorption isotherms

The single-component sorption isotherms of SIFSIX-bidmb-Cu′ for C_2_H_2_/CO_2_/C_2_H_4_ were collected at 298 and 273 K. As shown in Fig. 3b, each gas exhibits a type-I sorption isotherm at 298 K. The saturated uptake values of C_2_H_2_, CO_2_ and C_2_H_4_ at 1 bar were observed to be 2.65, 2.68, and 2.04 mmol g^−1^, respectively. Notably, SIFSIX-bidmb-Cu showed much steeper C_2_H_2_ uptake than CO_2_ and C_2_H_4_ at lower pressures (Fig. 3c). Substantially higher C_2_H_2_ uptake was exhibited at 0.01 bar (1.86 mmol g^−1^) than for CO_2_ (0.64 mmol g^−1^) and C_2_H_4_ (0.33 mmol g^−1^). The uptake of C_2_H_2_ at 0.01 bar, an indicator of the trace C_2_H_2_ capture ability of a sorbent, is higher than most SIFSIX materials (Fig. 3d and S16†), such as SIFSIX-1-Cu (0.45 mmol g^−1^),^23^ SIFSIX-3-Zn (0.85 mmol g^−1^),^23^ and SIFSIX-2-Cu-i (1.38 mmol g^−1^),^23^ comparable to the benchmark SIFSIX-14-Cu-i (1.83 mmol g^−1^),^24^ but is lower than ZJU-300a (3.23 mmol g^−1^).^33^ The PXRD patterns of SIFSIX-bidmb-Cu′ collected at 273 and 298 K at 1 bar C_2_H_2_ are consistent with that calculated for SIFSIX-bidmb-Cu (Fig. S17†), further suggesting a rigid structure. Consecutive C_2_H_2_ adsorption–desorption experiments conducted at 298 K indicated that adsorbed C_2_H_2_ was not fully removed under vacuum at room temperature (about 50% uptake remained), requiring heating to 120 °C (Fig. S18†). This provides evidence of the strong binding affinity for C_2_H_2_ and also suggests a method to capture and recover C_2_H_2_*via* temperature control. Interestingly, the C_2_H_2_-saturated sample was regenerated under vacuum at room temperature after exposure to air for one year (Fig. S19–S21†). In contrast, multiple cycles of sorption experiments on CO_2_ and C_2_H_4_ displayed negligible changes even if regeneration was implemented under high vacuum at room temperature (Fig. S22 and S23†), implying weaker interactions with SIFSIX-bidmb-Cu′. The time-dependent adsorption kinetics profiles of SIFSIX-bidmb-Cu′ were also investigated at 298 K (Fig. S24†). SIFSIX-bidmb-Cu′ exhibited a notably faster initial rate for C_2_H_2_ adsorption than CO_2_, reaching 41.7% and 11.3% of 1 bar uptake in 40 seconds for C_2_H_2_ and CO_2_, respectively. C_2_H_2_ adsorption reached equilibrium within 25 min, but CO_2_ adsorption only reached *ca*. 40% of loading by this time.

The coverage-dependent isosteric heat of adsorption (*Q*_st_) of SIFSIX-bidmb-Cu′ for each gas derived from sorption isotherms indicated *Q*_st_ values at near-zero coverage as follows: C_2_H_2_ (55.7 kJ mol^−1^) > C_2_H_4_ (38.1 kJ mol^−1^) > CO_2_ (20.1 kJ mol^−1^) (Fig. S25–S28†). Notably, the *Q*_st_ value of C_2_H_2_ at a low loading (55.7 kJ mol^−1^) surpasses those of most prominent SIFSIX materials (Fig. S29†), including ZNU-9 (33.1 kJ mol^−1^),^39^ SIFSIX-21-Ni (37.9 kJ mol^−1^),^29^ SIFSIX-Cu-TPA (39 kJ mol^−1^),^40^ SIFSIX-14-Cu-i (40.0 kJ mol^−1^),^24^ SIFSIX-2-Cu-i (41.8 kJ mol^−1^)^23^ and SIFSIX-17-Ni (44.2 kJ mol^−1^),^41^ and is comparable to those of UTSA-300a (57.6 kJ mol^−1^),^30^ NCU-100a (60.5 kJ mol^−1^)^42^ and ZJU-300a (61.1 kJ mol^−1^).^33^ Conversely, the *Q*_st_(CO_2_) value at a low loading (20.1 kJ mol^−1^) is low, being marginally higher than that of SIFSIX-21-Ni (19.8 kJ mol^−1^).^29^ Consequently, the difference in *Q*_st_ values between C_2_H_2_ and CO_2_ (Δ*Q*_st,C_2_H_2_–CO_2__), 35.6 kJ mol^−1^, is to our knowledge the highest reported value for SIFSIX materials (SIFSIX-2-Cu-i = 9.9 kJ mol^−1^,^43^ SIFSIX-22-Zn = 11.5 kJ mol^−1^,^44^ SIFSIX-Cu-TPA = 13.4 kJ mol^−1^,^40^ and SIFSIX-21-Ni = 18.1 kJ mol^−1^) (Fig. S30 and S31†).^29^ Furthermore, this Δ*Q*_st_ value is higher than the values of other top-performing sorbents like SOFOUR-1-Zn (24 kJ mol^−1^),^44^ SOFOUR-TEPE-Zn (26.3 kJ mol^−1^),^45^ Cu(bpy)NP (26.2 kJ mol^−1^)^46^ and SNNU-98-Mn (31 kJ mol^−1^),^47^ comparable to that of Ni(4-DPDS)_2_CrO_4_ (38.4 kJ mol^−1^),^48^ but inferior to those of sorbents with open metal centres such as ATC-Cu (43.6 kJ mol^−1^)^49^ and Cu^I^@UiO-66-(COOH)_2_ (45.6 kJ mol^−1^).^50^ The difference between *Q*_st_(C_2_H_2_) and *Q*_st_(C_2_H_4_), Δ*Q*_st,C_2_H_2_–C_2_H_4__, of 17.6 kJ mol^−1^ is also comparable to that of the benchmark SIFSIX material ZJU-300a (21.1 kJ mol^−1^)^33^ and superior to those of SIFSIX-3-Ni (10.2 kJ mol^−1^),^51^ SIFSIX-2-Cu-i (11.2 kJ mol^−1^)^43^ and SIFSIX-14-Cu-i (13 kJ mol^−1^) (Fig. S32†).^24^ These *Q*_st_ and Δ*Q*_st_ values are indicative of the relative binding strength of SIFSIX-bidmb-Cu′ for these sorbates.

### Separation properties

To explore the separation properties of SIFSIX-bidmb-Cu′, selectivities for C_2_H_2_/CO_2_ and C_2_H_2_/C_2_H_4_ gas mixtures in different compositions at 100 kPa and 298 K were calculated from the ideal adsorbed solution theory (IAST) (Fig. S33–S42†). For both C_2_H_2_/CO_2_ and C_2_H_2_/C_2_H_4_, the selectivity values indicate potential for trace removal of C_2_H_2_. For 50/50 (v/v) C_2_H_2_/CO_2_ and 1/99 C_2_H_2_/C_2_H_4_, the two gas ratios most commonly investigated, the selectivity values were determined to be 20.3 and 140.2, respectively (Fig. S36†). The equimolar C_2_H_2_/CO_2_ selectivity of SIFSIX-bidmb-Cu′ is higher than most SIFSIX materials (Fig. S43†), *e.g.* SIFSIX-Cu-TPA (5.3),^40^ SIFSIX-21-Ni (10),^29^ ZNU-9 (10.3),^39^ SIFSIX-17-Ni (11.7)^41^ and ZJU-280a (18.1),^52^ and is only lower than those sorbents that exhibit molecular sieving, including UTSA-300a (743)^30^ and NCU-100a (1786.6).^42^ The 1/99 C_2_H_2_/C_2_H_4_ selectivity of SIFSIX-bidmb-Cu′ is also superior to that of most SIFSIX materials (Fig. S44†), including SIFSIX-3-Ni (5.03),^51^ SIFSIX-2-Cu (6),^23^ SIFSIX-1-Cu (10.63),^23^ ZNU-9 (11.64),^39^ SIFSIX-2-Cu-i (44.5),^43^ ZJU-280a (44.5),^52^ and sql-SIFSIX-bpe-Zn (53.1),^53^ but inferior to that of the molecular sieve SIFSIX-14-Cu-i (6320).^24^ Notably, SIFSIX-bidmb-Cu′ ranks second among sorbents that simultaneously exhibit 50/50 C_2_H_2_/CO_2_ and 1/99 C_2_H_2_/C_2_H_4_ selectivities, only being inferior to SIFSIX-TEPE-Cu (Fig. S45†).^54^

Since 2016, when SIFSIX HUMs were found to offer superior acetylene sorption/separation, different strategies have been tried to improve the sorption/separation performance, including pore size control *via* interpenetration (*e.g.* SIFSIX-2-Cu *vs.* SIFSIX-2-Cu-i),^23,24^ organic linker functionalization^41^ and anion replacement to fine-tune pore chemistry (*e.g.* SiF_6_^2−^ to TiF_6_^2−^).^51^ In contrast, C_2_H_2_ capture performance improvement *via trans*-to-*cis* bridging coordination mode change of SIFSIX anions has not been explored. This research work fills the gap and shows that *cis*-SIFSIX anions can also be favorable for the construction of C_2_H_2_-selective sorbents with *cis*-SIFSIX anions, offering more fluoro binding sites than *trans* analogs. The selectivity for 1/99 C_2_H_2_/C_2_H_4_ has been improved from 44.5 (SIFSIX-2-Cu-i) to 140.2 (SIFSIX-bidmb-Cu), while the selectivity for 50/50 C_2_H_2_/CO_2_ has been increased to 20.3 (SIFSIX-bidmb-Cu) from 6.5 (TIFSIX-2-Cu-i). The *cis*-bridging mode of SIFSIX anions resembles that of the tetrahedral anions XO_4_^2−^ (X = W, Mo, S *etc*.*.*), which are increasingly used in HUMs.^38,44,45^

### Dynamic breakthrough experiments

**Fig. 4:**
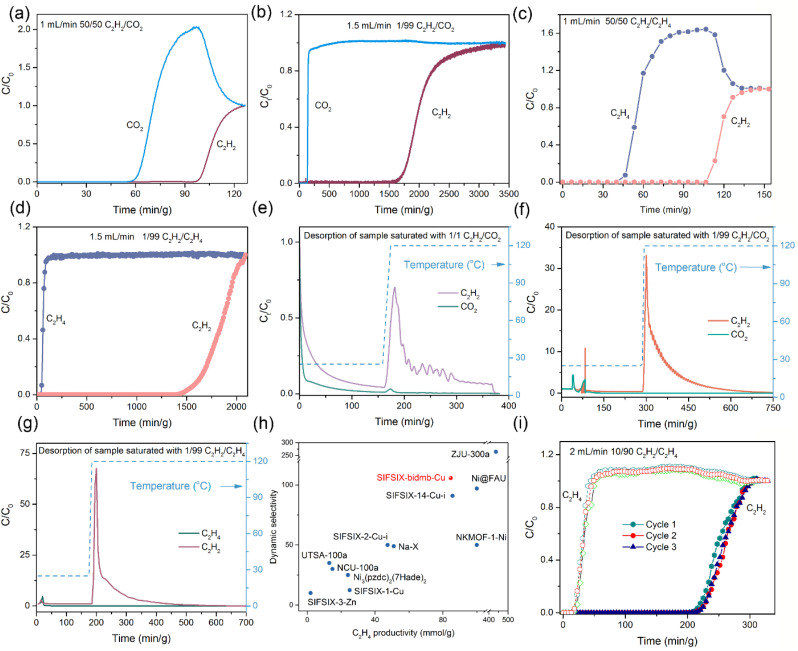
(a–d) Experimental breakthrough curves of equimolar C_2_H_2_/CO_2_ (a), 1/99 C_2_H_2_/CO_2_ (b), equimolar C_2_H_2_/C_2_H_4_ (c) and 1/99 C_2_H_2_/C_2_H_4_ (d) mixtures on SIFSIX-bidmb-Cu′. (e) and (f) Desorption curves of samples saturated with 1/1 (e) and 1/99 C_2_H_2_/CO_2_ (f) and 1/99 C_2_H_2_/C_2_H_4_ (g) mixtures. (h) Comparison of C_2_H_4_ productivity *versus* dynamic selectivity (1/99 C_2_H_2_/C_2_H_4_) for SIFSIX-bidmb-Cu′ and other benchmark sorbents. (i) Three consecutive cycles of breakthrough curves for a 10/90 C_2_H_2_/C_2_H_4_ binary mixture on SIFSIX-bidmb-Cu′.

The experimental separation performance of SIFSIX-bidmb-Cu′ for C_2_H_2_/CO_2_ and C_2_H_2_/C_2_H_4_ mixtures was tested by transient column breakthrough experiments performed at 298 K and 100 kPa (Fig. 4a–d). SIFSIX-bidmb-Cu′ showed excellent C_2_H_2_ separation performance for both 50/50 and 1/99 binary mixtures. For C_2_H_2_/CO_2_ mixtures, CO_2_ breakthrough occurred first with retention times of 59.7 and 128.1 min g^−1^ for the 50/50 and 1/99 mixtures, respectively (Fig. 4a and b). In contrast, the corresponding C_2_H_2_ breakthrough occurred after retention times of 98.6 and 1591.2 min g^−1^, respectively. The breakthrough times for C_2_H_2_ and CO_2_ were 38.9 and 1463.1 min g^−1^ for 50/50 and 1/99 gas mixtures, respectively. The calculated C_2_H_2_ uptake values were 2.48 and 1.38 mmol g^−1^ for 50/50 and 1/99 mixtures, respectively. The former is comparable to that of the benchmark materials, including JCM-1 (2.2 mmol g^−1^),^55^ NKMOF-1-Ni (2.48 mmol g^−1^),^56^ Cu^I^@UiO-66-(COOH)_2_ (2.89 mmol g^−1^)^50^ and Ni(4-DPDS)_2_CrO_4_ (2.96 mmol g^−1^).^48^ Captured C_2_H_2_ can be recovered with high purity (>99% purity) through heating the saturated sample at 120 °C after removal of co-adsorbed CO_2_*via* a period of helium flush at room temperature (the longer the flushing time, the purer the concentration of C_2_H_2_) (Fig. 4e and f). The temperature-controlled recovery of C_2_H_2_ is in good agreement with the sorption experiments where heating at 120 °C was needed to fully remove the strongly adsorbed C_2_H_2_ molecules. To our knowledge, SIFSIX-bidmb-Cu is just the second sorbent that yields high purity C_2_H_2_ (>99% purity) from a 1/99 C_2_H_2_/CO_2_ stream on desorption,^44^ which is relevant for recycling of C_2_H_2_, safety and environmental protection. The equimolar C_2_H_2_/CO_2_ separation factor for SIFSIX-bidmb-Cu, a criterion for evaluating the separation potential of sorbents, was calculated to be 7.8, higher than those of SIFSIX-Cu-TPA (1.97),^40^ FJUT-1 (5.17),^57^ Cu^I^@UiO-66-(COOH)_2_ (3.4),^50^ JCM-1 (4.4),^55^ FeNi-M'MOF (1.7),^58^ and Ni(4-DPDS)_2_CrO_4_ (6.7).^48^

For 50/50 and 1/99 C_2_H_2_/C_2_H_4_ binary mixtures, the C_2_H_4_ gas broke through the adsorption bed with high-purity at 39.7 and 41.5 min g^−1^, while the breakthrough time of C_2_H_2_ was longer at 106.2 and 1452.4 min g^−1^, corresponding to breakthrough intervals of 66.5 and 1410.9 min g^−1^, respectively (Fig. 4c and d). The calculated C_2_H_2_ capture capacities were 2.68 and 1.22 mmol g^−1^ for 50/50 and 1/99 mixtures, respectively. The productivity of 99.999% pure C_2_H_4_ was calculated to be 84.6 mmol g^−1^, which is comparable to the benchmark SIFSIX-14-Cu-i (84.6 mmol g^−1^) and higher than those of SIFSIX-3-Zn (1.94 mmol g^−1^), MUF-17 (8.57 mmol g^−1^),^59^ SIFSIX-1-Cu (12.4 mmol g^−1^),^23^ SIFSIX-dps-Cu (14.9 mmol g^−1^),^43^ ZNU-9 (48.57 mmol g^−1^)^39^ and Cu(bpy)NP (20.57 mmol g^−1^).^46^ According to the breakthrough curves, the C_2_H_2_/C_2_H_4_ dynamic selectivity (1/99) of SIFSIX-bidmb-Cu was calculated to be 105.7 (Fig. 4h), notably higher than previously studied sorbents, including SIFSIX-3-Zn (9), SIFSIX-1-Cu (11), and SIFSIX-2-Cu-i (45),^23^ comparable to Ni@FAU (97)^8^ and SIFSIX-14-Cu-i (91),^24^ but lower than ZJU-300a (264).^33^ Similarly, captured C_2_H_2_ can be recovered with high purity (>99% purity) *via* heating the sample at 120 °C after a helium flush at room temperature (Fig. 4g), thus providing a facile approach to obtain pure C_2_H_2_ and C_2_H_4_. Additionally, the breakthrough performance for the 10/90 C_2_H_2_/C_2_H_4_ mixture was investigated at 298 K and 100 kPa with a total gas flow rate of 2 mL min^−1^ (Fig. 4i), resulting in retention times of 15.8 and 216.3 min g^−1^ for C_2_H_4_ and C_2_H_2_, respectively, and a breakthrough time lag of 200.5 min g^−1^. Consecutive breakthrough experiments for 10/90 C_2_H_2_/C_2_H_4_ on SIFSIX-bidmb-Cu′ revealed no performance loss, suggesting recyclability which was supported by PXRD data (Fig. S46†). The breakthrough experiments for 50/50 C_2_H_2_/CO_2_ and 1/99 C_2_H_2_/C_2_H_4_ mixtures conducted at higher gas flow rates (5 mL min^−1^) were in good agreement with those at lower flow rates, demonstrating that the separation performance was unaffected by gas flow rates and highlighting the fast sorption kinetics of C_2_H_2_ (Fig. S47†).

### Binding site identification *via* SCXRD and DFT-D calculations

**Fig. 5:**
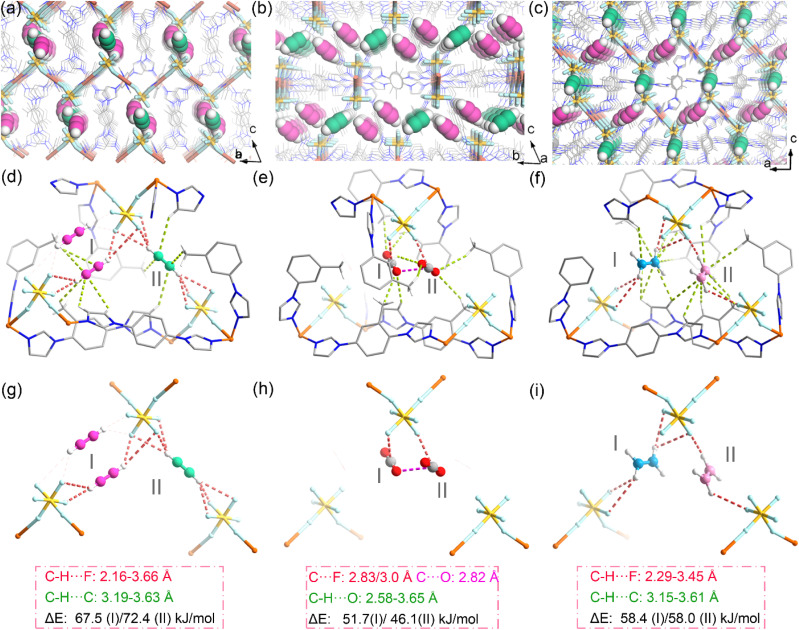
(a–c) The packing of adsorbed C_2_H_2_ molecules in SIFSIX-bidmb-Cu′ viewed in different directions based on SCXRD results. (d–f) The binding sites and configurations of C_2_H_2_ (d), CO_2_ (e) and C_2_H_4_ (f). (g–i) The interactions between SiF_6_^2−^ anions and C_2_H_2_ (g), CO_2_ (h) and C_2_H_4_ (i).

In order to gain insight into the difference in binding affinity between the three sorbates and the framework, the binding sites of SIFSIX-bidmb-Cu′ were explored *via* SCXRD on gas-loaded single crystals in combination with dispersion-corrected density functional theory (DFT-D) calculations (Fig. 5). *In situ* SCXRD studies of SIFSIX-bidmb-Cu′ under 1 bar gas pressure at 298 K using an environmental gas cell were conducted first. In all cases, the gas molecules could not be modeled crystallographically (most likely owing to their high thermal motion at 298 K); however, the most probable locations of the guest molecules within the host framework can be inferred from difference electron density maps (Fig. S48–S52†). In order to further visualize the binding sites, SCXRD studies at 100 K were performed on crystals loaded with C_2_H_2_ and CO_2_ at 298 K. C_2_H_2_@SIFSIX-bidmb-Cu′ was found to exhibit two types of binding sites (site I and site II) for C_2_H_2_ molecules, each located between two adjacent SiF_6_^2−^ anions (Fig. 5a–d and g). The C_2_H_2_ molecule at each site is chelated by two SiF_6_^2−^ anions in an end-on fashion through C≡C–H⋯F hydrogen bonding (*d*_C≡C–H⋯F_: 2.16–3.16 Å), and there are van der Waals (vdW) interactions between C_2_H_2_ and bidmb ligands (*d*_C–H⋯C≡C_: 3.19–3.63 Å) (Fig. 5d and g), affording a zig-zag SiF_6_^2−^···C_2_H_2_(I)⋯SiF_6_^2−^···C_2_H_2_(ii)⋯SiF_6_^2−^ chain along the channel direction (*b*-axis) (Fig. 5a–c). At site I, two parallel and centrosymmetric C_2_H_2_ molecules are simultaneously chelated by two SIFSIX anions, wherein each is unsymmetrically bound to four terminal fluorine atoms from two SiF_6_^2−^ anions (Fig. 5d and g). At site II, the C_2_H_2_ molecule is located at an inversion center and is symmetrically bound to six terminal fluoro atoms from two SIFSIX anions (Fig. S48†).^60^ For the two binding sites, the DFT-D calculated static binding energies (Δ*E*) are ∼67.5 and ∼72.4 kJ mol^−1^, respectively, which are higher than the strongest calculated C_2_H_2_ binding strength in SIFSIX materials (∼52.9 kJ mol^−1^ in SIFSIX-2-Cu-i,^43^ ∼56.0 kJ mol^−1^ in SIFSIX-14-Cu-i^24^ and ∼51.7/56.8 kJ mol^−1^ in UTSA-300a^30^).

For CO_2_@SIFSIX-bidmb-Cu′, the CO_2_ molecules could not be modeled adequately, consistent with the low *Q*_st_ of CO_2_ adsorption. The difference electron density maps obtained from SCXRD studies at 298 K and 100 K on CO_2_-loaded crystals reveal similar guest distribution regions (Fig. S50†). We performed DFT-D calculations to determine the binding sites of CO_2_ and C_2_H_4_. Three CO_2_ binding sites (site I, site II and site III) were identified (Fig. 5e and h). CO_2_ molecules at sites I and II lie in similar positions but with different orientations than that of C_2_H_2_. Each CO_2_ molecule is bound to one (site II and site III) or two (site I) terminal fluorine atoms from a SiF_6_^2−^ anion in a side-on mode through O=C⋯F electrostatic interactions (*d*_O=C⋯F_: 2.86–3.18 Å), accompanied by vdW interactions between CO_2_ and bidmb ligands (*d*_C–H⋯O=C_: 2.50–3.70 Å). The CO_2_ molecules on site I and site II should be half-occupied, as they are too close to the inversion centers of the structure (Fig. S53†). The DFT-D calculated static binding energies (Δ*E*) for the three CO_2_ binding sites were determined to be ∼48.0, ∼45.1 and ∼52.8 kJ mol^−1^, respectively. We attribute this to the lack of synergetic fluorine–CO_2_ binding interactions.

For C_2_H_4_, two binding sites were identified (site I and site II), displaying similar positions and orientations to those of C_2_H_2_ (Fig. 5f and i). On each site, C_2_H_4_ is chelated by SIFSIX pairs in an end-on mode through =C–H⋯F hydrogen bonding (*d*_=C–H_⋯_F_: 2.29–3.45 Å), which is accompanied by vdW interactions between C_2_H_4_ and bidmb ligands (*d*_C–H⋯C=C_: 3.15–3.61 Å). Each SiF_6_^2−^ anion at site I and site II provides two and one free fluoro atoms that bind to one hydrogen atom of C_2_H_4_, respectively. Similarly, the C_2_H_4_ molecule at site II should be half-occupied, as it resides close to the inversion center (Fig. S54†). For the two C_2_H_4_ binding sites, the DFT-D calculated static binding energy (Δ*E*) was determined to be ∼58.0 and ∼58.4 kJ mol^−1^, respectively. We attribute these lower energies to weaker C–H⋯F hydrogen bonds (C_2_H_2_ is more acidic than C_2_H_4_). The C_2_H_2_ binding sites from SCXRD data collected at 100 K and those of CO_2_ and C_2_H_4_ calculated from DFT-D are in good agreement with the guest locations inferred from the difference electron density maps of *in situ* SCXRD at 298 K (Fig. S49–S52†). The DFT-D calculated static binding energy (Δ*E*) is in the sequence of C_2_H_2_ > C_2_H_4_ > CO_2_ and matches well with the order of *Q*_st_ values from sorption isotherms.

In *trans*-SIFSIX HUMs, due to the symmetric coordination mode of the SiF_6_^2−^ anion, the four free (uncoordinated) F sites are equally distributed around the coordination axis. The free rotation of *trans*-SIFSIX anions around the coordination axis will modulate the number of free F sites located on the pore surface at a binding site. For example, in SIFSIX-1-Cu,^23^ only one free F site orients toward the channel, and thus a single C–H⋯F hydrogen bonding was observed (the pore size is large too). When the pore size was reduced by, for instance, interpenetration, the distance between two anions is less (*e.g.*, SIFSIX-2-Cu-i and SIFSIX-14-Cu-i),^23,24^ and dual C–H⋯F hydrogen bonds can occur. When the rotation of *trans* anions results in two free F sites located on the pore surface, each anion can provide two free F sites for one C_2_H_2_, and totally four C–H⋯F hydrogen bonds can be formed (*e.g.* ZJU-300a).^33^ Due to geometry limitations, at most, two of the four F sites of a *trans*-SiF_6_^2−^ anion can be accessible for one C_2_H_2_ molecule. Symmetric bridging of *trans*-SIFSIX anions can result in strong CO_2_ trapping (*e.g.* SIFSIX-3-Ni).^16^ However, for *cis*-SIFSIX anions, the four free F sites are located on the same side of the coordination axis and one C_2_H_2_ molecule can simultaneously engage with three F sites of each anion. We attribute this feature to the highly selective binding of C_2_H_2_*vs.* both CO_2_ and C_2_H_4_ observed herein.

### Water stability tests

**Fig. 6:**
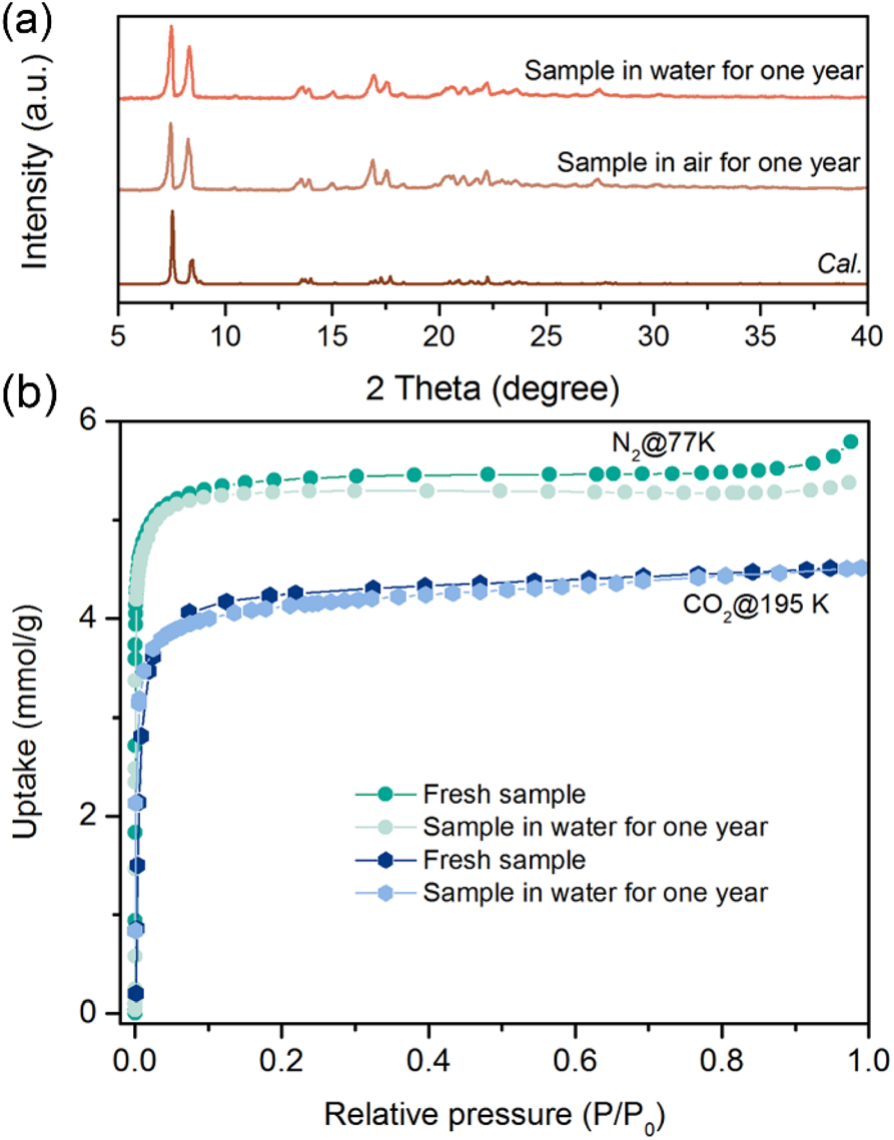
(a) Comparison of the calculated PXRD pattern with those of samples treated in air or water for one year. (b) Comparison of CO_2_ (195 K) and N_2_ (77 K) isotherms of pristine sample with those of samples treated in air or water for one year.

SIFSIX materials sustained by *trans*-SIFSIX anions (*e.g.* SIFSIX-14-Cu-i) have been reported to undergo degradation or phase transformation when exposed to humidity.^61^ Conversely, SIFSIX-bidmb-Cu bulk samples of SIFSIX-bidmb-Cu retained crystallinity after one year of exposure to both liquid water and ambient humidity, as confirmed by PXRD (Fig. 6a) and VT-PXRD (Fig. S55†) experiments. The adsorption isotherms of CO_2_ (195 K), N_2_ (77 K), C_2_H_2_ (298 K), CO_2_ (298 K) and C_2_H_4_ (298 K) of samples exposed to air or water revealed negligible differences *vs.* pristine materials (Fig. 6b, S56 and S57†). Water stability was also demonstrated by consecutive water adsorption–desorption experiments that revealed no uptake loss even after ten cycles and no structural alteration according to PXRD (Fig. S58 and S59†). The high hydrolytic stability of SIFSIX-bidmb-Cu is consistent with that of the first *cis*-SIFSIX sorbent.^34^ The use of imidazole linkers could also be a factor^62^ along with self-catenation.^63^

## Conclusions

This work reveals that *cis*-bridging SIFSIX anions can offer different sorbate binding sites relative to *trans* variants, in this case with high C_2_H_2_ affinity and low CO_2_ affinity. In addition, this is the first example of a *cis*-bridging SIFSIX rigid HUM, and it exhibits C_2_H_2_ molecular traps enabled by multiple fluoro binding sites that exhibit relatively weak CO_2_ and C_2_H_4_ binding. Overall, the combination of high C_2_H_2_ uptake at 0.01 bar, high *Q*_st_ for C_2_H_2_, large difference in *Q*_st_ between C_2_H_2_ and CO_2_, and high C_2_H_2_/CO_2_ and C_2_H_2_/C_2_H_4_ selectivities make SIFSIX-bidmb-Cu′ a leading sorbent for trace C_2_H_2_ capture. This work opens up a new avenue for the design and construction of SIFSIX materials with distinct structural features and pore chemistry. Other fluorinated linkers will be targeted in order to fine-tune C_2_H_2_ binding affinity.

## Data availability

The data supporting this article have been included as part of the ESI.† The crystallographic information can be found in the ESI† associated with this work and at the Cambridge Crystallographic Data Center under deposition numbers 2350654–2350657, 2361231–2361233, *via*https://www.ccdc.cam.ac.uk/data_request/cif, or by emailing data_request@ccdc.cam.ac.uk, or by contacting The Cambridge Crystallographic Data Centre, 12 Union Road, Cambridge CB2 1EZ, UK; fax: +44 1223 336033.

## Author contributions

B. Q.-Song and M. Y. Gao contributed equally to this work and carried out the synthesis, characterization analysis, sorption and separation measurements, and writing – original draft; C.-H. Deng and Q.-Y. Yang assisted with the gas sorption measurement and data analysis; S.-Q. Wang and S. Darwish assisted with the characterization analysis; L. M. van Wyk, A. C. Eaby and L. J. Barbour conducted the *in situ* single crystal X-ray diffraction measurements; D. Li and S.-J. Qin performed computational simulations; B.-Q. Song, Y. -L. Peng and M. J. Zaworotko carried out the methodology, supervision, and writing – review & editing; all authors contributed to preparing the manuscript.

## Conflicts of interest

The authors declare no competing financial interests.

## Supplementary Material

SC-OLF-D5SC00697J-s001

SC-OLF-D5SC00697J-s002
