# Supplementary material for: A new type of C2H2 binding site in a cis-bridging hexafluorosilicate ultramicroporous material that offers trace C2H2 capture
Source: Chem Sci. 2025 Apr 23;16(20):9010–9. doi: 10.1039/d5sc00697j (PMC12015180; doi:10.1039/d5sc00697j)
Supplement: SC-016-D5SC00697J-s001 [file SC-016-D5SC00697J-s001.pdf]

Supplementary Information (SI) for Chemical Science.
This journal is © The Royal Society of Chemistry 2025

Supporting Information

A new type of C_2H_2 binding site in a *cis*-bridging hexafluorosilicate ultramicroporous material that offers trace C_2H_2 capture

Bai-Qiao Song,[#,*,a] *Mei-Yan Gao,*[#,b] *Lisa Mercene van Wyk,*[c] *Cheng-Hua Deng,*[b] *Alan C. Eaby,*[c] *Shi-Qiang Wang,*[b] *Shaza Darwish,*[b] *Dan Li,*[a] *Shao-Jie Qin,*[a] *Yun-Lei Peng,*[*,d] *Qing-Yuan Yang,*[e] *Leonard J. Barbour,*[c] *Michael J. Zaworotko*[*,b]

[a]College of Materials and Chemistry & Chemical Engineering, Chengdu University of Technology, Chengdu 610059, China
[b]Department of Chemical Sciences and Bernal Institute, University of Limerick, Limerick V94 T9PX, Republic of Ireland
[c]Department of Chemistry and Polymer Science, University of Stellenbosch, Matieland 7602, South Africa
[d]Department of Applied Chemistry, College of Science, China University of Petroleum-Beijing, Beijing 102249, China
[e]School of Chemical Engineering and Technology, Xi'an Jiaotong University, Xi'an 710049, China

Table of Contents

1. Materials and Methods

2. Experimental Section

3. Supporting Figures

4. Supporting Tables

5. Supporting References

1. Materials and Methods

The ligand 1,4-bis(1-imidazol-yl)-2,5-dimethyl benzene (bidmb) was synthesized using the analogous procedures for 1,4-bis(1-imidazolyl)benzene based on the literature method.[1] Other reagents and solvents were commercially available and used without further purification.

2. Experimental Section

2.1 Synthesis of SIFSIX-bidmb-Cu

$[CuSiF_6(bidmb)_2]_n$. (SIFSIX-bidmb-Cu) First, a 5 mL water solution containing $CuSiF_6 \cdot 6H_2O$ (22 mg, 0.1 mmol) was added into the bottom of a glass tube (diameter: 15 mm and length: 150 mm). And then, a 10 mL MeOH and water mixture (1:1, v:v) was layered onto the water solution as a buffer layer. Finally, a 10 mL MeOH solution of bidmb (48 mg, 0.2 mmol) was carefully layered on the buffer solution. The tube was tightly sealed and stood at room temperature for one month. After that, block-shaped purple crystals will be formed on the glass tube wall, which were isolated by filtration and washed with H_2O and methanol for multiple times. The obtained sample was dried in air at room temperature overnight. Yield: 32% based on bidmb.

2.2 Single-crystal X-ray diffraction measurements (SCXRD)

a. Structure determination of SIFSIX-bidmb-Cu

The single-crystal diffraction data for SIFSIX-bidmb-Cu was collected on a Bruker Quest diffractometer equipped with a CMOS detector and IμS microfocus X-ray source (Mo Kα, λ = 0.71073 Å). The data was indexed, integrated and scaled in APEX3.[2] Absorption correction was performed by multi-scan method using in SADABS.[3] Space group was determined using XPREP[4] implemented in APEX3. Structure was solved using intrinsic phasing method (SHELXT)[5] and refined on F^2 using nonlinear least-squares techniques with SHELXL[6] programs incorporated in OLEX2 graphical user interface.[7] Anisotropic thermal parameters were applied to all non-hydrogen

atoms. The reported refinement for SIFSIX-bidmb-Cu was of the guest-free structure using the *.hkl file produced using the SQUEEZE[8] routine because the guests are highly disordered in the framework. The void volume (excluding solvent guest molecules) in the crystal cell was calculated using the program PLATON.[9]

b. *In -situ* variable pressure SCXRD at 298 K

(I) Activation Procedure

Single crystals of the as-synthesised material of appropriate size, possessing suitable morphology and ability to extinguish plane polarized light were glued onto a glass fibre with clear epoxy. The glass fibre was then attached to an environmental gas cell (EGC) consisting of a 0.3 mm glass Lindemann Capillary set into a steel nut with epoxy which is then screwed into a valve body. The EGC allows for pressurisation/evacuation of the immediate crystal environment while the valve allows for this environment to be isolated and transported to the diffractometer.

Once placed inside the environmental gas cell the crystal was then connected to a turbo-vacuum pump (pressure: ~3 x 10^{-3} millibar) while immersed in oil which was heated to 120 °C for approximately 72 hours. The valve was then closed and the EGC removed from the apparatus.

(II) Apohost structure (SIFSIX-bidmb-Cu') determination

For the apohost data collection the crystal (in an EGC) was placed on the diffractometer while under vacuum. X-ray intensity data were recorded on a Bruker SMART APEX II diffractometer. The instrument is equipped with Incoatec IμS molybdenum (λ = 0.71073 Å) microfocus X-ray source and a CCD area detector. The diffractometer is fitted with a 700 Series Cryostream Plus cryostat from Oxford Cryosystems, which is used to control the sample temperature at 298 K. Omega and phi scans were carried out in a high redundancy strategy.

Data reduction and absorption corrections were carried out using the SAINT and SADABS programs,[10] respectively. The unit-cell dimensions were refined on all data and space groups were assigned based on systematic absences and intensity statistics. The structures were solved with a dual-space algorithm using SHELXT.[5] Structure refinement was carried out with SHELXL 2018/1[6] using the X-Seed graphical user

interface.[11] Non-hydrogen atoms were refined anisotropically. Hydrogen atoms were placed in calculated positions. Where necessary thermal parameters were restrained using the RIGU and SIMU cards. The disorders of bidmb and fluorine atoms of SiF_6^{2-} anions were observed. Consequently, these disordered fragments were split into two positions where the occupancy of each is fixed to be 0.5.

(III) Gas Loading

For the C_2H_2 loaded structure an activated crystal in an EGC was attached to a C_2H_2 cylinder with a regulator. The system was pressurised to 1 bar and allowed to equilibrate for between 16 and 24 hours. After which the valve to the ECG was closed and it could be transported to the diffractometer. For the CO_2 and C_2H_4 loaded structures the EGC was attached to a pressure manifold, which was in turn connected to the appropriate gas cylinder. The system was pressurised to 1 bar and allowed to equilibrate for between 16 and 24 hours.

(IV) Determination of Gas Loaded Structures at 298 K

The data collection, and structure solution and refinement for the gas loaded structures at 298 K, were carried out similarly to the apohost material. In all cases it was not possible to model the guest molecules adequately (most likely owing to high thermal motion at ambient temperatures). To locate the most probable positions of the guests, electron density maps were generated in Olex2 for both materials. Electron density maps were generated as surfaces utilising the difference function with a resolution of 0.1 Å at a level of 1.0 or 1.2 $eÅ^{-3}$ (to obtain the map of highest clarity). Areas of positive electron density (in this case mainly attributed to the guests) are shown in green, while areas of negative electron density (which may arise from heavy element shielding effects) are shown in red. Hydrogen atoms were omitted for clarity when rendering these images. The reported refinement for gas@SIFSIX-bidmb-Cu'_in-situ was of the guest-free structure using the *.hkl file produced using the SQUEEZE routine because the gas molecules are highly disordered in the framework (Table S1). The obtained structures were labeled as C_2H_2@SIFSIX-bidmb-Cu'_in-situ, C_2H_4@SIFSIX-bidmb-Cu'_in-situ, CO_2@SIFSIX-bidmb-Cu'_in-situ (Table S1).

c. Structure determination of C_2H_2 and CO_2 loaded structures at 100 K

Thanks to the high thermal motion of gas molecules inside the channel, in-situ SCXRD studies at 298 K cannot identify the precise positions of the gas molecules. Then, we tried to perform the SCXRD tests at 100 K which may improve the data quality as low temperature can freeze the thermal motion of gas molecules. However, in-situ SCXRD at 100 K is not feasible because the crystals inside the EGC (environmental gas cell) are under 1 bar gas atmosphere, and lowering the temperature of EGC from 298 K to 100 K will change the state of the surrounded gas (liquefaction or solidification) and the gas uptake of crystals. One solution is to load the gas into the structure at 298 K and subsequently to pick up gas-loaded crystals for SCXRD studies at 100 K. For this purpose, fresh crystals of SIFSIX-bidmb-Cu were first activated at 120 °C under high vacuum to obtain the totally dried form, SIFSIX-bidmb-Cu'. And then, the activated crystals were exposed to 1 bar C_2H_2 or CO_2 gas at 298 K for 3-6 hours. After that, the gas-loaded crystals were quickly picked and transferred to diffraction instrument for data collection at 100 K.

For gas-loaded single crystals, the single-crystal diffraction data were collected at 100 K using Cu Kα radiation (λ = 1.54184 Å) on a Rigaku XtaLAB Synergy-DW diffractometer equipped with a HyPix-Arc 150° detector. Diffraction data were acquired and processed with CrysAlisPro software package. Direct or structure expansion methods were used for all structures, and the refinements were established by full-matrix least squares with SHELX-2018/3[6] using X-seed[11] and Olex2[7] software as a graphical interface. The obtained structures are labeled as C_2H_2@SIFSIX-bidmb-Cu' and CO_2@SIFSIX-bidmb-Cu'. For CO_2@SIFSIX-bidmb-Cu', the CO_2 molecules were in disorder even at 100 K and cannot be modeled adequately, further proving the weak binding of CO_2 to the framework. Electron density maps were generated as surfaces utilising the difference function with a resolution of 0.1 Å at a level of 0.7 $eÅ^{-3}$ (to obtain the map of highest clarity). Areas of positive electron density (in this case mainly attributed to the guests) are shown in green, while areas of negative electron density (which may arise from heavy element shielding effects) are shown in red. Hydrogen atoms were omitted for clarity when rendering these images. The reported refinement for CO_2@SIFSIX-bidmb-Cu' was of the guest-free structure using

the *.hkl file produced using the SQUEEZE routine because the CO_2 molecules are highly disordered in the framework (Table S1).

Crystallographic data and structural refinement information are listed in Tables S1. Crystallographic data for the structure reported in this paper have been deposited with the Cambridge Crystallographic Data Centre as CCDC numbers 2350654 (SIFSIX-bidmb-Cu), 2350655 (SIFSIX-bidmb-Cu'), 2350656 (C_2H_2@SIFSIX-bidmb-Cu'), 2350657 (CO_2@SIFSIX-bidmb-Cu'), 2361231 (C_2H_2@SIFSIX-bidmb-Cu'_in-situ), 2361232 (C_2H_4@SIFSIX-bidmb-Cu'_in-situ) and 2361233 (CO_2@SIFSIX-bidmb-Cu'_in-situ) (available free of charge, on application to the CCDC, 12 Union Rd., Cambridge CB2 1EZ, U.K.; e-mail deposit@ccdc.cam.ac.uk).

2.3 IR spectra

Fourier Transform Infrared (FTIR) Spectroscopy A drop of peptide hydrogel (2 wt%) was dropped onto the ATR plate by syringe delivery. Afterwards, the droplet was allowed to be completely air dry. Spectra were obtained by using a FTIR spectrometer (Agilent technologies, Cary 630) in the range of 4000-450 cm^{-1}.

2.4 Thermogravimetric analysis (TGA)

Thermogravimetric analyses (TGA) were performed under N_2 atmosphere using a TA Instruments Q50 system. Samples were loaded into aluminum sample pans and heated at 5 K min^{-1} from room temperature to 550 ^{o}C.

2.5 Powder X-ray diffraction measurements

Powder X-ray diffraction patterns were recorded on a PANalytical X'Pert MPD Pro (Cu Ka, λ = 1.5418 Å) with a 1D X'Celerator strip detector. Experiments were conducted in continuous scanning mode with the goniometer in the theta-theta orientation. Incident beam optics included the Fixed Divergences slit with anti-scatter slit PreFIX module, with a 1/8° divergence slit and a 1/4° anti-scatter slit, as well as a 10 mm fixed incident beam mask and a Soller slit (0.04 rad). Divergent beam optics included a P7.5 anti-scatter slit, a Soller slit (0.04 rad), and a Ni β filter. The data were

collected in the range of 2θ = 3-50. Raw data was then evaluated using the X'Pert HighScore Plus™ software V 4.1 (PANalytical, The Netherlands).

2.6 *In-situ* variable temperature powder X-ray diffraction (VT-PXRD)

Diffractograms at different temperature were recorded using a PANalytical X'Pert Pro-MPD diffractometer equipped with a PIXcel3D detector operating in scanning line detector mode with an active length of 4 utilizing 255 channels. Anton Paar TTK 450 stage coupled with the Anton Paar TCU 110 Temperature Control Unit was used to record the variable temperature diffractograms. The diffractometer is outfitted with an Empyrean Cu LFF (long fine-focus) HR (9430 033 7300x) tube operated at 40 kV and 40 mA and CuKα radiation (λ_α = 1.54056 Å) was used for diffraction experiments. Continuous scanning mode with the goniometer in the theta-theta orientation was used to collect the data. Incident beam optics included the Fixed Divergences slit, with a 1/4° divergence slit and a Soller slit (0.04 rad). Divergent beam optics included a P7.5 anti-scatter slit, a Soller slit (0.04 rad), and a Ni-β filter. In a typical experiment, 50 mg of sample was ground into a fine powder, and was loaded on a zero-background sample holder made for Anton Paar TTK 450 chamber. The data was collected from 5°-45° (2θ) with a step-size of 0.0167113° and a scan time of 50 seconds per step. Crude data were analyzed using the X'Pert HighScore Plus™ software V 4.1 (PANalytical, The Netherlands).

2.7 *In-situ* variable temperature variable pressure PXRD

The crystalline material was ground into a fine powder using a pestle and mortar, and packed into an environmental gas cell (EGC). The EGC consists of a 0.5 mm glass Lindemann Capillary attached to a steel nut with epoxy, which is then screwed into a valve body. The EGC allows for pressurisation/evacuation of the immediate sample environment while the valve allows for this environment to be isolated and transported to the diffractometer.

Each material was activated *in situ* by connecting the EGC to a turbo-vacuum pump (pressure: ~5 x 10-4 millibar) while also immersed in oil, which was heated to 120 °C

for approximately 18 hours. The valve was then closed and the EGC removed from the apparatus.

For the pressure studies an EGC was attached to a C_2H_2 cylinder via a regulator. The system was pressurised and left to equilibrate under static pressure for approximately 6–8 hours. After the valve to the ECG was closed, it could be transported to the diffractometer.

A PANalytical XPERT-PRO diffractometer was used to record experimental diffractograms. The diffractometer utilises Bragg-Brentano geometry and Cu Kα radiation (λ = 1.5418 Å) as the incident beam. Intensity data were recorded using a capillary spinner to which the EGC was attached. The samples were scanned between 4° and 45° 2θ with a varying scan speed and step size, which was dependent on the nature of the sample. The diffractometer is fitted with a 700 Series Cryostream Plus cryostat from Oxford Cryosystems, which is used to control the sample temperature. For the low temperature diffractograms, the system was allowed to equilibrate for at least an hour at 273 K before measurement.

2.8 Low-pressure gas adsorption measurements

The sorption isotherms for N_2 at 77 K and CO_2 at 195 K were measured using Micromeritics Tristar II 3030 instrument. Before gas sorption experiment, the freshly prepared sample of SIFSIX-bidmb-Cu was activated in the quartz tube and degassed under high vacuum at 120 °C for 6 hours to remove the remnant solvent molecules prior to measurements. The surface area was determined from the N_2 adsorption isotherm of SIFSIX-bidmb-Cu' collected at 77 K by applying the Langmuir and Brunauer-Emmett-Teller (BET) models.

The sorption isotherms for C_2H_2, C_2H_4 and CO_2 at 273 K and 298 K were measured using Micromeritics 3 Flex surface area and pore size analyser. A Julabo temperature controller was used to maintain a constant temperature in the bath through the duration of the experiment. Samples were degassed under high vacuum at 120 °C for 6 hours on a Smart VacPrep instrument prior to the analysis.

2.9 Calculation of the Langmuir surface area

The well-known Langmuir isotherm model can be expressed by the following equation:

$$\frac{Q}{Q_0} = \frac{bP}{1 + bP}$$

Where Q (cm^{-3} g^{-1}) is the amount adsorbed; Q_0 (cm^3 g^{-1}) is the saturated amount adsorbed; P (mmHg) is the equilibrium pressure; and b (mmHg^{-1}) is the adsorption affinity.

A line expression for the Langmuir equation can be written as following:

$$\frac{P}{Q} = \frac{1}{bQ_0} + \frac{1}{Q_0}P$$

A least-squares fit is performed on the ($\frac{P}{Q}, P$) designated pairs where $\frac{P}{Q}$ is the independent variable and P is the dependent variable. The following are calculated:

a) Slope ($\frac{1}{Q_0}$, g cm^{-3} STP)

b) Y-intercept ($\frac{1}{bQ_0}$, g·mmHg cm^{-3} STP)

c) Error of the slope (g cm^{-3} STP)

d) Error of the y-intercept (g·mmHg cm^{-3} STP)

Using the results of the above calculations, the Langmuir surface area can be calculated as following:

$$S_g = A_m \times N_A \times \frac{Q_0}{22414} \times 10^{-18}$$

Where S_g is the Langmuir surface area (m^2 g^{-1}); A_m = molecular cross-sectional area

(nm^2) of adsorbate i.e. 0.1700 nm^2 for CO_2, and N_A = 6.02 × 10^{23}.

2.10 Calculation of the Brunauer-Emmett-Teller (BET) surface area

The well-known Brunauer, Emmett, and Teller (BET) isotherm model can be expressed by the following equation:

$$\frac{P}{Q(P_0 - P)} = \frac{1}{CQ_0} + \frac{C-1}{CQ_0} * \frac{P}{P_0}$$

Where Q (cm^{-3} g^{-1}) is the amount adsorbed; Q_0 (cm^3 g^{-1}) is the saturated amount adsorbed; P is the equilibrium pressure and P_0 is the saturated vapor pressure under measurement temperature, and C is a constant relating to the sorption ability of sorbate. A line expression for the BET equation can be written as following:

$$\frac{1}{Q(\frac{1}{P/P_0} - 1)} = \frac{1}{CQ_0} + \frac{C-1}{CQ_0} * \frac{P}{P_0}$$

A least-squares fit is performed on the ($\frac{1}{Q(\frac{1}{P/P_0} - 1)}, \frac{P}{P_0}$) designated pairs where $\frac{1}{Q(\frac{1}{P/P_0} - 1)}$ is the independent variable and $\frac{P}{P_0}$ is the dependent variable. The following are calculated:

e) Slope ($\frac{C-1}{CQ_0}$, g cm^{-3} STP)

f) Y-intercept ($\frac{1}{CQ_0}$, g cm^{-3} STP)

g) Error of the slope (g cm^{-3} STP)

h) Error of the Y-intercept (g cm^{-3} STP)

i) $Q_0 = 1/(Slope + Y - Intercept)$

Using the results of the above calculations, the Langmuir surface area can be calculated

as following:

$$S_{BET} = A_m \times N_A \times \frac{Q_0}{22414} \times 10^{-18}$$

Where S_{BET} is the Langmuir surface area (m^2 g^{-1}); A_m = molecular cross-sectional area (nm^2) of adsorbate i.e., 0.1700 nm^2 for CO_2, and $N_A = 6.02 \times 10^{23}$.

2.11 Dynamic Breakthrough Experiments

In typical breakthrough experiments, ~0.80 g of finely grinded sample was first placed in a quartz tube (8 mm diameter) to form a fixed bed. Then, the adsorbent bed was purged under a 20 mL min^{-1} flow of He gas at 120 °C for 6 hours to activate the sample prior to breakthrough experiments. Upon cooling to room temperature, a binary gas mixture (C_2H_2/CO_2 or C_2H_2/C_2H_4) at a total flow rate of 1 mL min^{-1} (50:50, v:v) or 1.5 mL min^{-1} (1:99, v:v) or 2 mL min^{-1} (10:90, v:v) was introduced into the activated column bed to perform breakthrough experiments at 25 °C and 1 bar. The outlet composition was continuously monitored by a mass spectrometer (Hiden HPR20) or gas chromatography (Shimadzu GC2030 with an FID detector) until complete breakthrough was achieved. Once the breakthrough experiment finished, the inlet gas was immediately switched from reaction mixture to He flow at a flow rate of 20 ml/min for a period of time before the packed column bed was heated to 120 ºC to desorb the adsorbed gas. The packed column bed was regenerated at 120 ºC with constant He flow (20 mL min^{-1}) over night to ensure complete sample regeneration. The outlet composition was also continuously monitored by a mass spectrometer (Hiden HPR20) or gas chromatography (Shimadzu GC2030 with an FID detector) until complete regeneration of sample was achieved.

2.12 Separation factor / Separation selectivity calculations.

The amount of adsorbed specific gas i (q_i) is calculated from the breakthrough curve as follows:

$$q_i = \frac{F_i t_0 - V_{dead} - \int_0^{t_0} F_{ei} dt}{m}$$

Here, F_i is the influent flow rate of gas i (cm^3 min^{-1}), F_{ei} is the effluent flow rate of gas i (cm^3 min^{-1}), V_{dead} is the dead volume of the system (cm^3), t_0 is the adsorption time (min) and m is the mass of the sorbent (g).[12]

On approximation, this simplifies to:

$$q_i = \frac{F_T \Delta T P_i}{m}$$

F_T is the total flow rate of gas (cm^3 min^{-1}), P_i is the partial pressure of gas i (bar) and ΔT is the time for initial breakthrough of gas i to occur (min).[13] The separation factor, also known as separation selectivity (α_{AC}) for the breakthrough experiment i.e. breakthrough derived selectivity is determined as follows:

$$\alpha = \frac{q_1 y_2}{q_2 y_1}$$

y_i is the partial pressure of gas i in the gas mixture. In the case where one gas component has negligible adsorption, the amount of gas adsorbed is treated as ≤ 1 cm^3 for calculations.

2.13 Calculation of adsorption enthalpies.

A virial-type expression of the following form was used to fit the isotherm data collected at 273 and 298 K for C_2H_2, CO_2 and C_2H_4.

$$lnP = lnN + 1/T \sum_{i=0}^{m} a_i N^i + \sum_{j=0}^{n} b_j N^j$$

Here, P is the pressure described in Pa, N is the adsorbed amount in mmol/g, T is the temperature in K, a_i and b_j are virial coefficients, and m, n are the number of coefficients used to describe the isotherms.

The Q_{st} in the range of adsorption capacity can be calculated through the Clausius-Clapeyron equation, which is as follows:

$$Q_{st} = - R\left[\frac{\partial lnP}{\partial\left({}^{1}/_{T}\right)}\right]_{n}$$

Where T is temperature (in K) and R is the ideal gas constant (8.314 J mol^{-1} K^{-1}). Consequently, combining the Clausius-Clapeyron equation and Virial expression, the following expression of Q_{st} can be obtained:

$$Q_{st} = - R\sum_{i=0}^{m} a_{i}N^{i}$$

The values of virial coefficients a0 to am were then used to calculate the isosteric heat of adsorption using the above expression.

2.14 Calculation of IAST selectivity

The selectivities for the adsorbate mixture composition of interest were predicted from the single-component adsorption isotherms using Ideal Adsorbed Solution Theory (IAST)[14]. First, the single-component isotherms for the adsorbates at 298 K were fitted to the dual-site Langmuir equation:

$$n(P) = \frac{n_{m1}b_{1}P}{1 + n_{m1}b_{1}P} + \frac{n_{m2}b_{2}P}{1 + n_{m2}b_{2}P}$$

In this equation, *n* is the amount adsorbed per mass of material (mmol g^{-1}), *P* is the total pressure (Pa) of the bulk gas at equilibrium with the adsorbed phase, $nm1$ and $nm2$ are the saturation uptakes (mmol g^{-1}) for sites 1 and 2, $b1$ and $b2$ are the affinity coefficients (Pa^{-1}) for sites 1 and 2.

Next, the spreading pressure for adsorbates i and j can be calculated using the following equations:

$$\frac{\pi_{i}^{0}A}{RT} = \int_{0}^{P_{i}^{0}(\pi)} \frac{n_{i}(P)}{P}dP$$

$$\frac{\pi_{j}^{0}A}{RT} = \int_{0}^{P_{j}^{0}(\pi)} \frac{n_{j}(P)}{P}dP$$

In the above equations, A is the specific surface area (assumed to be the same for all

adsorbates), R is the ideal gas constant, T is the temperature in K, and $P^{\circ}_i(\pi)$ and $P^{\circ}_j(\pi)$ are the equilibrium gas phase pressures corresponding to the solution temperature and solution spreading pressure for the adsorption of pure components i and j, respectively. Further, the following equations hold true for a two-component mixture according to IAST:

$$\pi_I^0 = \pi_J^0$$

$$Py_i = P_i^0 x_i$$

$$Py_j = P_j^0 x_j$$

$$x_i + x_j = 1$$

$$y_i + y_j = 1$$

Here, x_i and x_j are the mole fractions of components i and j in the adsorbed phase, respectively, and y_i and y_j are the mole fractions of components i and j in the gas phase, respectively. The previous seven equations are seven independent equations with nine unknown variables. In order to solve for all of the unknowns, two quantities must be specified, particularly P and y_i. Utilization of the aforementioned equations yields the following equilibrium expression for adsorbates i and j:

$$\int_0^{\frac{Py_i}{x_i}} \frac{n_i(P)}{P} dP = \int_0^{\frac{P(1-y_i)}{(1-x_i)}} \frac{n_j(P)}{P} dP$$

The above equation was solved for x_i for a range of pressures at a specified y_i value. Finally, the selectivity for adsorbate i relative to adsorbate j was calculated using the following expression:

$$S_{i/j} = \frac{x_i y_j}{x_j y_i}$$

2.15 Water sorption and hydrolytic stability tests

Dynamic water vapor sorption isotherms were collected using ca. 9 mg of SIFSIX-bidmb-Cu using a DVS Adventure system from Surface Measurement Systems. Before

the measurements, the sample was first activated *in situ* at 120 °C until no weight change was observed for a period of time. During the measurements, temperature was maintained at 298 K by enclosing the system in a temperature-controlled incubator. The mass of the sample was determined in parallel by comparison to an empty reference chamber and recorded by a high-resolution microbalance with a precision of 0.1 μg. Isotherms were measured from 0 to 95% R.H. with an equilibrium criterion dm/dt =0.05 %/min. The minimum and maximum equilibration times for each step were 10 and 360 min.

The crystallinity stability of SIFSIX-bidmb-Cu was evaluated by PXRD measurements after synthesis, activation, cycling experiments, exposure in air and immersion in water for one year. Retention of porosity after exposure in air and water was evaluated by 195 K CO_2, 77 k N_2, 298 K $C_2H_2/CO_2/C_2H_4$ sorption isotherms collected on SIFSIX-bidmb-Cu'.

2.16 Computational Methods

Dispersion-corrected density functional theory (DFT-D) calculations have been performed by using the generalized gradient approximation (GGA)[15] with a Perdew-Burke-Ernzerh (PBE) as the exchange-correlation functional implemented in the CASTEP package[16]. The projected augmented wave generalized gradient approximation (PAWGGA) method was adopted to describe the electron-ion interaction. Wave functions were expanded using a planewave basis set with an energy cutoff of 670 eV, the electronic threshold was set to 10^{-6} eV. The structure was optimized aiming at the global energy minimum, and fully relaxed until the residual force convergence value on each atom is less than 0.03 eV Å^{-1}, and the Gamma-point was taken into account during the numerical integration over the Brillouin zone. We first optimized the structure of SIFSIX-bidmb-Cu'. C_2H_4 gas molecule was then introduced to the optimized host structure at the experimentally identified adsorption site (from the difference electron density map), followed by a full structural relaxation. To obtain the gas binding energy, an isolated gas molecule placed in a supercell (with the same cell dimensions as the sorbent crystal) was also relaxed as a reference. The

static binding energy (at $T = 0$ K) was then calculated using $\Delta EB = E(MOF) + E(gas) - E(MOF + gas)$.

3. Supporting Figures

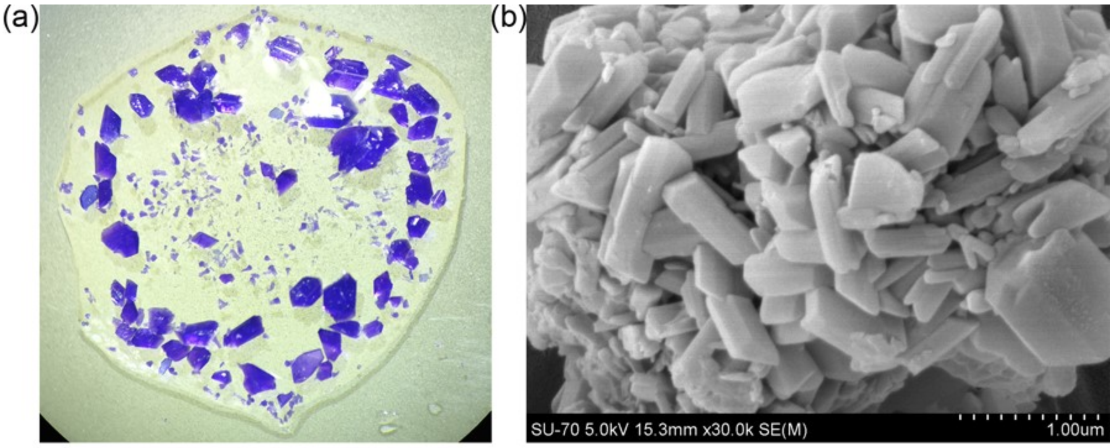

Figure S1 (a) The photograph of single crystals synthesized via diffusion. (b) The scanning electron microscope (SEM) image of microcrystalline powder sample obtained via fast mixing.

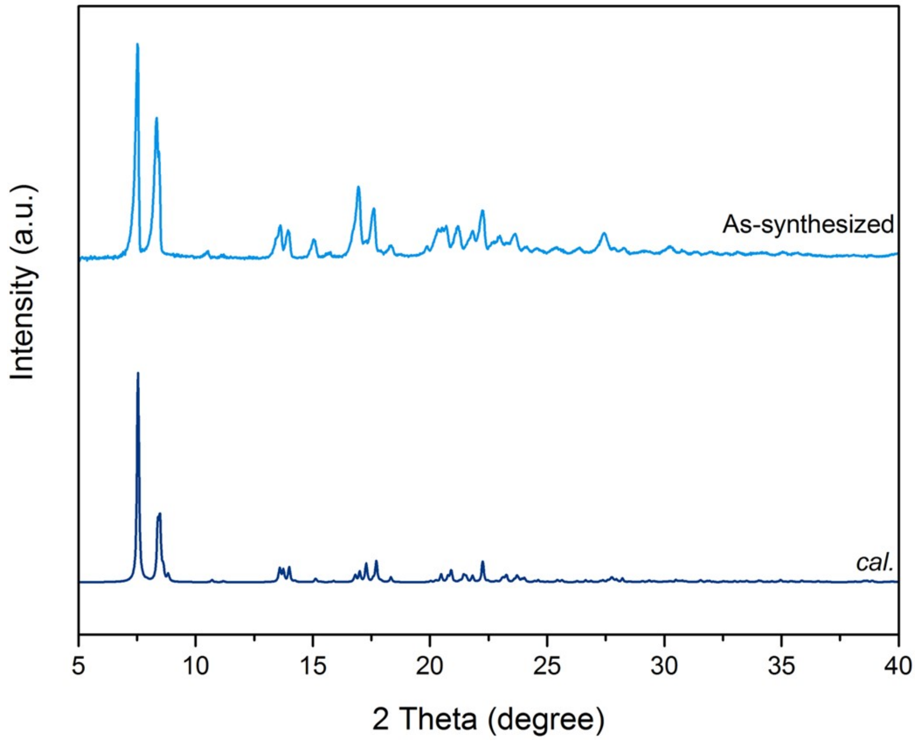

Figure S2 Comparison of PXRD patterns of as-synthesized microcrystalline powder sample and the one calculated from the single crystal structure of SIFSIX-bidmb-Cu.

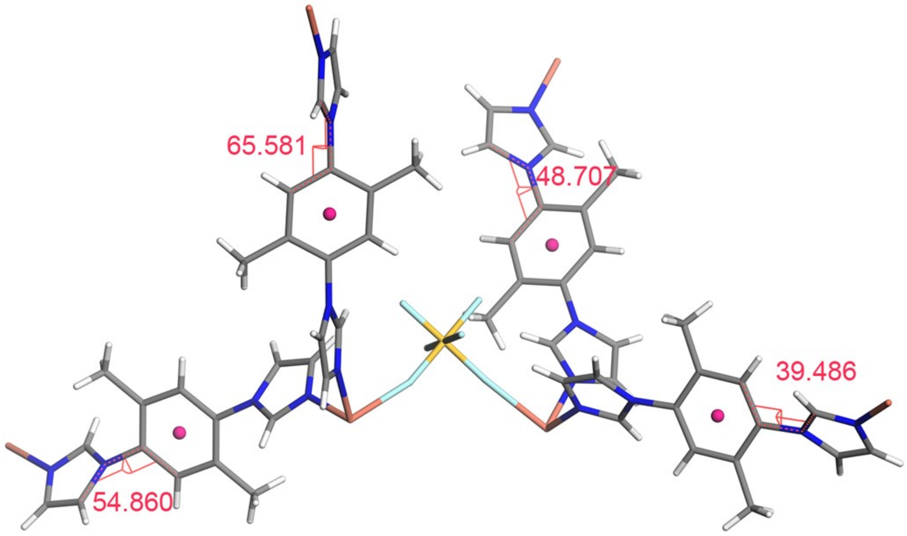

Figure S3 The coordination geometry in SIFSIX-bidmb-Cu. The small balls on the middle of phenyl rings on bidmb ligands represent the inversion centers. Clearly, all the bidmb ligands in SIFSIX-bidmb-Cu is centrosymmetric, and thus, all the ligands adopt the anti-conformation, which is the reason why the Cu(bidmb)$_2$ sql net is coplanar. The dihedral angle between imidazolyl plane and phenyl plane is labeled, which is varying with different molecules.

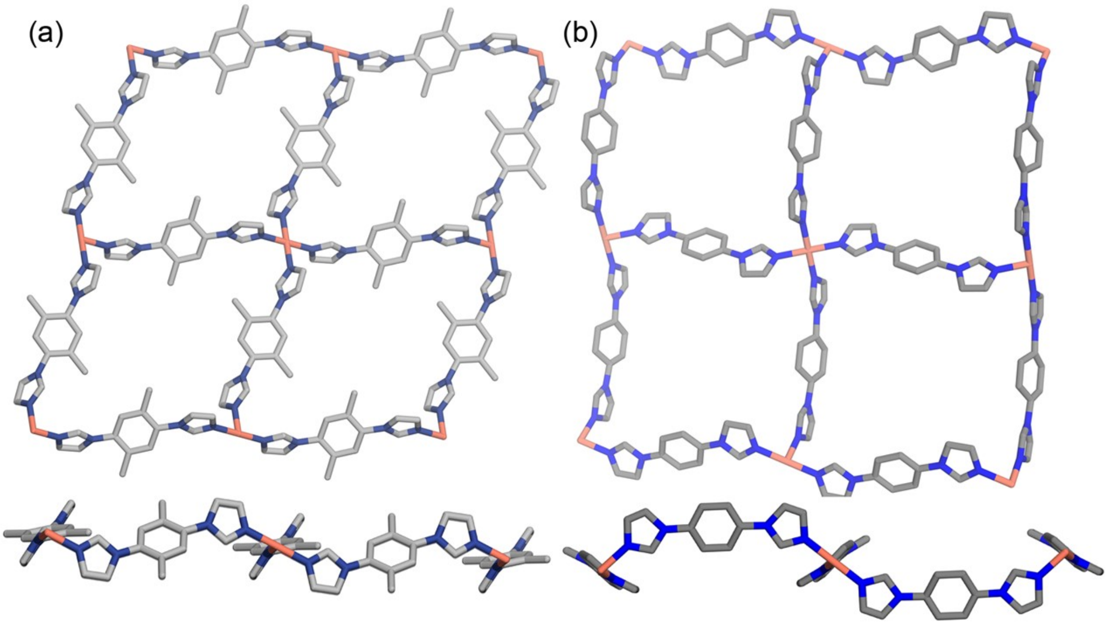

Figure S4 (a) The single sql net observed in SIFSIX-bidmb-Cu, top and side view (top and bottom). (b) The undulating sql net observed in SIFSIX-23-Cu, top and side view (top and bottom). The undulating sql net is ascribed to the existence of cis-conformational bi-imidazolyl ligands.

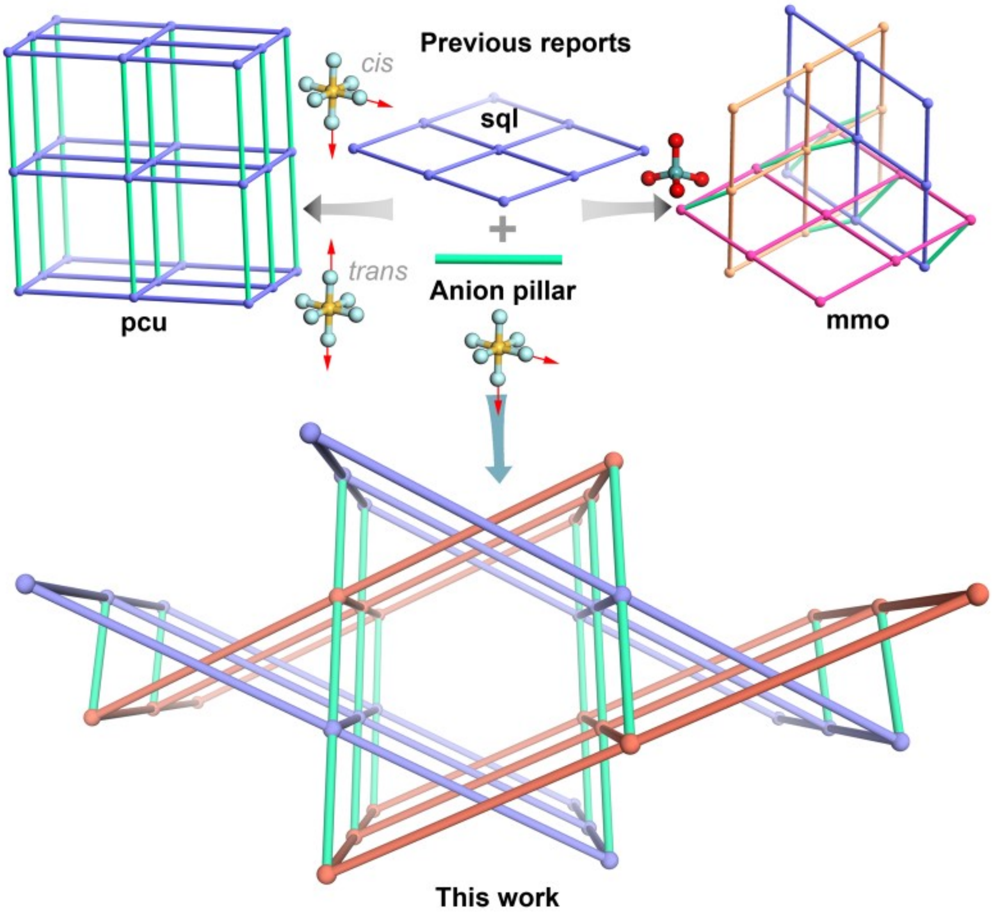

Figure S5 Comparison of different hybrid coordination networks assembled by inorganic anions pillaring **sql** nets. Usually the inorganic anions are: octahedral anions MF_6^{2-} (M = Si, Ti, Sn…) and $NbOF_5^{2-}$; tetrahedral anions XO_4^{2-} (X = S, W, Mo, Cr…). For octahedral anions, there are two different bridging coordination modes: *trans* (two opposite F atoms participation in coordination, i.e., linear pillar) and *cis* (two adjacent F atoms participation in coordination, i.e., angular pillar). The *trans*-bridging coordination modes have long been used to pillar **sql** nets to form traditional **pcu** networks (e.g. SIFSIX materials).Up to date, only one flexible SIFSIX material (SIFSIX-23-Cu and its analogs) containing the *cis*-SIFSIX anion pillar was reported. The *cis*-bridging coordination mode of octahedral anions resembles that of tetrahedral anions that can pillar interpenetrated **sql** nets to form stable hybrid ultramicroporous materials (HUMs), like **mmo** nets from WO_4^{2-} and MoO_4^{2-}. Taking advantage of the angular feature of *cis*-SiF_6^{2-} anions, in this work, we reported the synthesis and separation properties of SIFSIX-bidmb-Cu, the second SIFSIX material composed of *cis*-SiF_6^{2-} anions but the first rigid framework assembled from *cis*-SiF_6^{2-} anions.

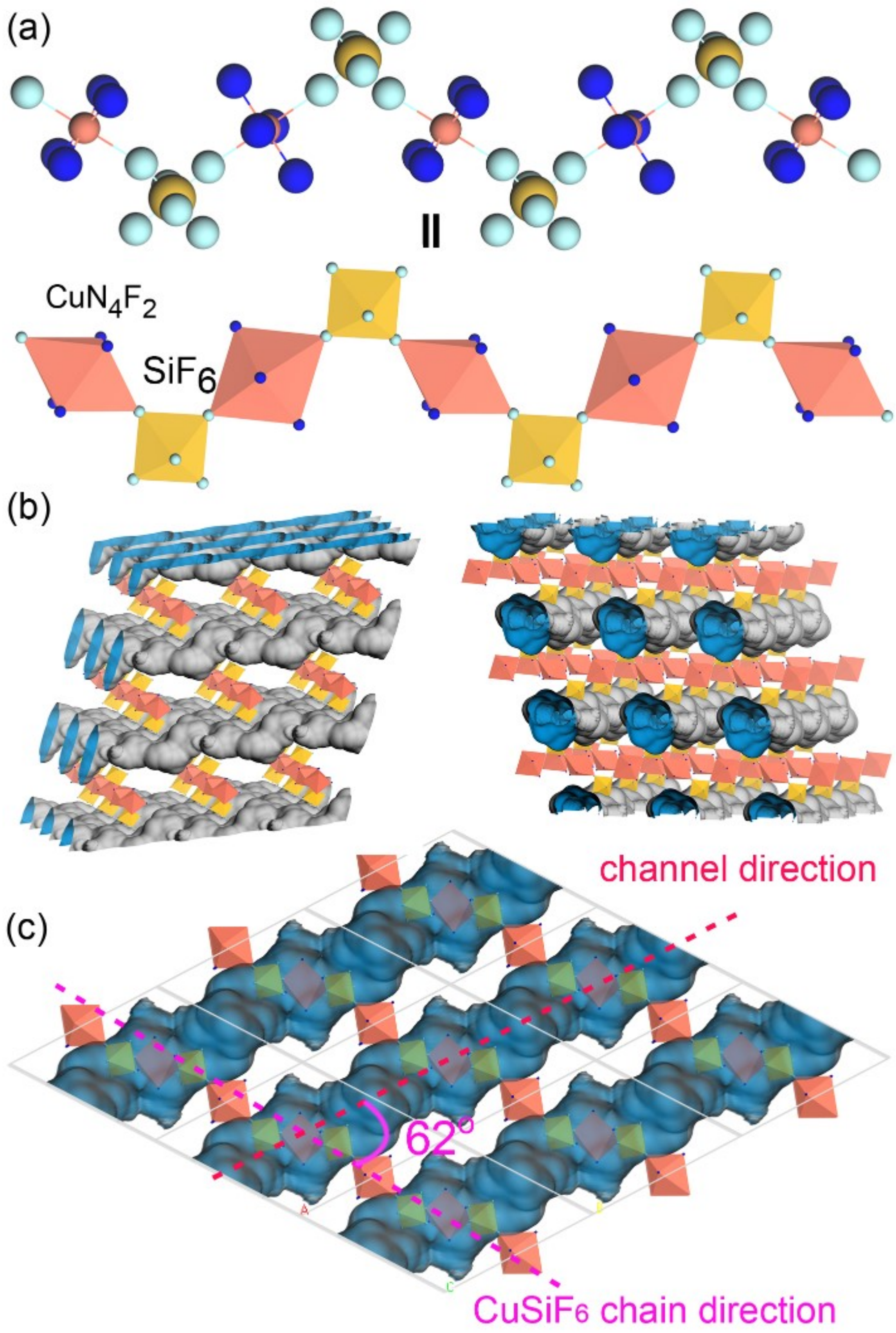

Figure S6 (a) The undulating $CuSiF_6$ chain formed by *cis*-bridging SiF_6^{2-} anions linking adjacent Cu centers. Octahedral SiF_6^{2-} and CuN_4F_2 are represented as polyhedral styles. (b,c) View of the configuration of 1D channels and the $CuSiF_6$ chains. The 1D channels are shown with Connoly surface calcualted with a probe of 1.7 Å. The 1D channel is inclined to the $CuSiF_6$ chain with an angle of ca. 62°, which is different to those found in common SIFSIX materials where the 1D channel is parallel to the Metal-SiF_6 chain.

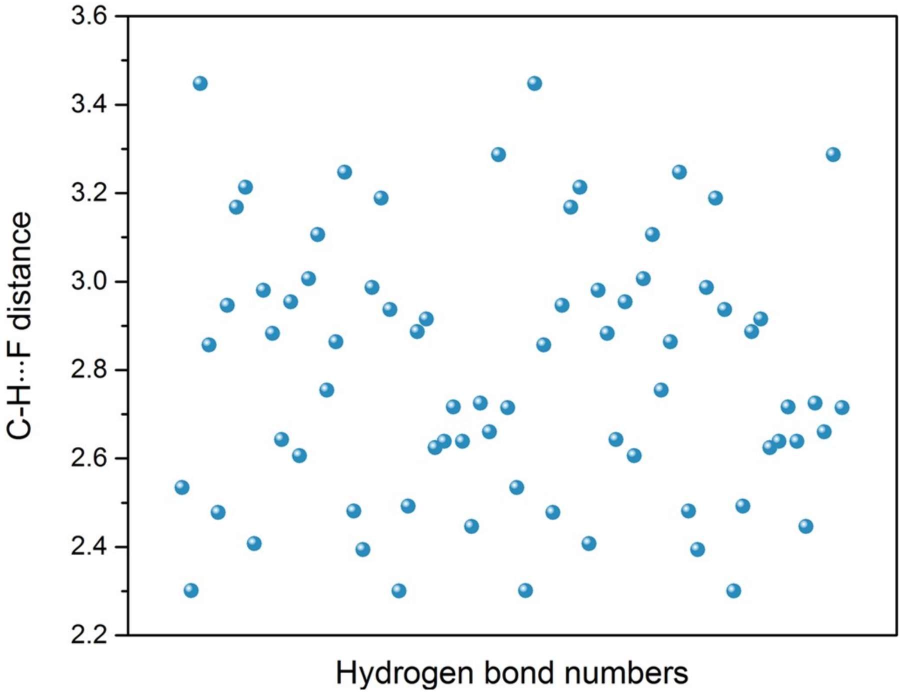

Figure S7 The statistic of C-H···F hydrogen bonds between bidmb ligands and fluorine atoms of SiF_2^- anions in SIFSIX-bidmb-Cu. As shown in the plot, the hydrogen bonds range from 2.300 Å-3.448 Å.

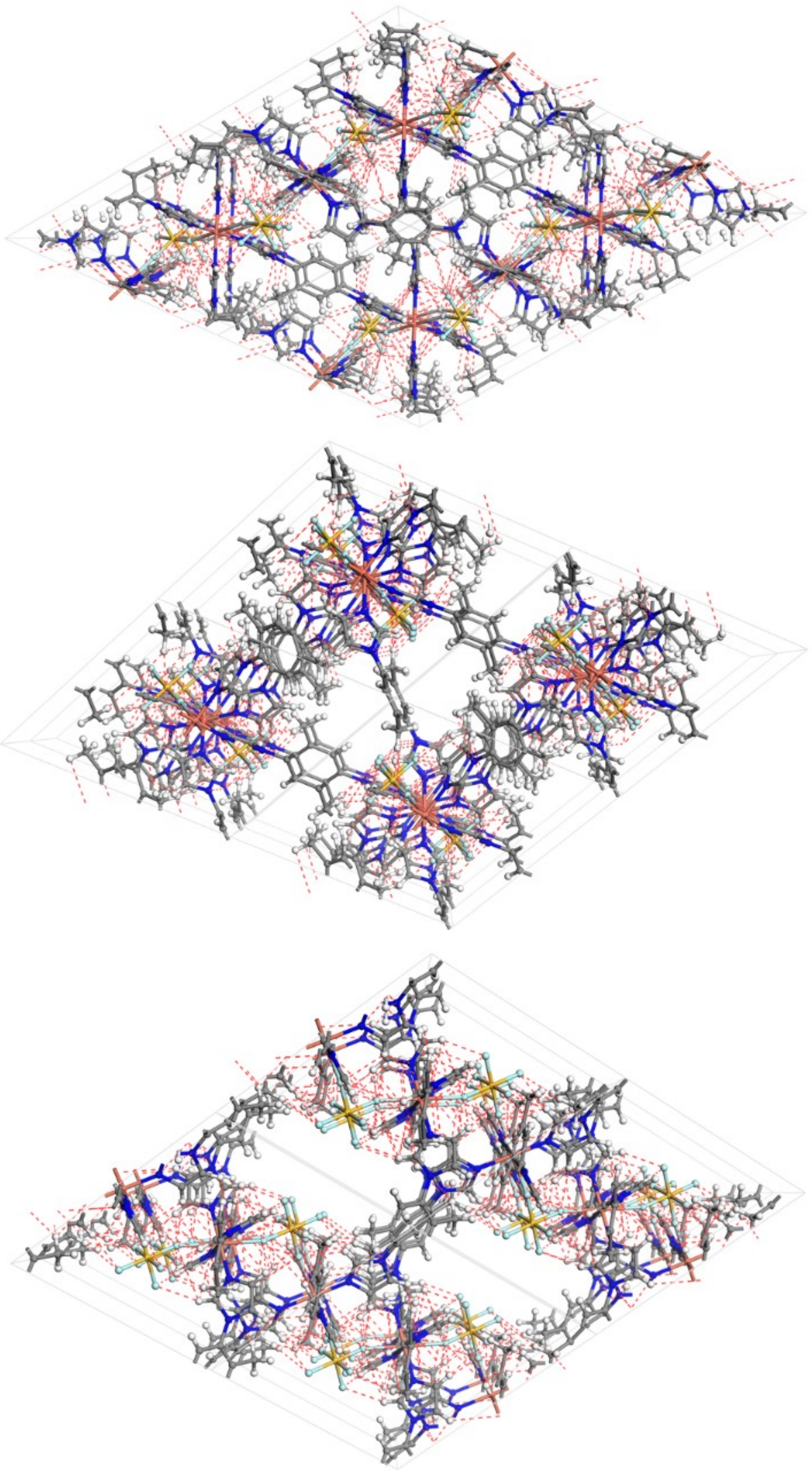

Figure S8 The ubiquitous C-H···F hydrogen bonds between bidmb ligands and fluorine atoms of SiF_2^- anions in SIFSIX-bidmb-Cu view along *a*-, *b*- and *c*-axis.

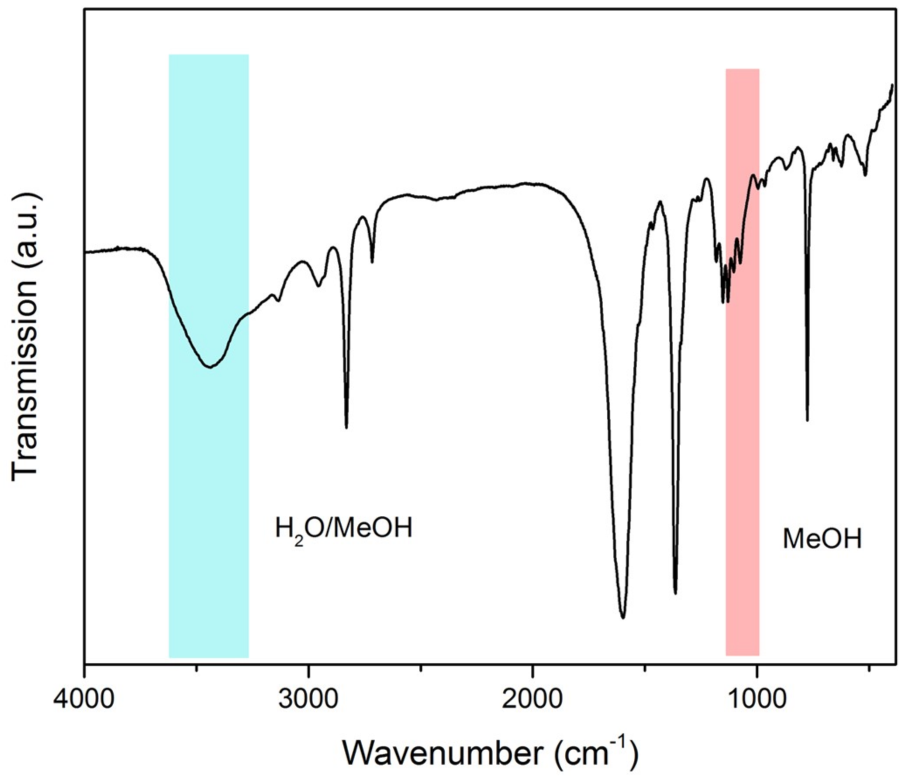

Figure S9 The IR spectrum of SIFSIX-bidmb-Cu where the signals that may indicate the existence of MeOH and H_2O were highlighted.

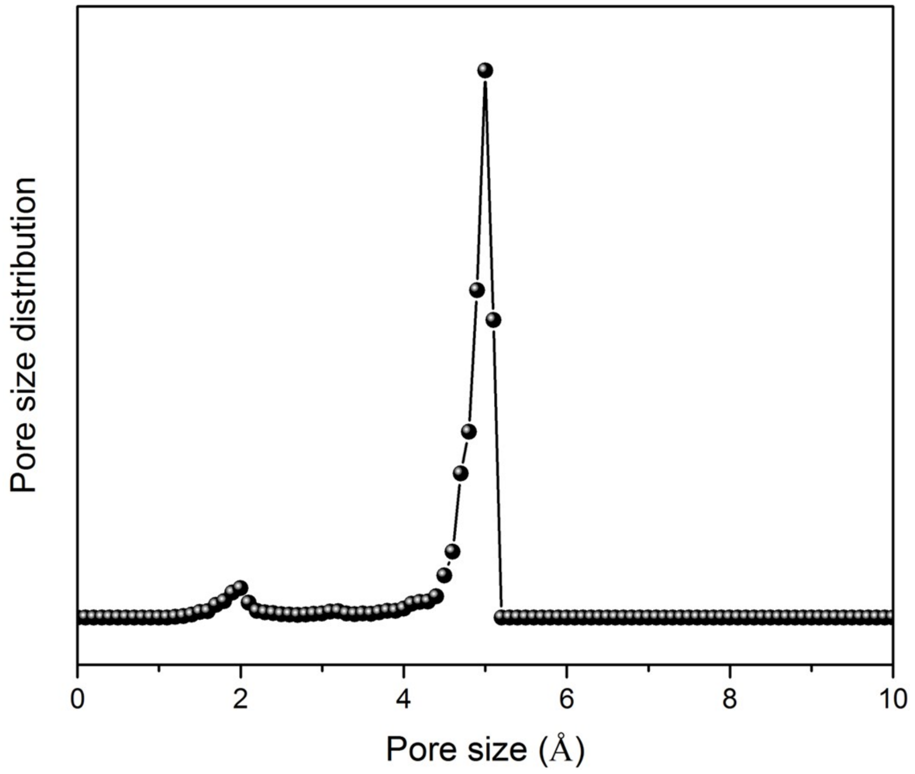

Figure S10 Pore size distribution (PSD) of SIFSIX-bidmb-Cu' which was calculated using the well-known method by Gubbins et al.[17] The van der Waals diameters of the framework atoms were adopted from the Cambridge Crystallographic Center.

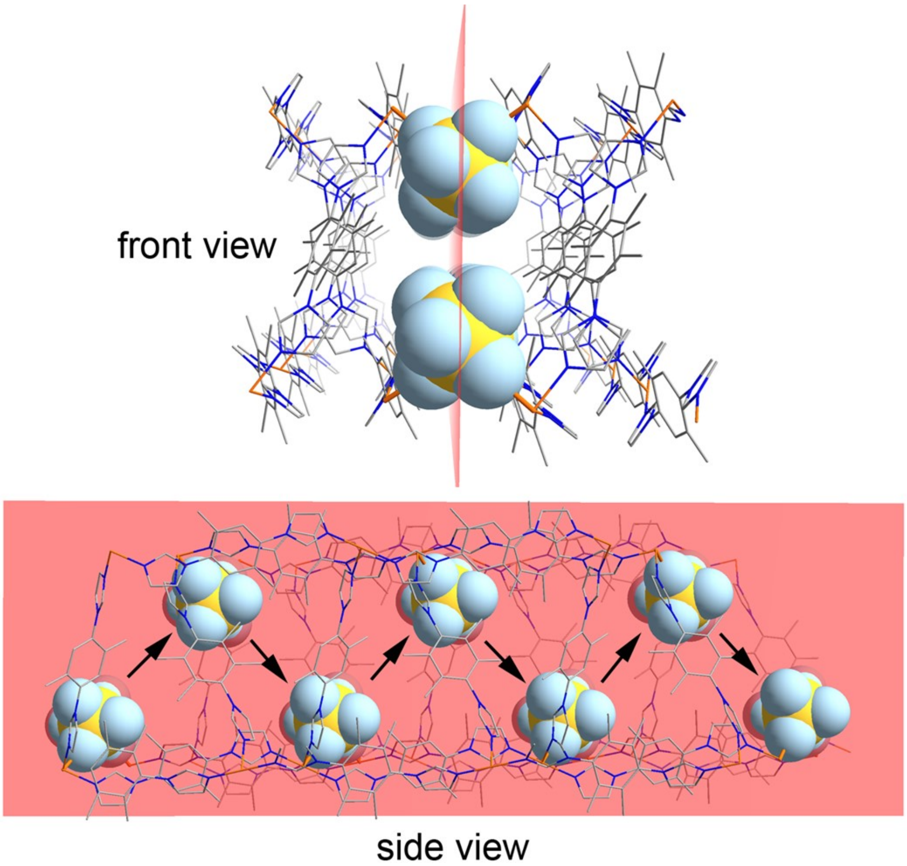

Figure S11 The coplanar and zig-zag SiF_6^{2-} anions distribution along the channel direction. The light red plane is defined by the Si centers of SiF_6^{2-} anions.

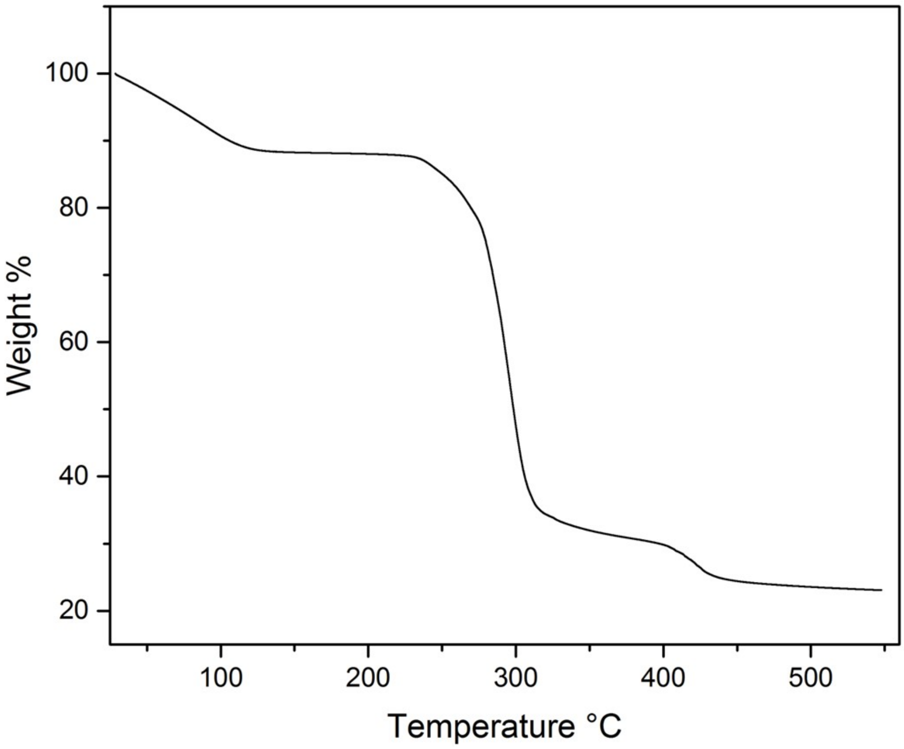

Figure S12 The TGA curve of SIFSIX-bidmb-Cu.

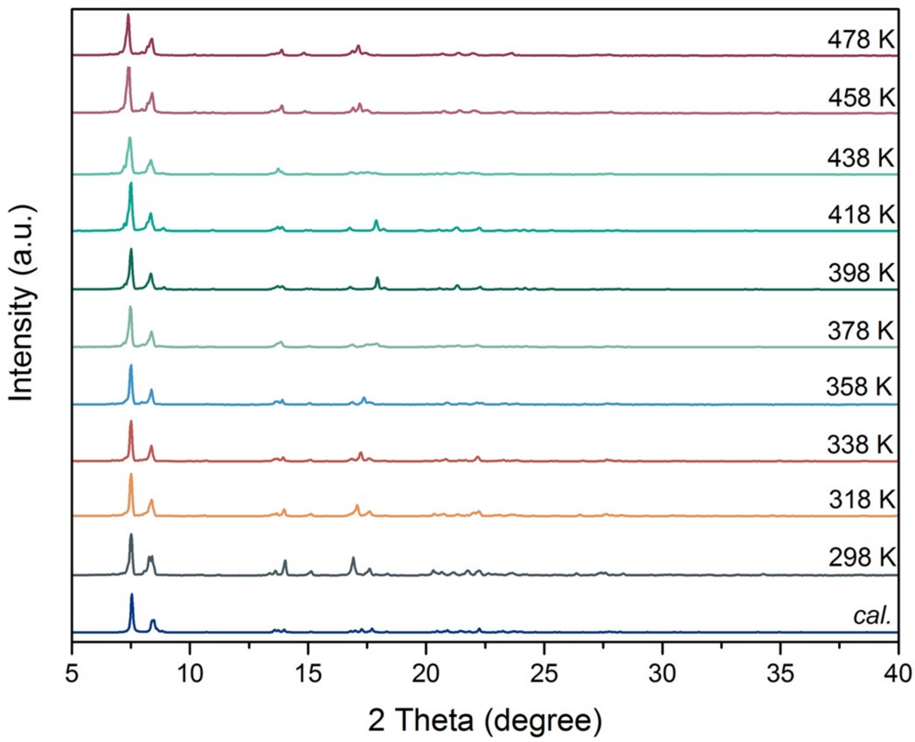

Figure S13 The variable temperature PXRD patterns measured for freshly synthesized samples of SIFSIX-bidmb-Cu.

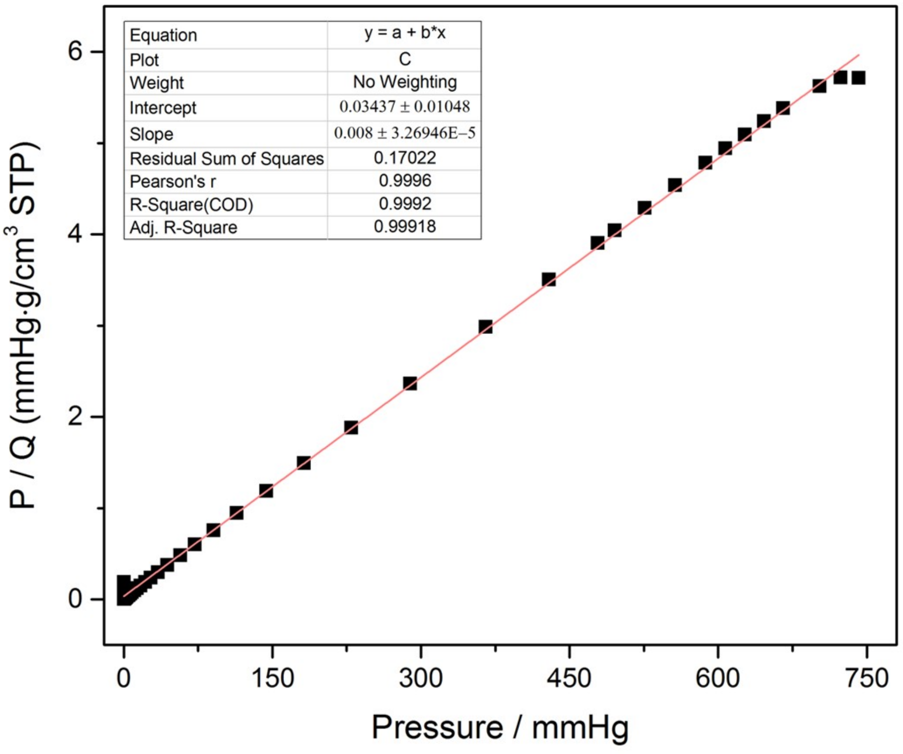

Figure S14 The Langmuir fitting of 77 K N_2 adsorption isotherms.

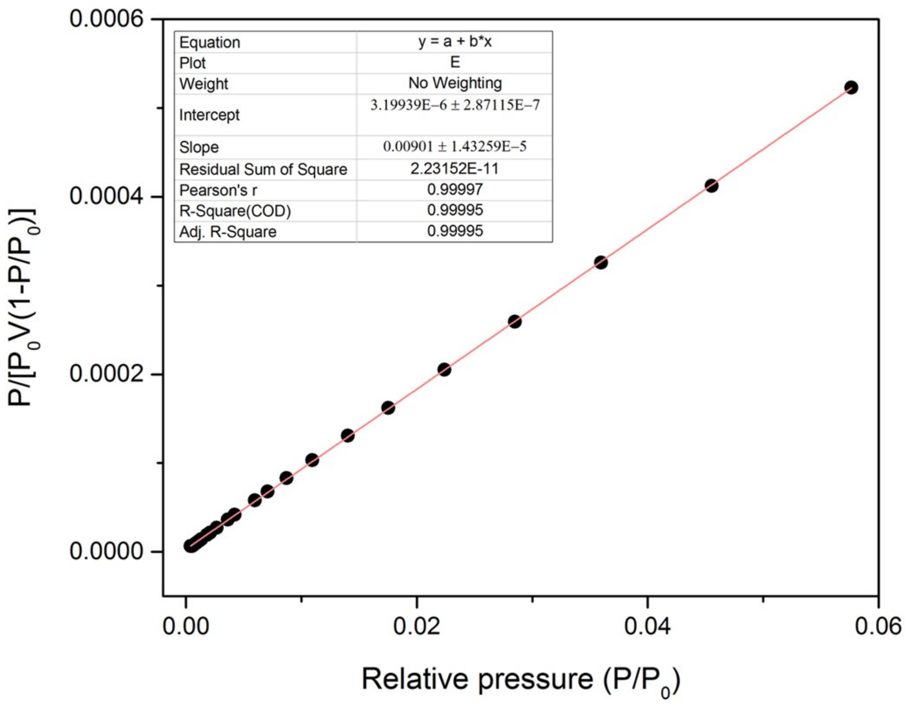

Figure S15 The Brunauer-Emmett-Teller (BET) fitting of the 77 K N_2 adsorption isotherm (0.01 < P/P_0 < 0.06).

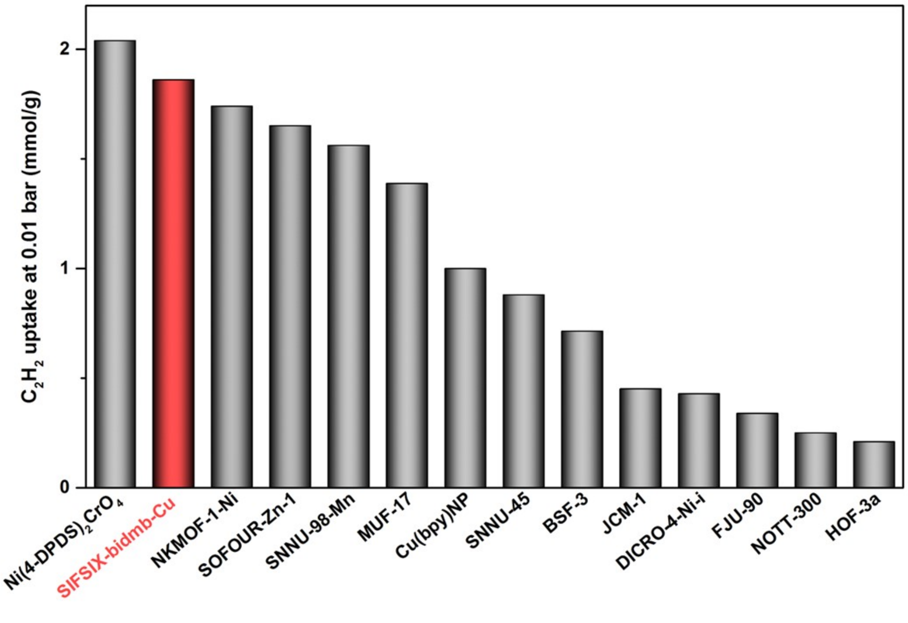

Figure S16 Comparison of C_2H_2 uptakes at 0.01 bar for various leading physisorbents (Non SIFSIX materials).

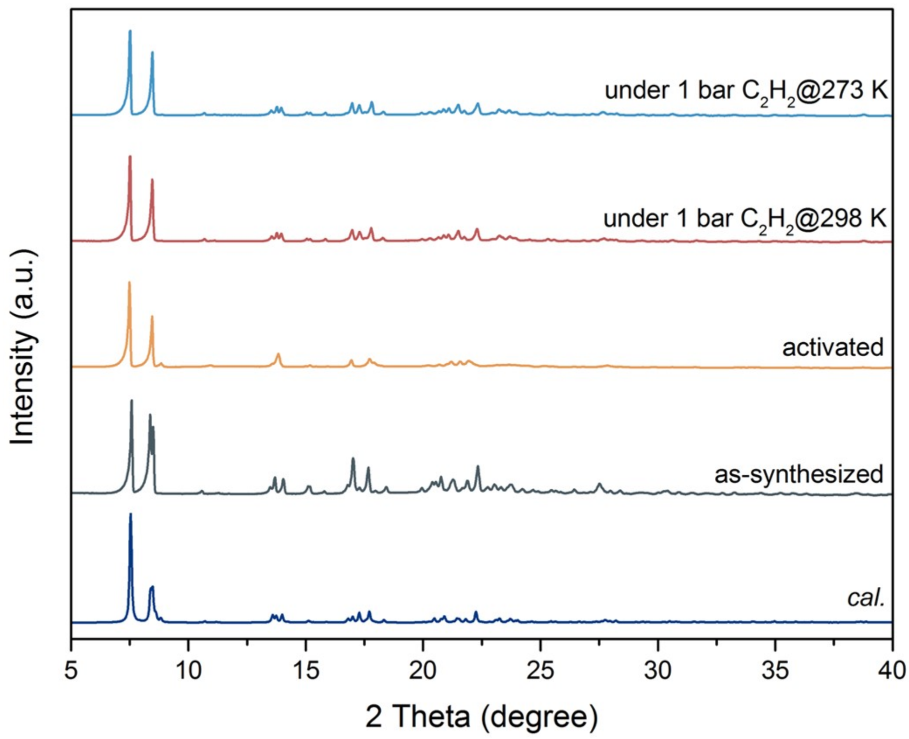

Figure S17 Comparison of PXRD patterns calculated from crystal strucutres of SIFSIX-bidmb-Cu and SIFSIX-bidmb-Cu' with those measured for as-synthesized sample and sample under 1 bar C_2H_2 at 273 K and 298 K.

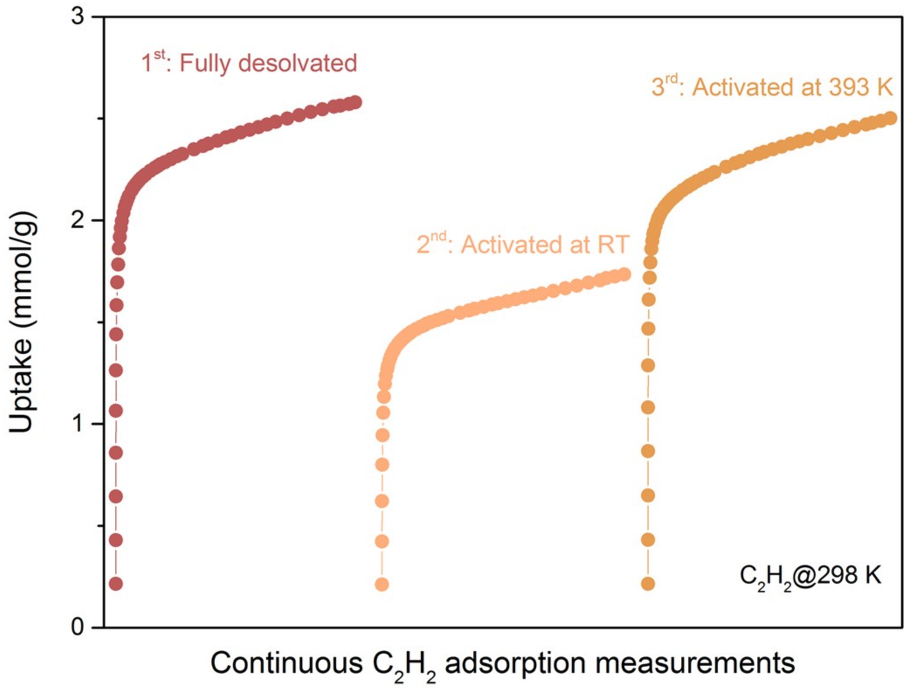

Figure S18 Consecutive measurements of C_2H_2 adsorption at 298 K with different activation conditions.

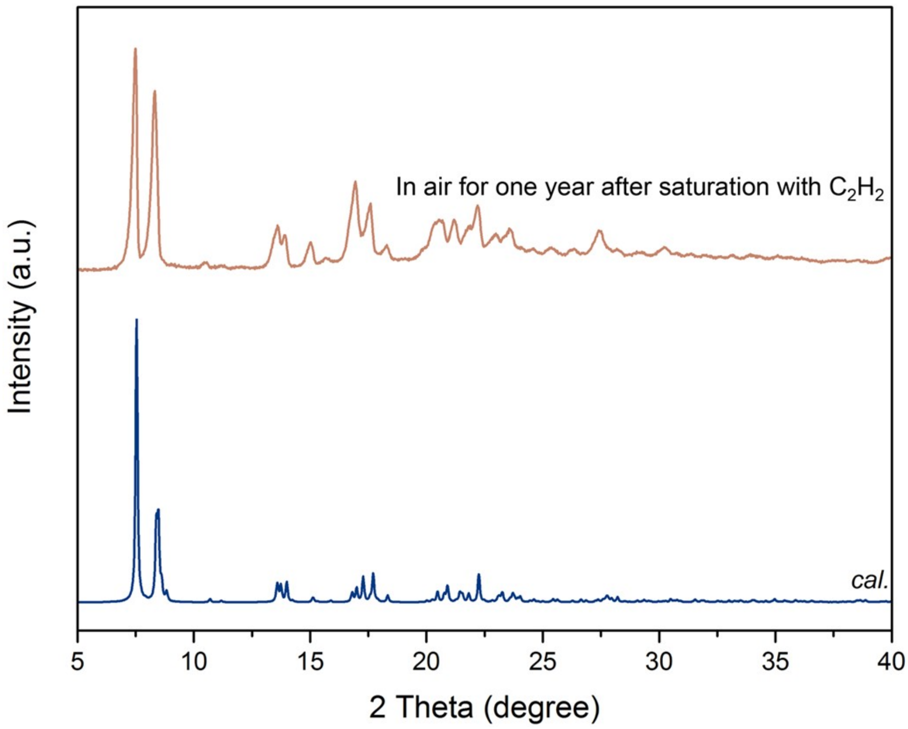

Figure S19 Comparison of PXRD pattern calculated from the crystal structure of SIFSIX-bidmb-Cu with that of sample placed in air for one year after saturation with C_2H_2.

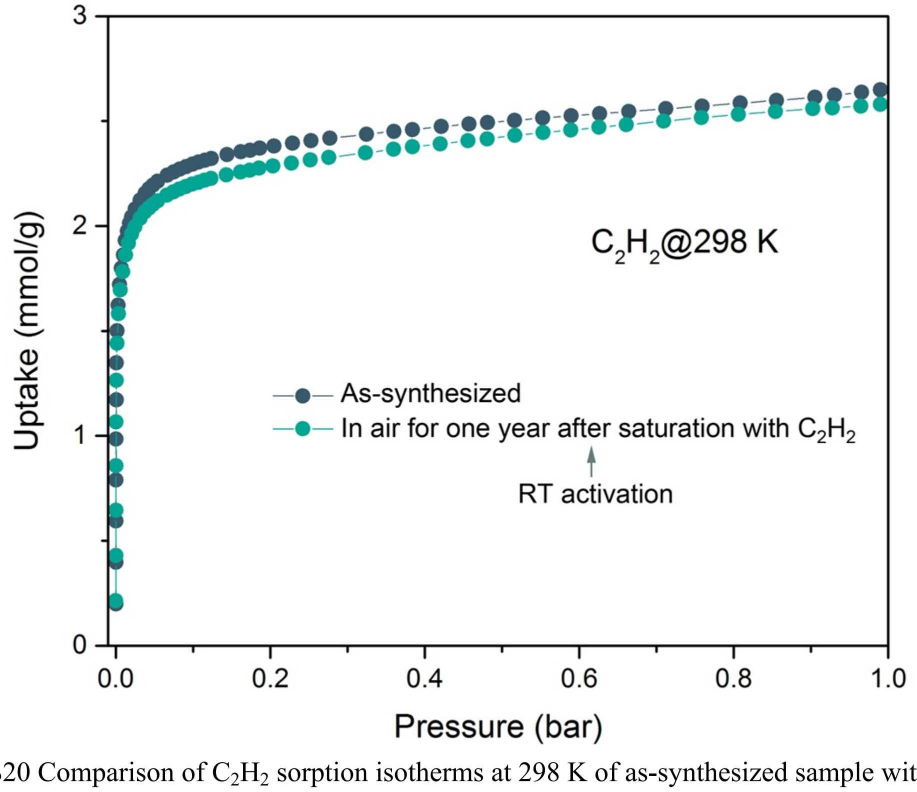

Figure S20 Comparison of C_2H_2 sorption isotherms at 298 K of as-synthesized sample with that of sample placed in air for one year after saturation of C_2H_2. Notably, the reactivation of sample was conducted at room temperature under high vacuum.

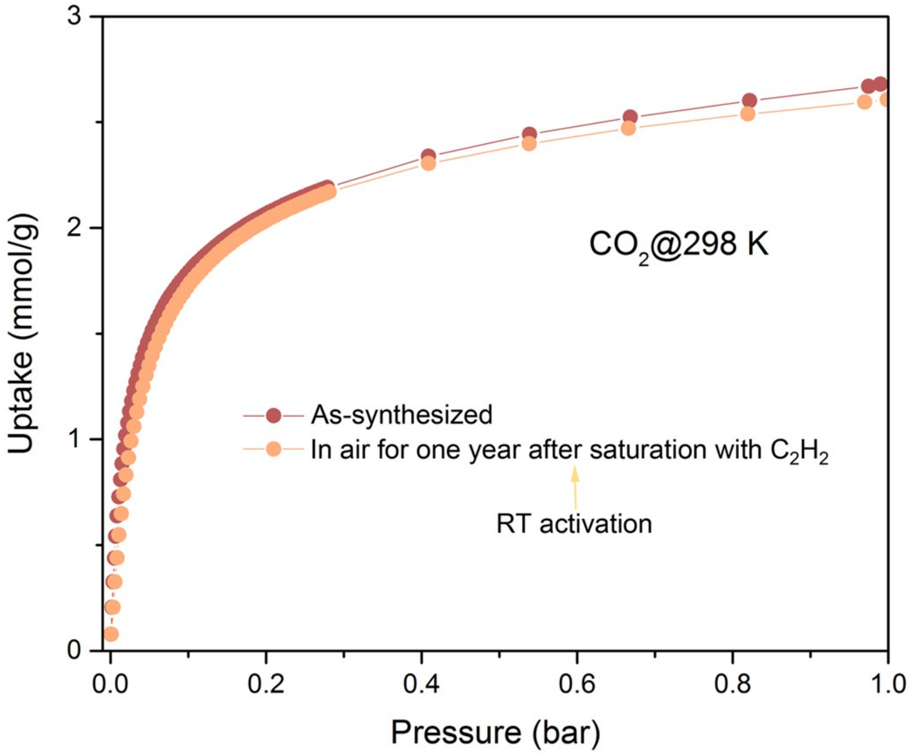

Figure S21 Comparison of CO_2 sorption isotherms at 298 K of as-synthesized sample with that of sample placed in air for one year after saturation of C_2H_2. Notably, the reactivation of sample was conducted at room temperature under high vacuum.

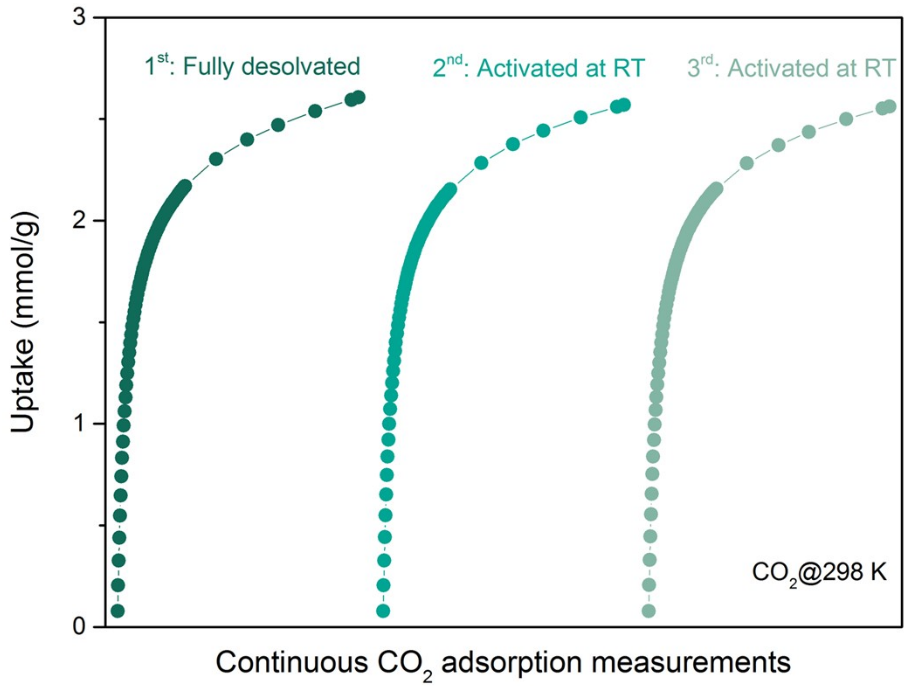

Figure S22 Consecutive measurements of CO_2 adsorption at 298 K.

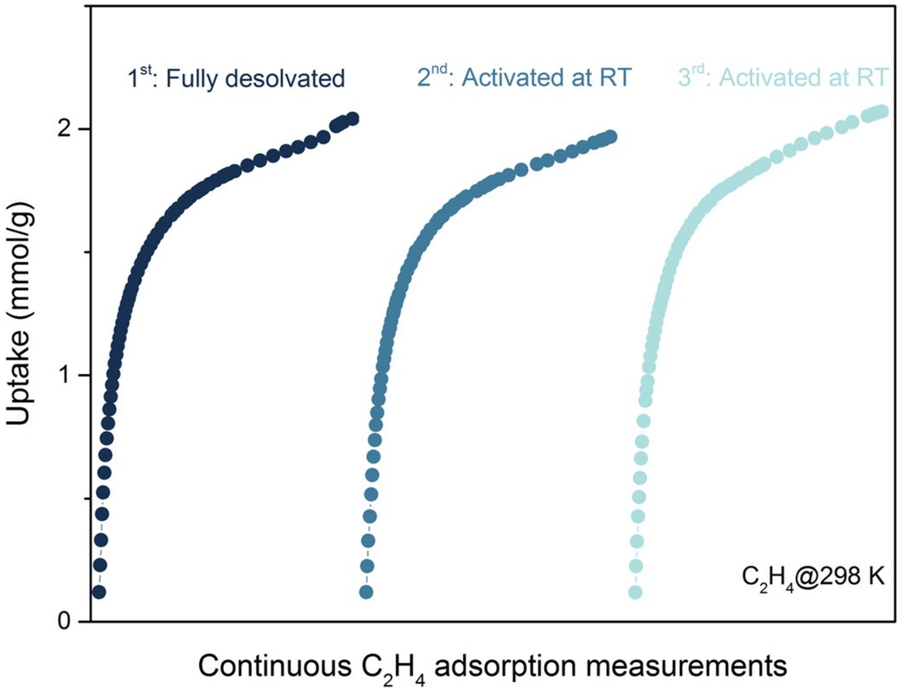

Figure S23 Consecutive measurements of C_2H_4 adsorption at 298 K.

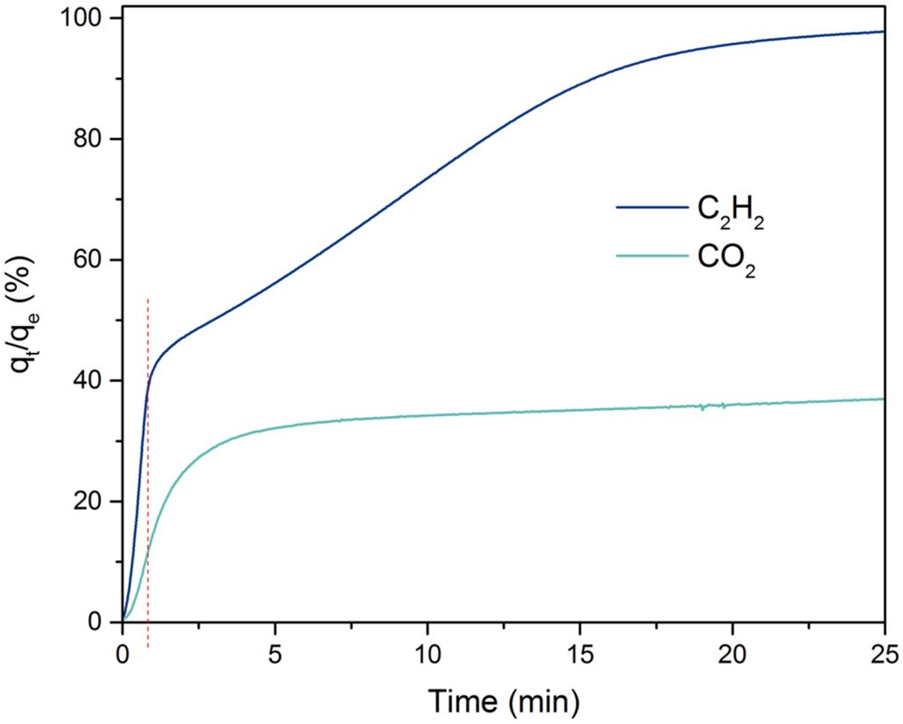

Figure S24 The time-dependent adsorption kinetics profiles. q_e = the saturated sorption uptake, q_t = the uptake at different time.

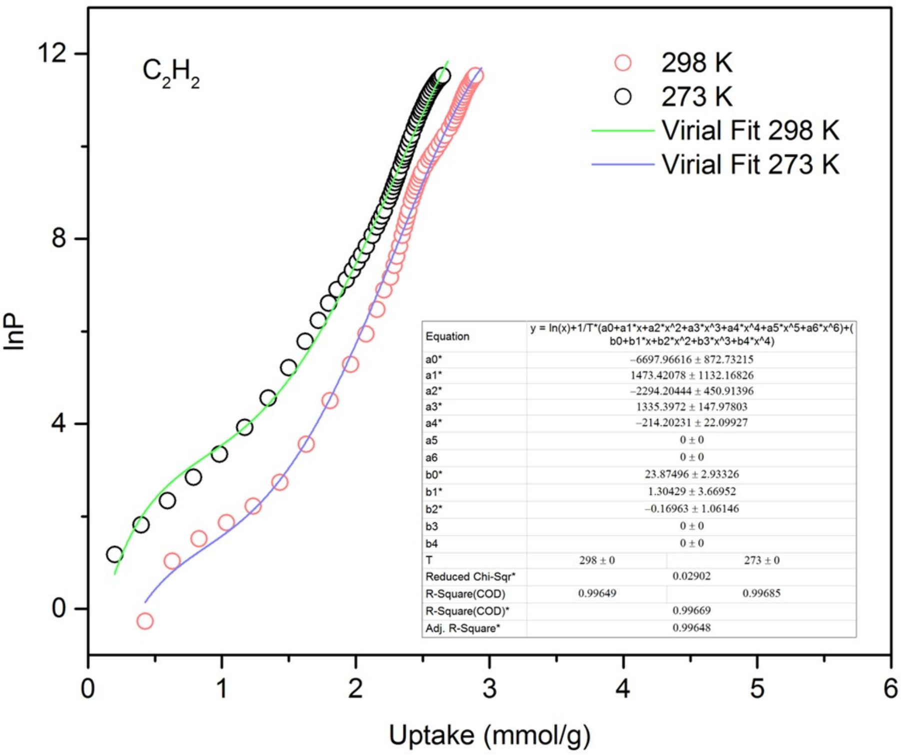

Figure S25 The Virial fitting of C_2H_2 adsorption isotherms collected at 273 and 298 K.

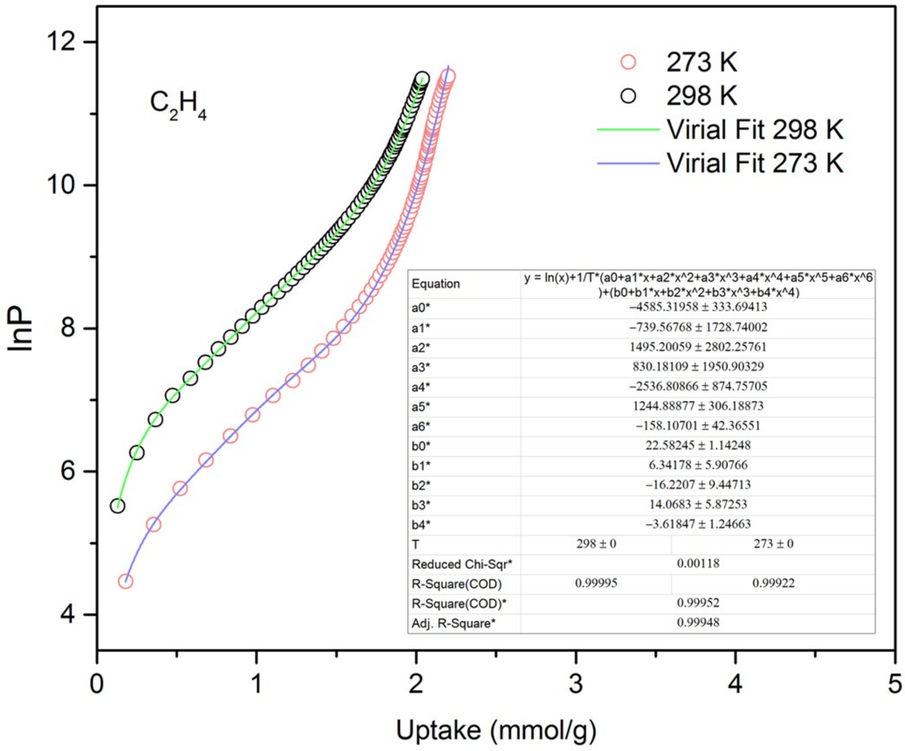

Figure S26 The Virial fitting of C_2H_4 adsorption isotherms collected at 273 and 298 K.

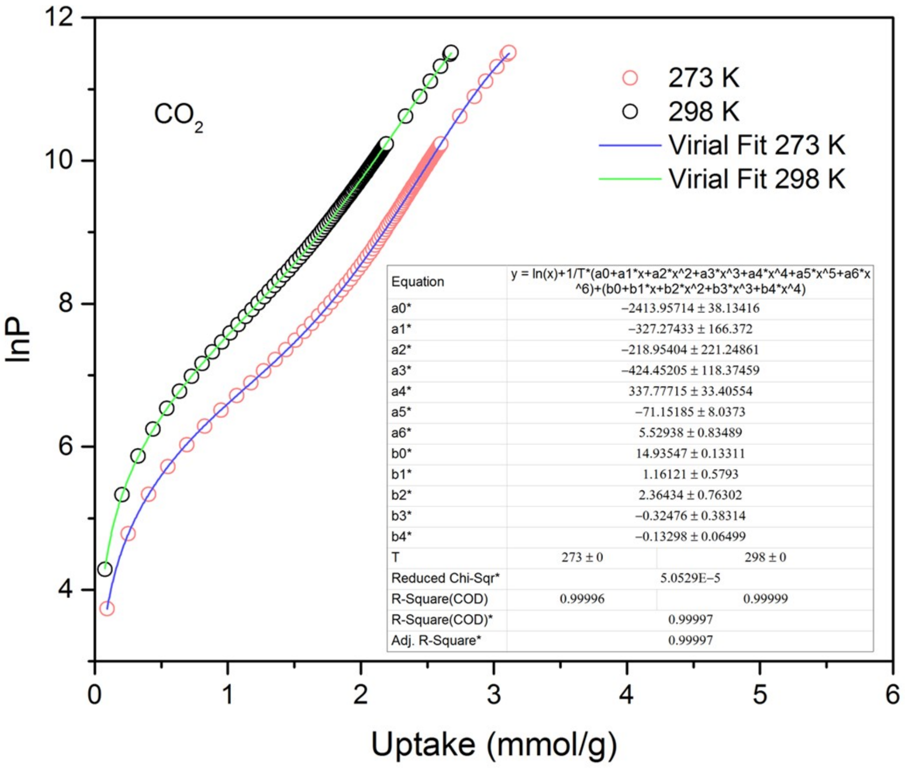

Figure S27 The Virial fitting of CO_2 adsorption isotherms collected at 273 and 298 K.

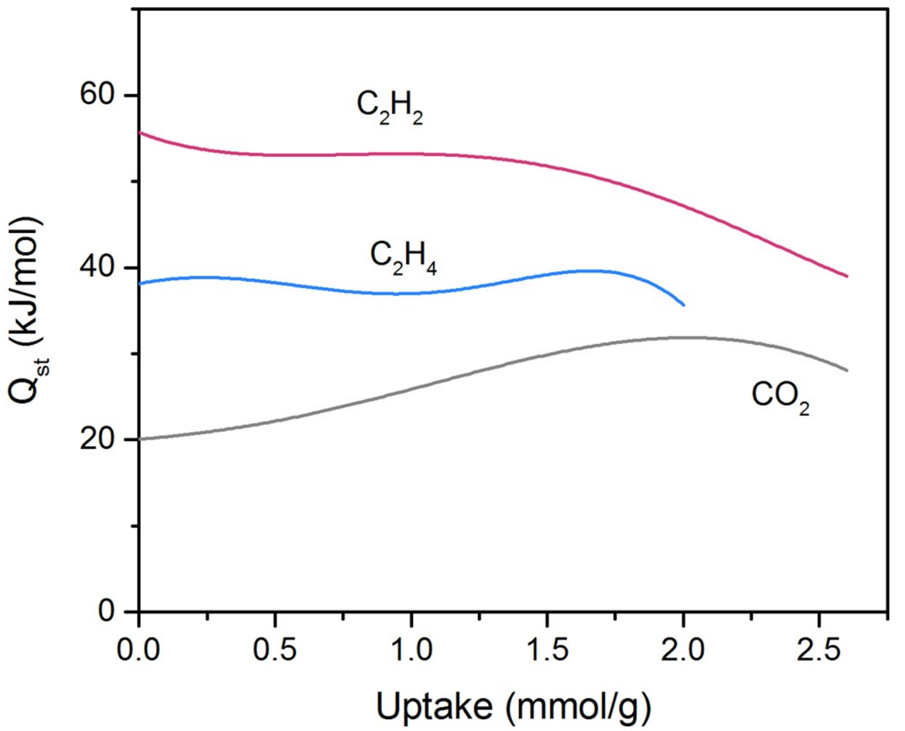

Figure S28 The calculated heat of adsorption (Q_{st}) of C_2H_2, C_2H_4 and CO_2 for SIFSIX-bidmb-Cu.

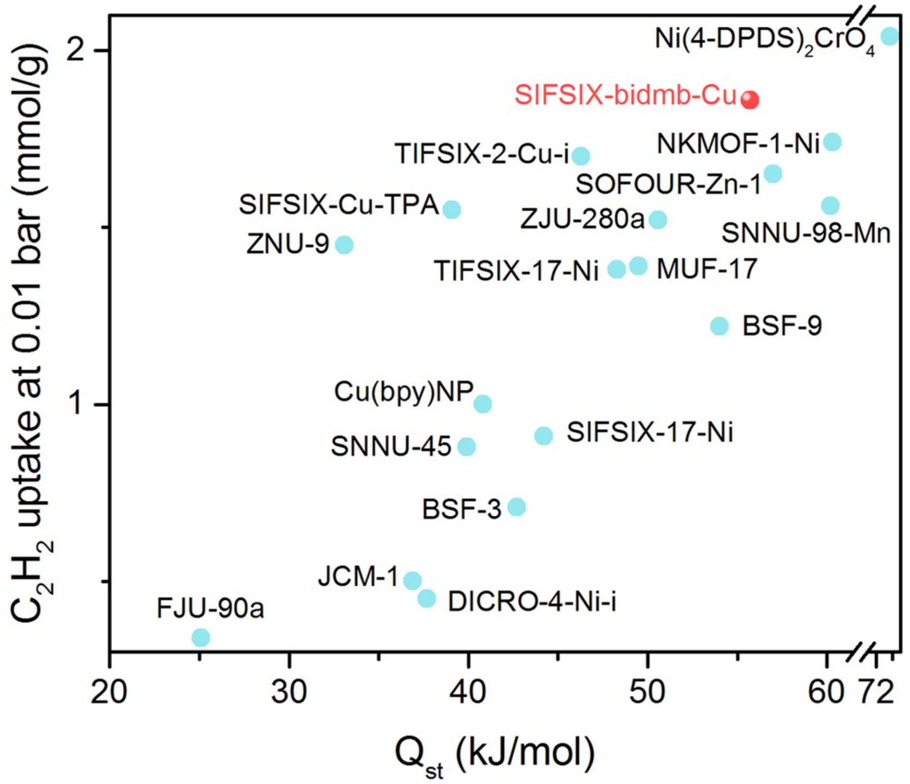

Figure S29 Comparison of C_2H_2 uptakes at 0.01 bar and the heat of adsorption (Q_{st}) of C_2H_2 for various top-performing physisorbents.

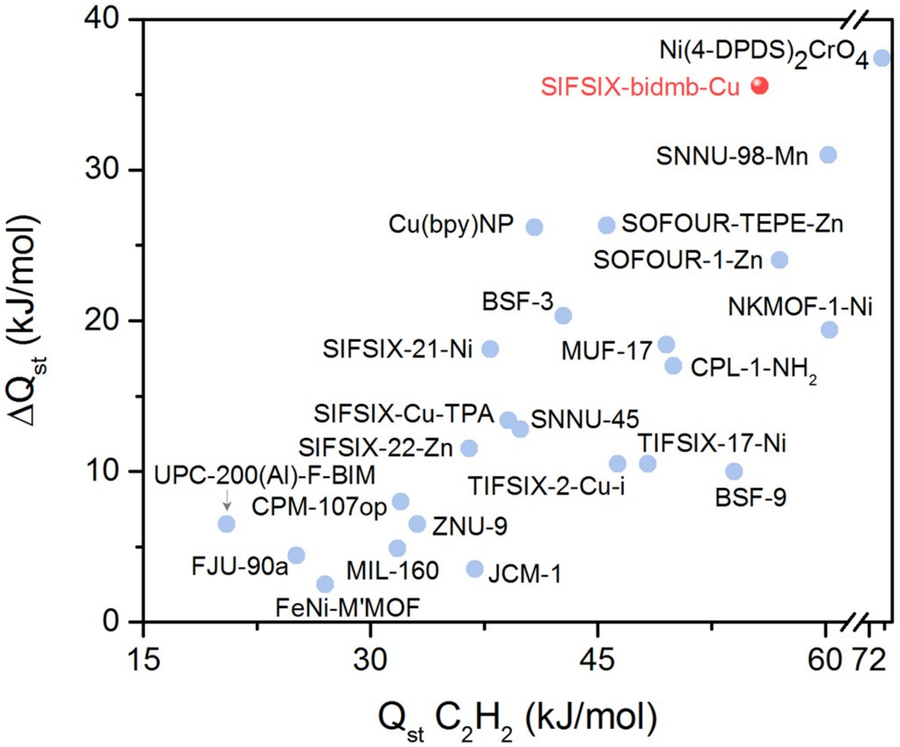

Figure S30 Comparison of the heat of adsorption (Q_{st}) of C_2H_2 and the difference in Q_{st} values between C_2H_2 and CO_2 for various top-performing physisorbents.

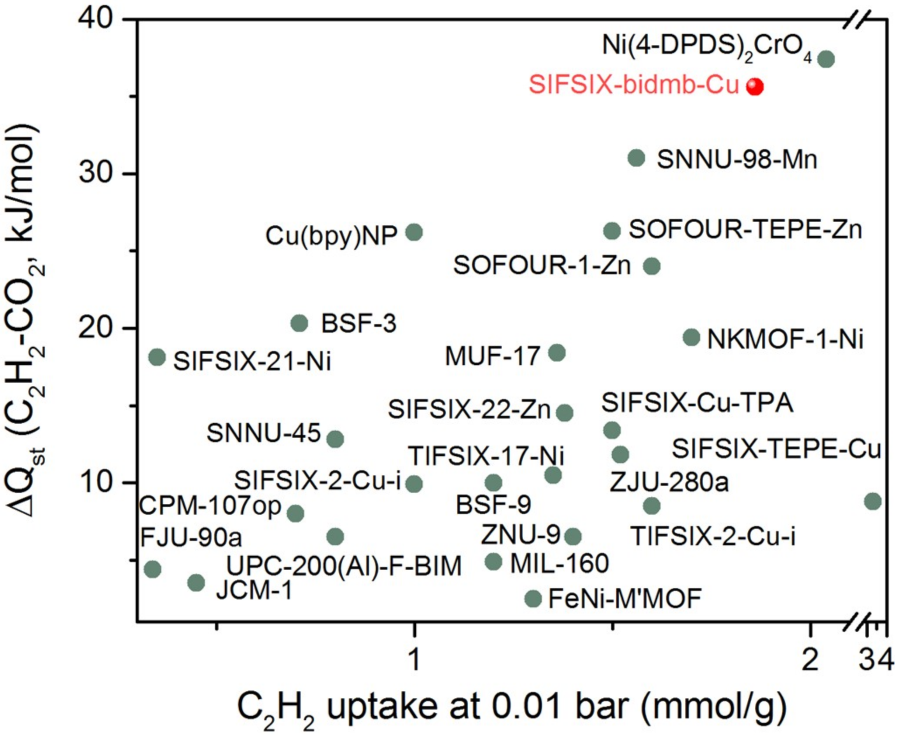

Figure S31 Comparison of the uptake of C_2H_2 at 0.01 bar and the difference in Q_{st} values between C_2H_2 and CO_2 for various top-performing physisorbents.

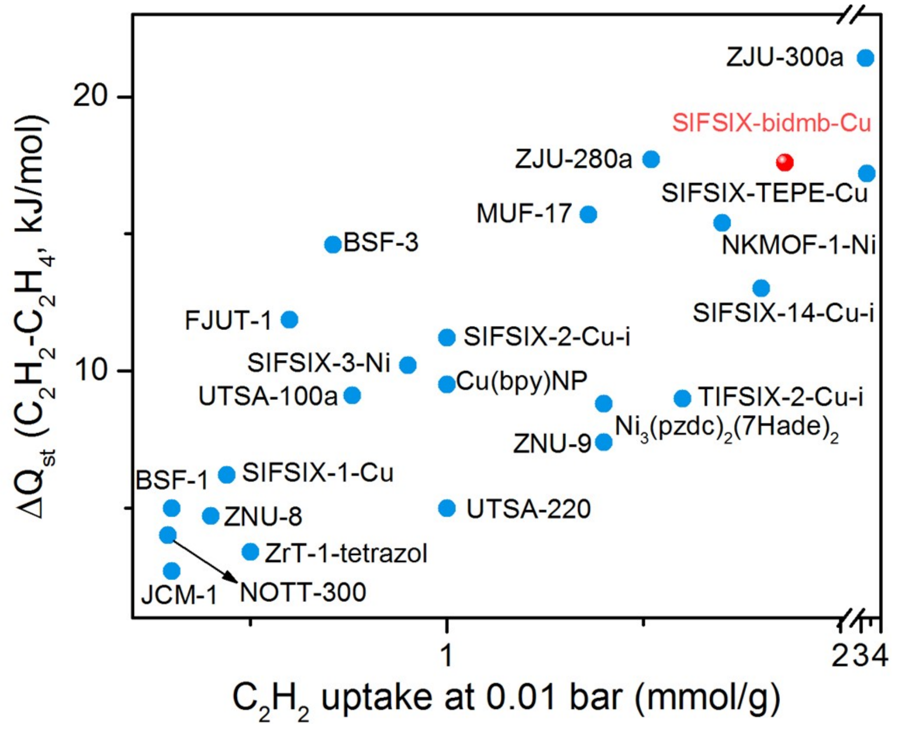

Figure S32 Comparison of the uptake of C_2H_2 at 0.01 bar and the difference in Q_{st} values between C_2H_2 and C_2H_4 for various top-performing physisorbents.

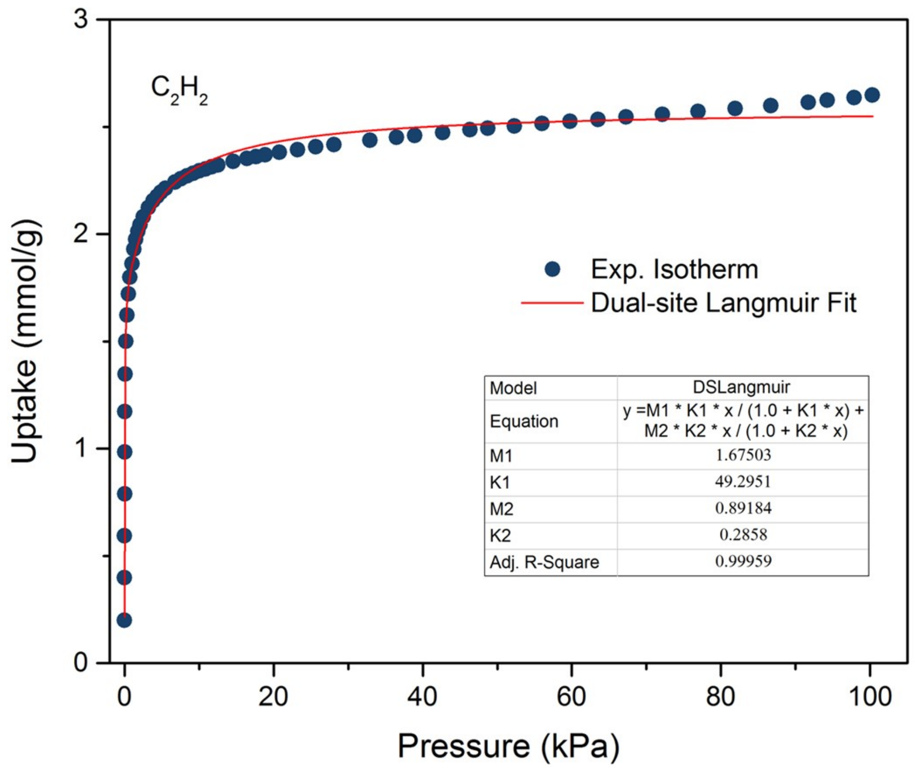

Figure S33 Dual-site Langmuir fitting of C_2H_2 adsorption isotherm at 298 K.

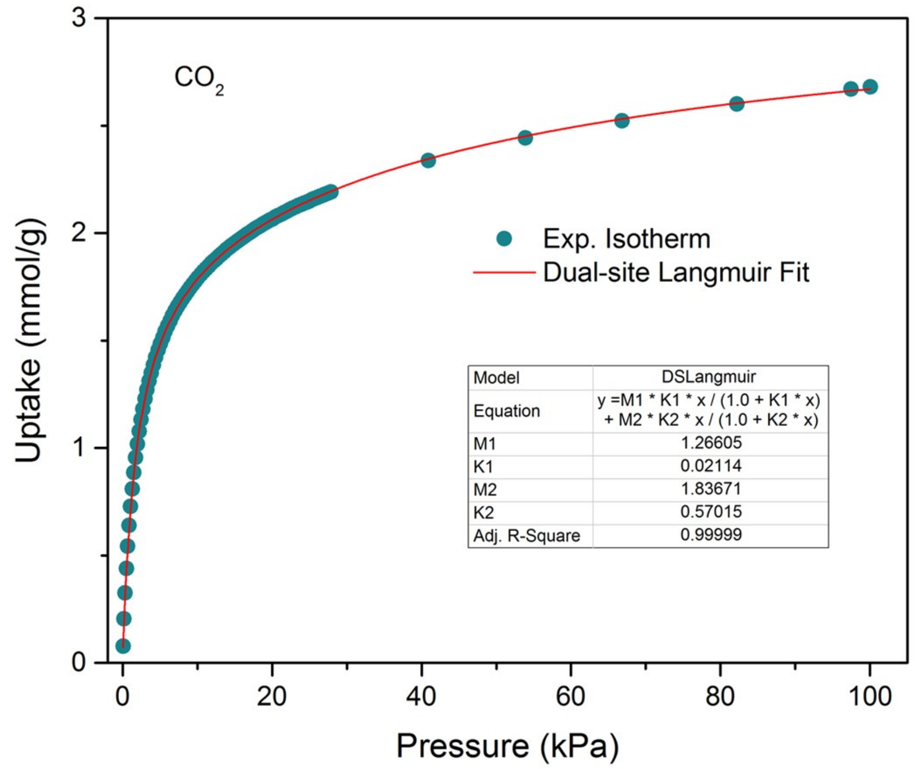

Figure S34 Dual-site Langmuir fitting of CO_2 adsorption isotherm at 298 K.

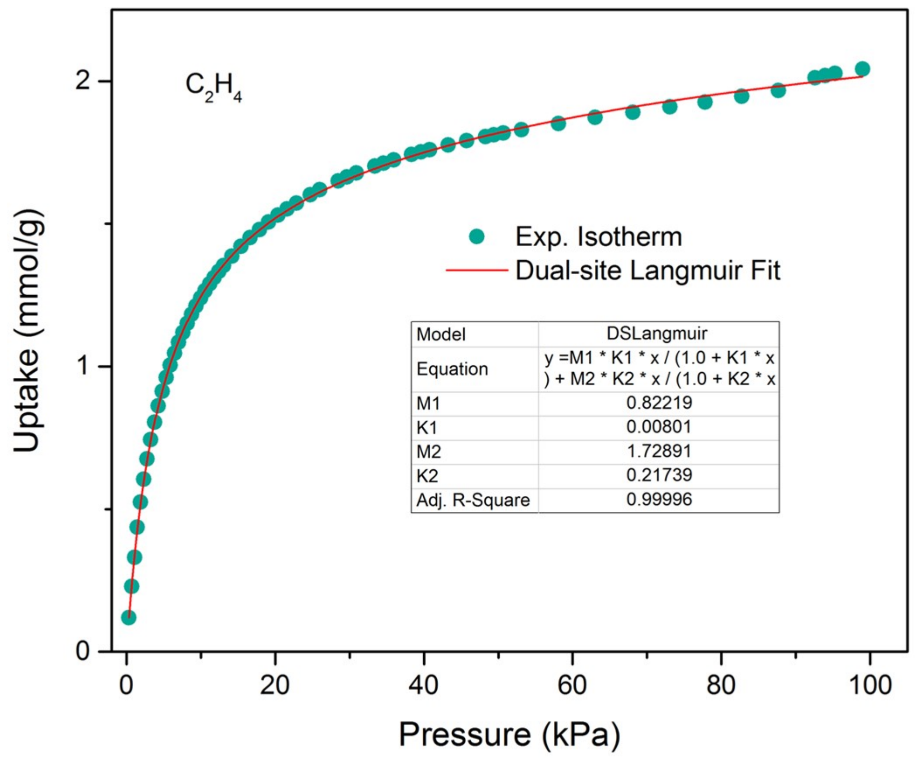

Figure S35 Dual-site Langmuir fitting of C_2H_4 adsorption isotherm at 298 K.

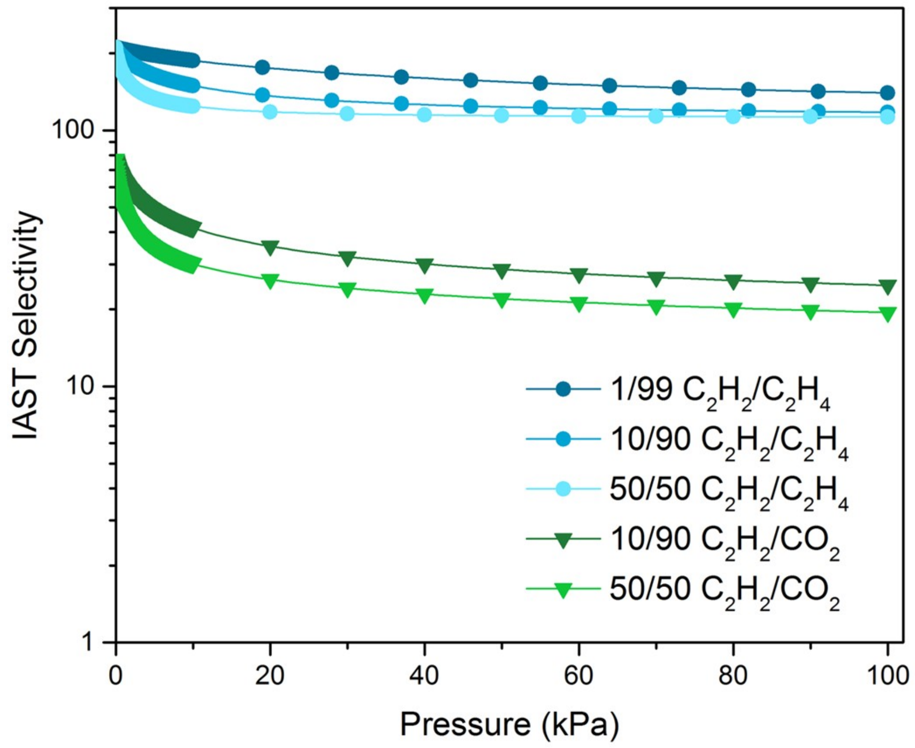

Figure S36 The IAST selectivities of C_2H_2/CO_2 and C_2H_2/C_2H_4 mixtures with different compositions.

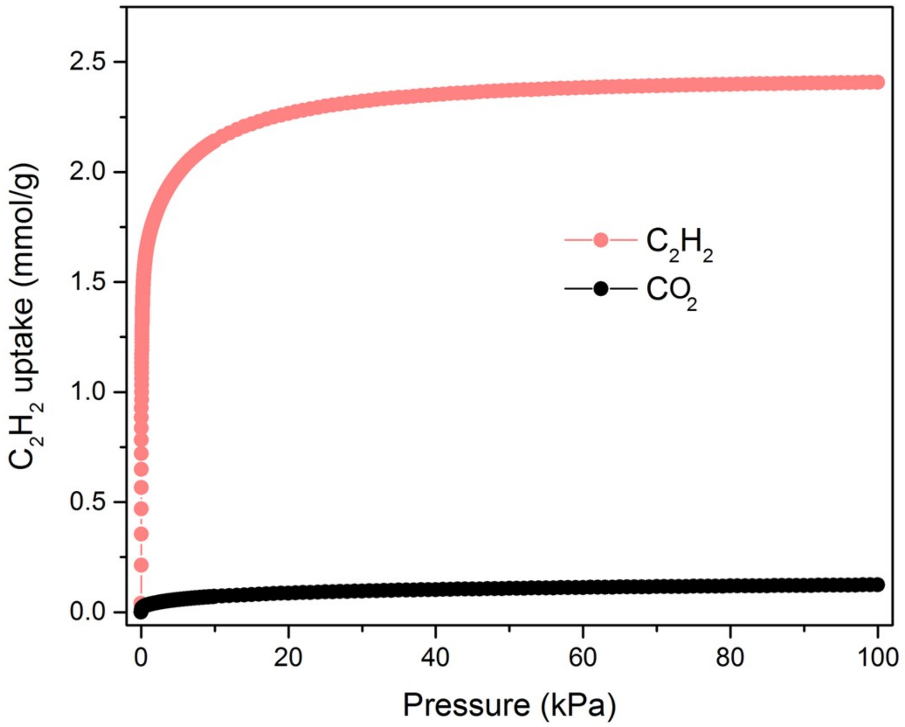

Figure S37 Co-adsorption isotherms for 50/50 binary C_2H_2/CO_2 mixtures predicted via IAST.

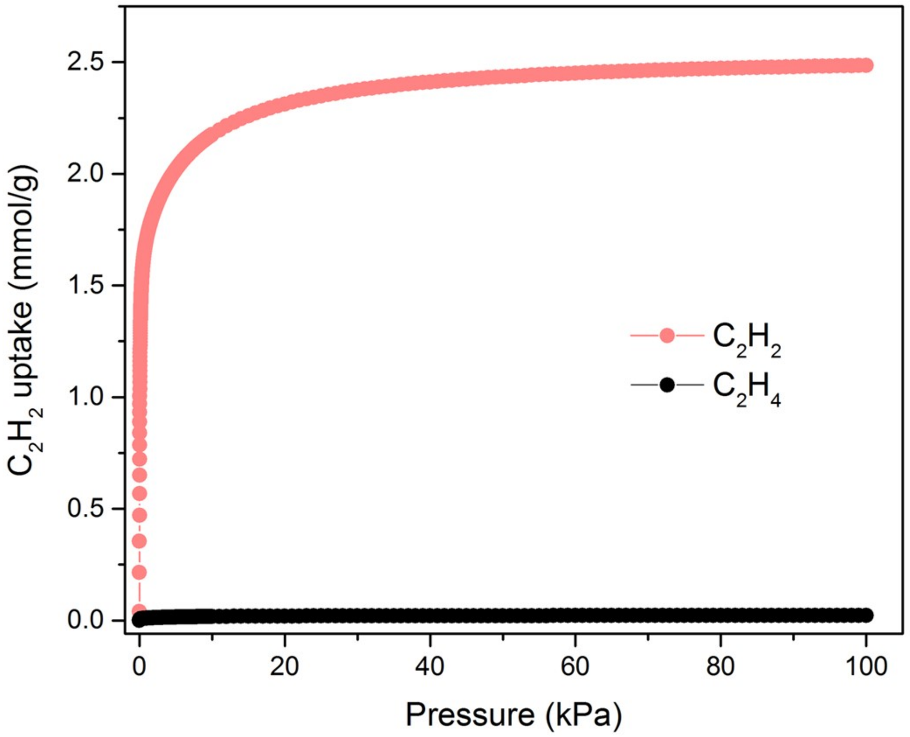

Figure S38 Co-adsorption isotherms for 50/50 binary C_2H_2/C_2H_4 mixtures predicted via IAST.

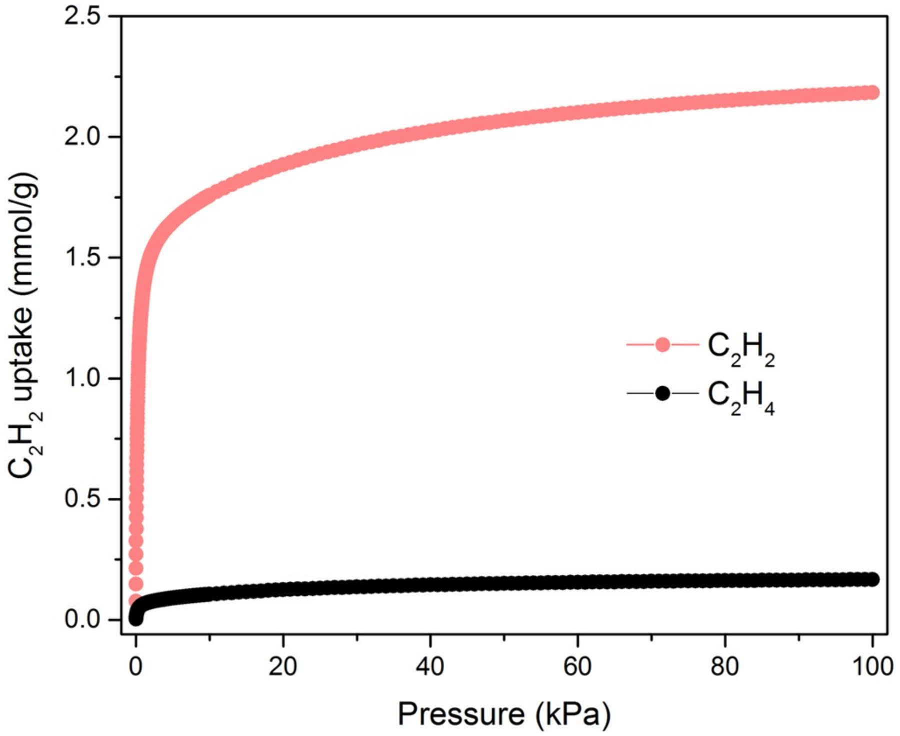

Figure S39 Co-adsorption isotherms for 10/90 binary C_2H_2/C_2H_4 mixtures predicted via IAST.

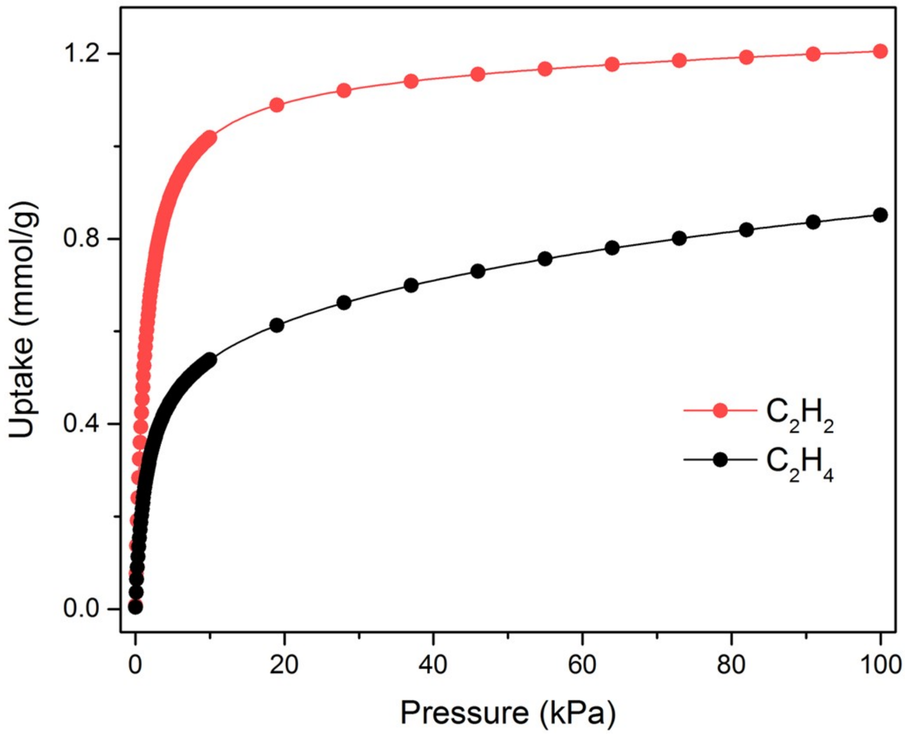

Figure S40 Co-adsorption isotherms for 1/99 binary C_2H_2/C_2H_4 mixtures predicted via IAST.

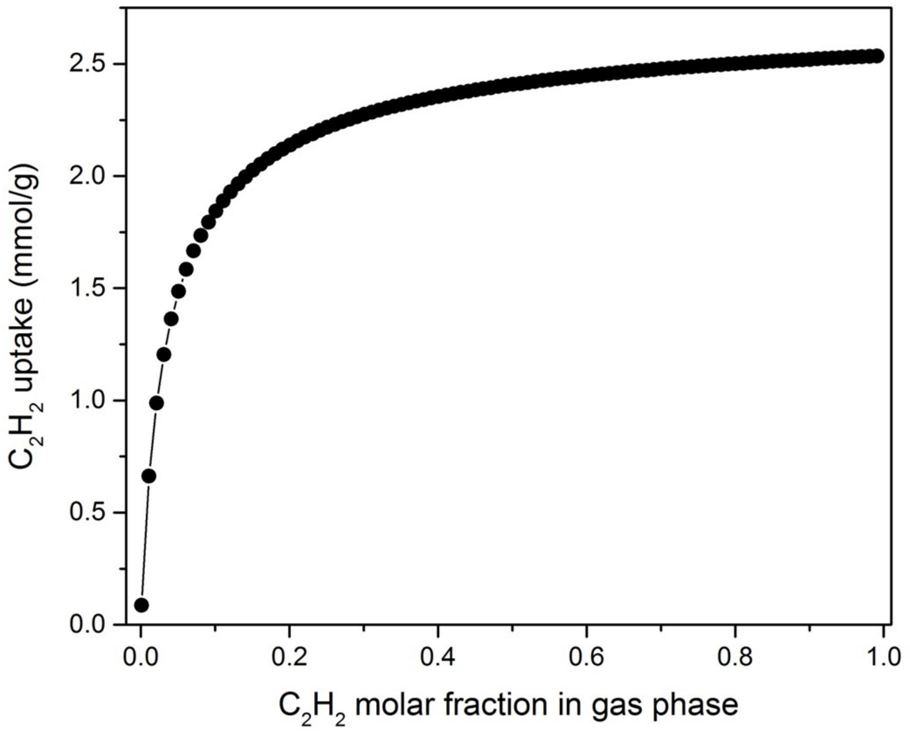

Figure S41 The C_2H_2 uptake from binary C_2H_2/CO_2 mixtures with different C_2H_2 molar fraction predicted via IAST.

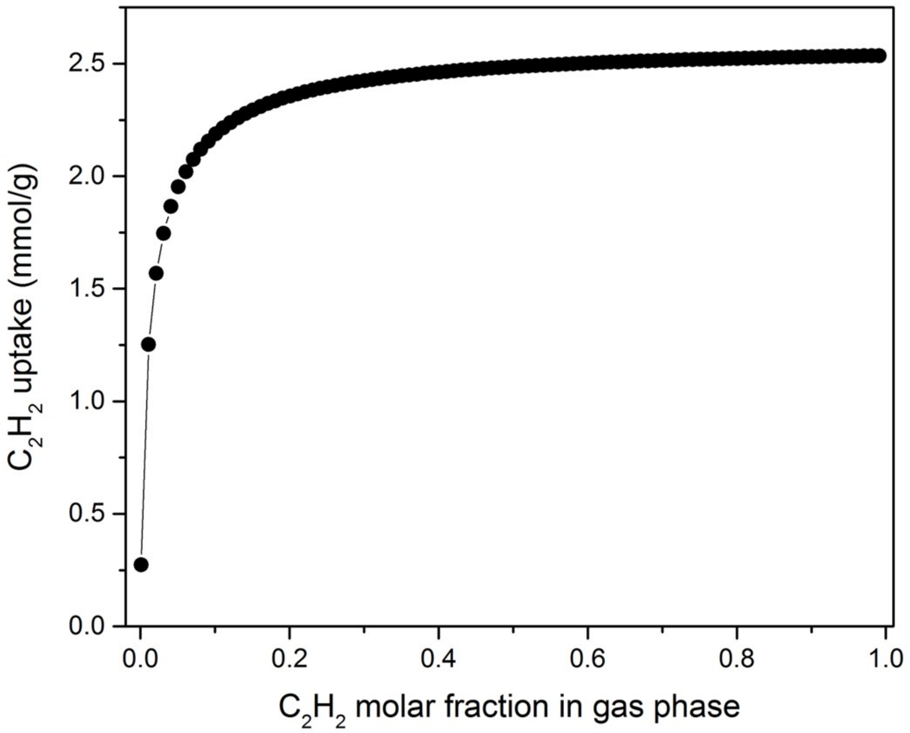

Figure S42 The C_2H_2 uptake from binary C_2H_2/C_2H_4 mixtures with different C_2H_2 molar fraction predicted via IAST.

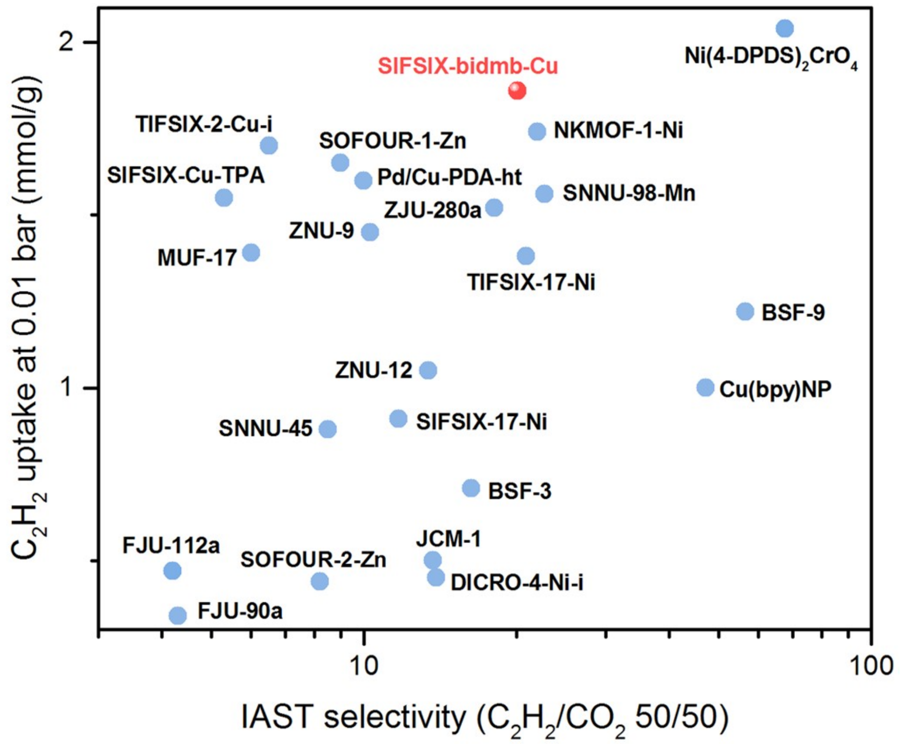

Figure S43 Comparison of the C_2H_2 uptakes at 0.01 bar with IAST selectivities of 50/50 C_2H_2/CO_2 mixture for various top-performing physisorbents.

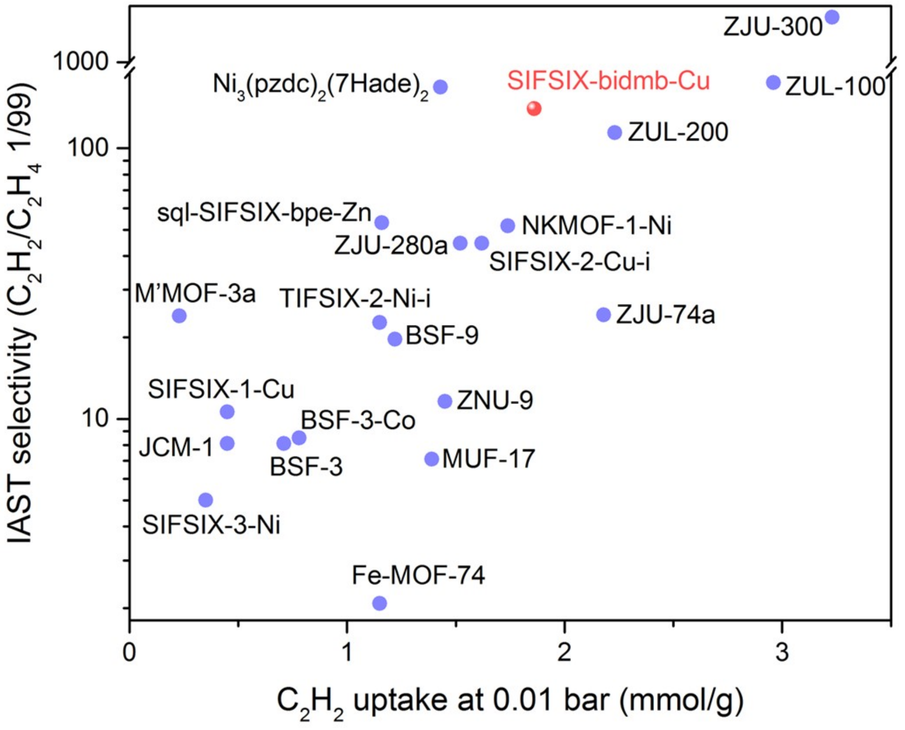

Figure S44 Comparison of the C_2H_2 uptakes at 0.01 bar with IAST selectivities of 1/99 C_2H_2/C_2H_4 mixture for various top-performing physisorbents.

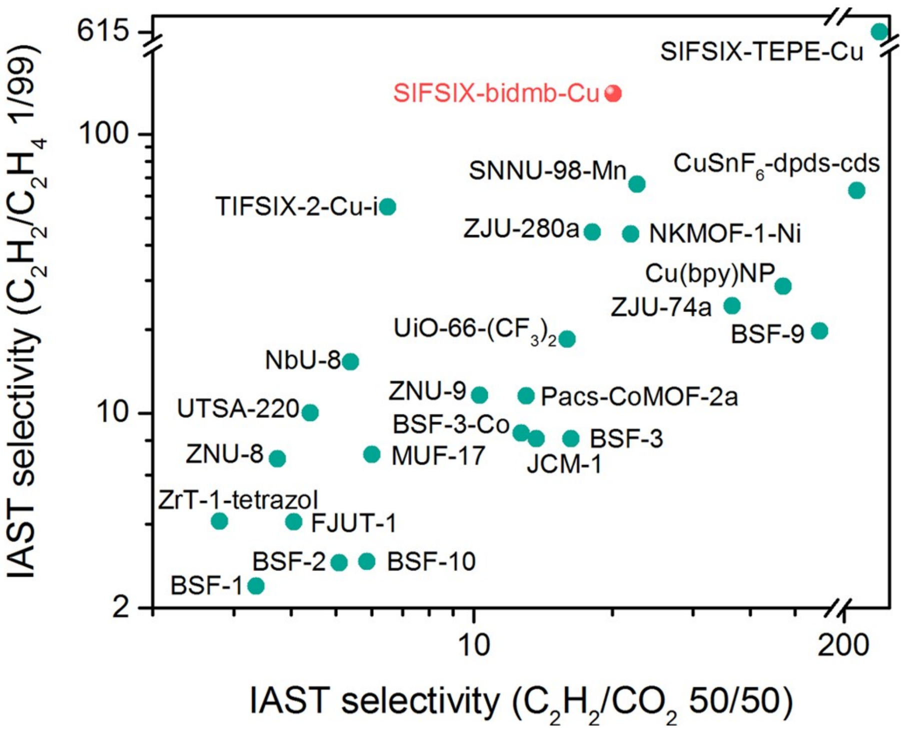

Figure S45 Comparison of IAST selectivities of 50/50 C_2H_2/CO_2 mixture and 1/99 C_2H_2/C_2H_4 for various top-performing physisorbents.

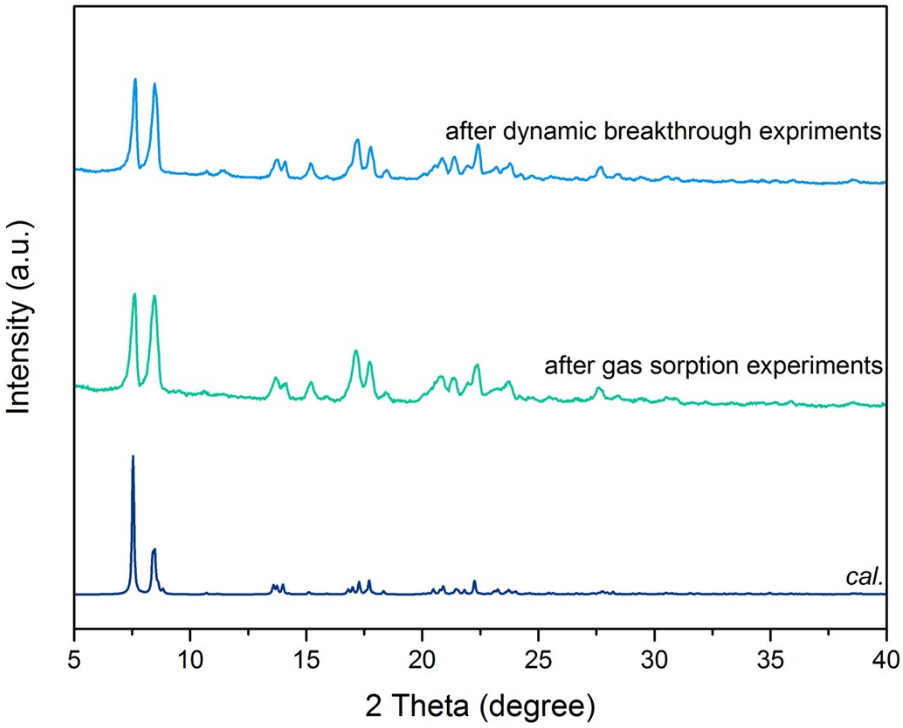

Figure S46 Comparison of PXRD pattern calculated from crystal structure of SIFSIX-bidmb-Cu with those measured for samples after consecutive sorption and breakthrough experiments.

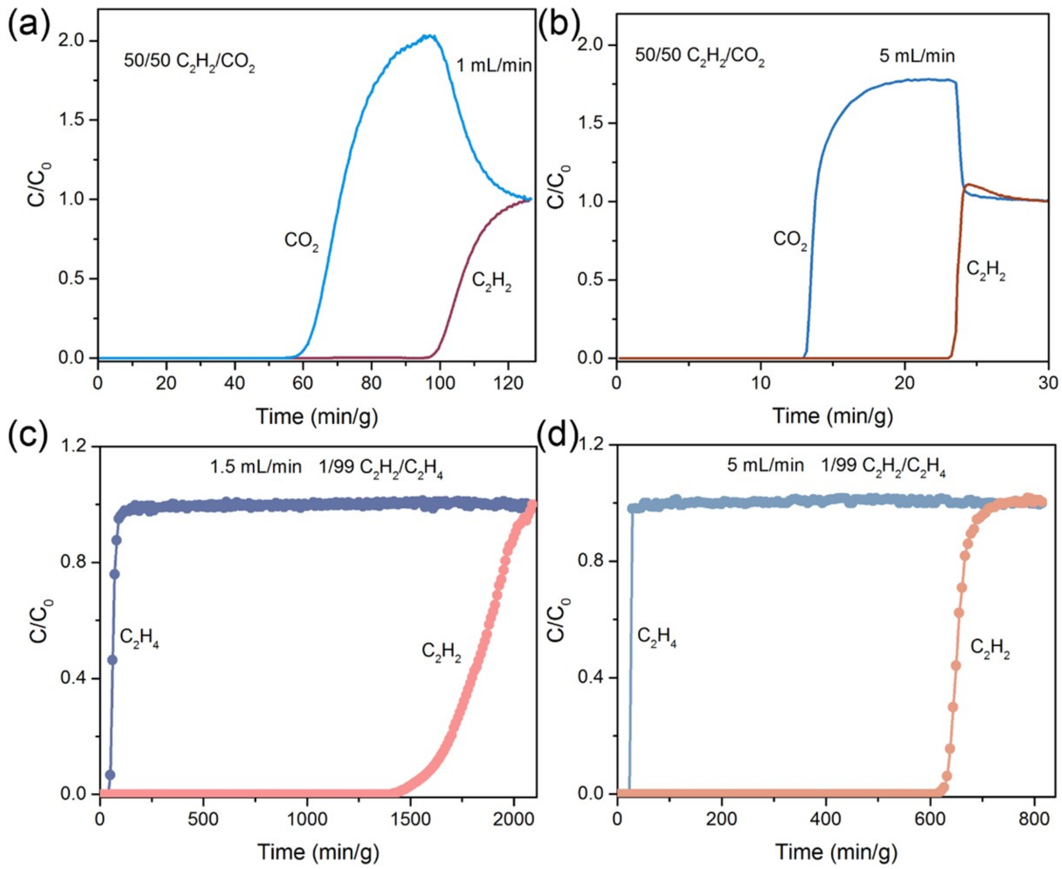

Figure S47 (a,b) The breakthrough separation experiments on SIFSIX-bidmb-Cu using 50/50 C_2H_2/CO_2 binary mixture at gas flow rates of 1 ml/min (a) and 5 ml/min (b), respectively. (c,d) The breakthrough separation experiments on SIFSIX-bidmb-Cu using 1/99 C_2H_2/C_2H_4 binary mixture at gas flow rates of 1.5 ml/min (c) and 5 ml/min (d), respectively.

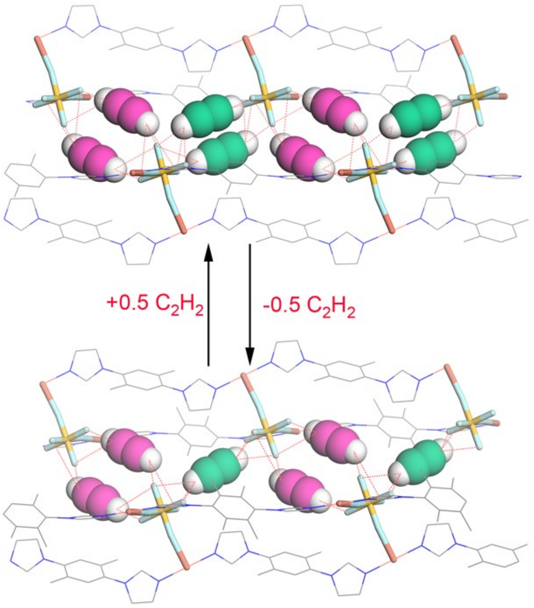

Figure S48 Considering the similar geometry of SiF_6^{2-} anion pairs on site I (pink) and site II (cyan), it can be inferred that additional C_2H_2 molecules can be adsorbed by C_2H_2@SIFSIX-bidmb-Cu' as the single C_2H_2(II) could possibly change orientation and deviate from the inversion center, leading to a similar binding mode to that of C_2H_2(I), i.e., two parallel and centrosymmetric C_2H_2

simultaneously chelated by two SiF_6^{2-} anions.[18] Based on DFT-D calculations, the chelation of two C_2H_2 molecules by two SiF_6^{2-} anions at both site I and site II is geometry reasonable.

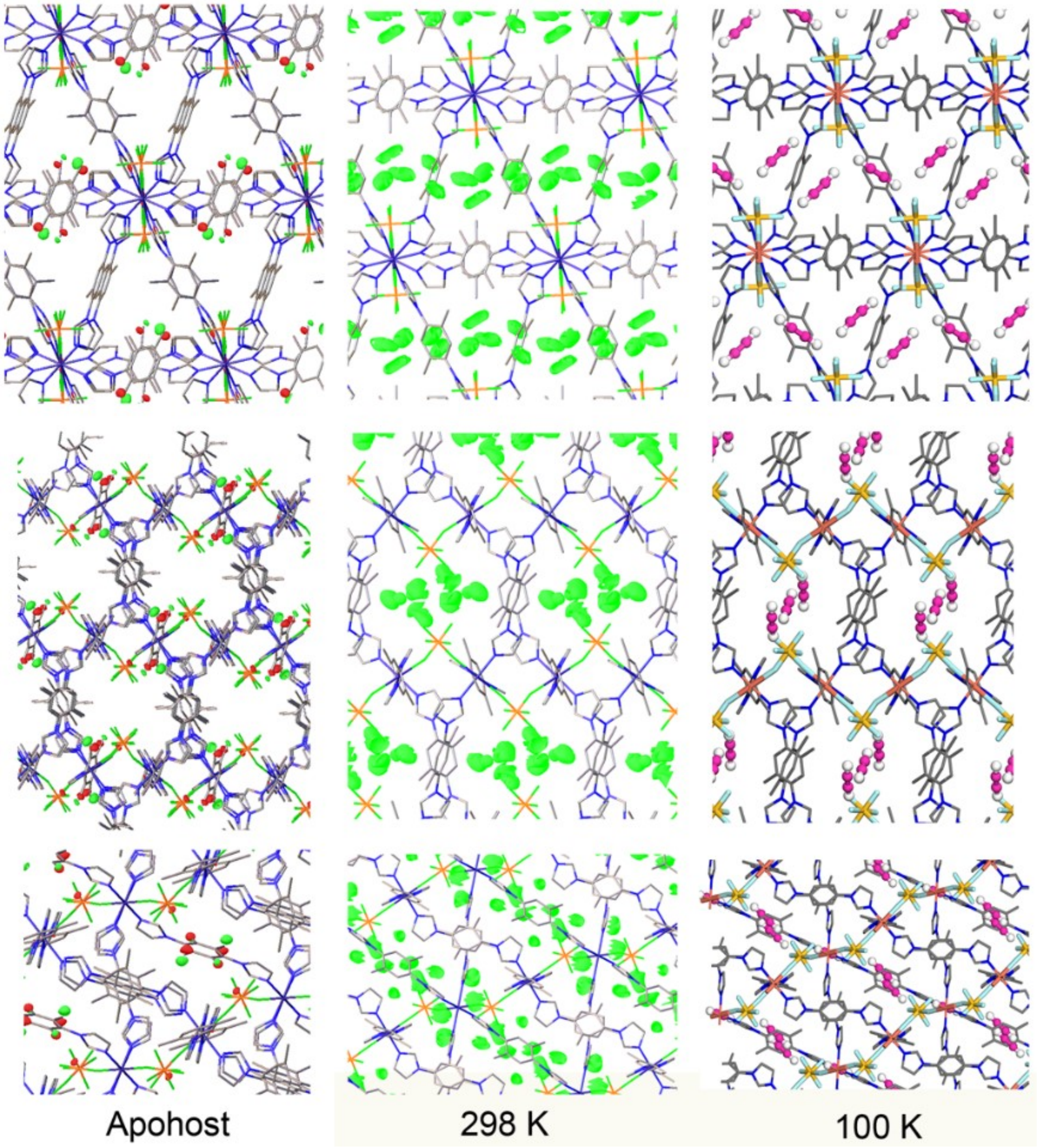

Figure S49 (left) The difference electron density map from apohost structure. (middle) The difference electron density map from in-situ SCXRD measurements with C_2H_2 at 298 K. (right) The C_2H_2 crystallographic positions determined from SCXRD at 100 K after crystals loaded with C_2H_2 at 298 K.

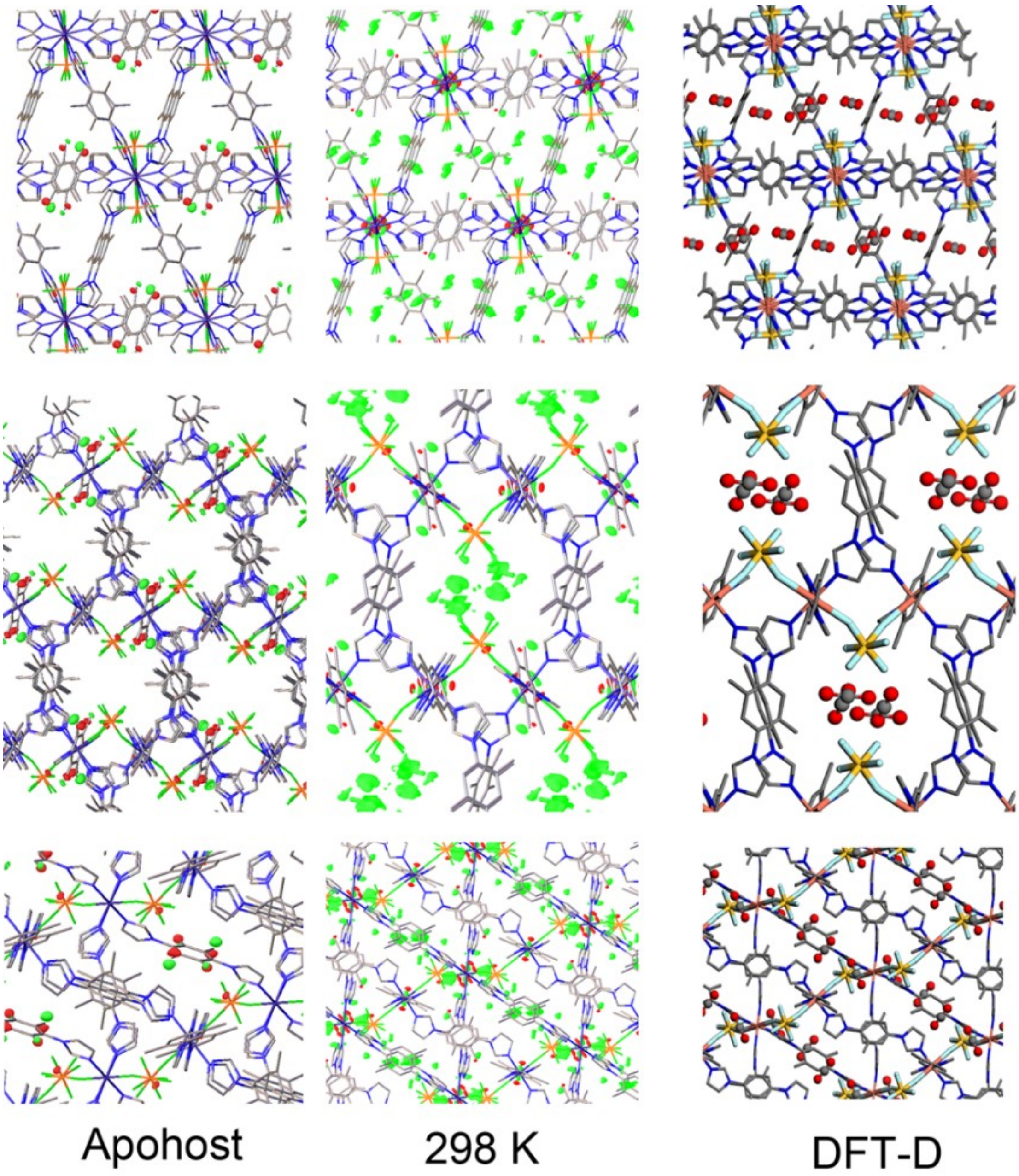

Figure S50 (left) The difference electron density map from apohost structure. (middle) The difference electron density map from in-situ SCXRD measurements with CO_2 at 298 K. (right) The CO_2 binding sites calculated from DFT-D calculations. Notably, the centrosymmetry of the host framework was considered during packing of CO_2 molecules in the channel.

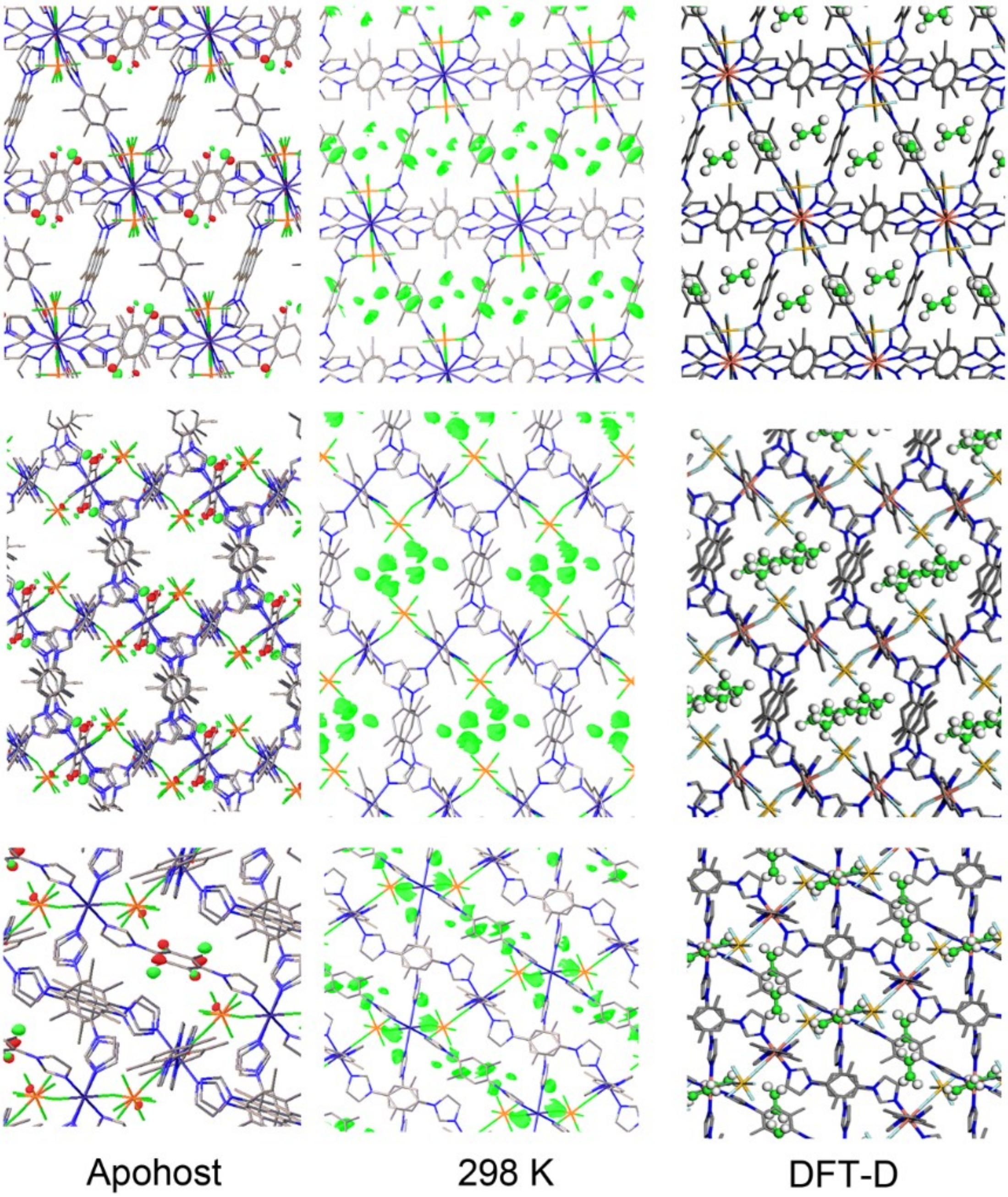

Figure S51 (left) The difference electron density map from apohost structure. (middle) The difference electron density map from in-situ SCXRD measurements with C_2H_4 at 298 K. (right) The C_2H_4 positions calculated from DFT-D. Notably, the centrosymmetry of the host framework was considered during packing of C_2H_4 molecules in the channel.

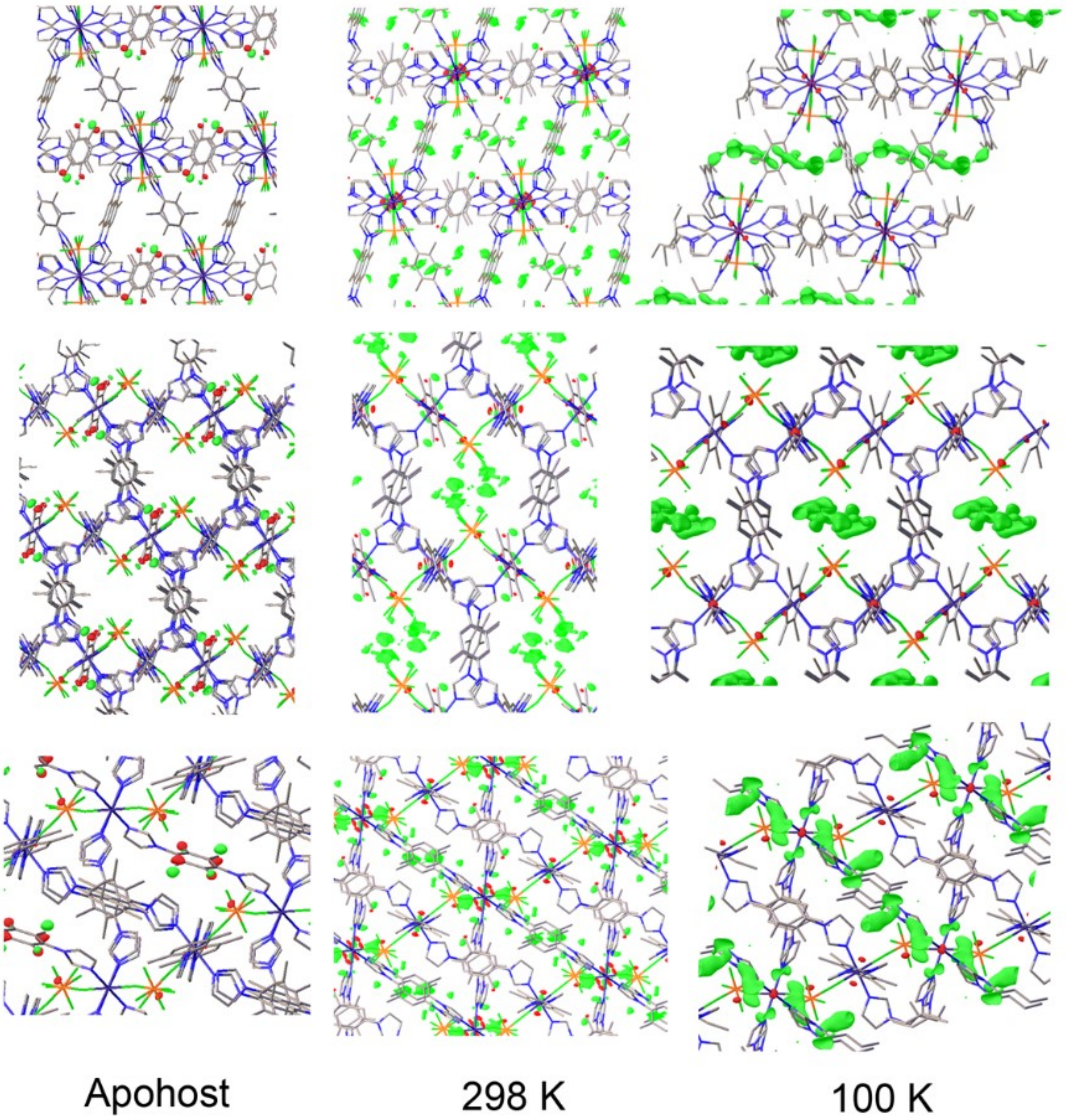

Figure S52 (left) The difference electron density map from apohost structure. (middle) The difference electron density map from in-situ SCXRD measurements with CO_2 at 298 K. (right) The difference electron density map from SCXRD studies at 100 K on crystals that were loaded with CO_2 at 298 K.

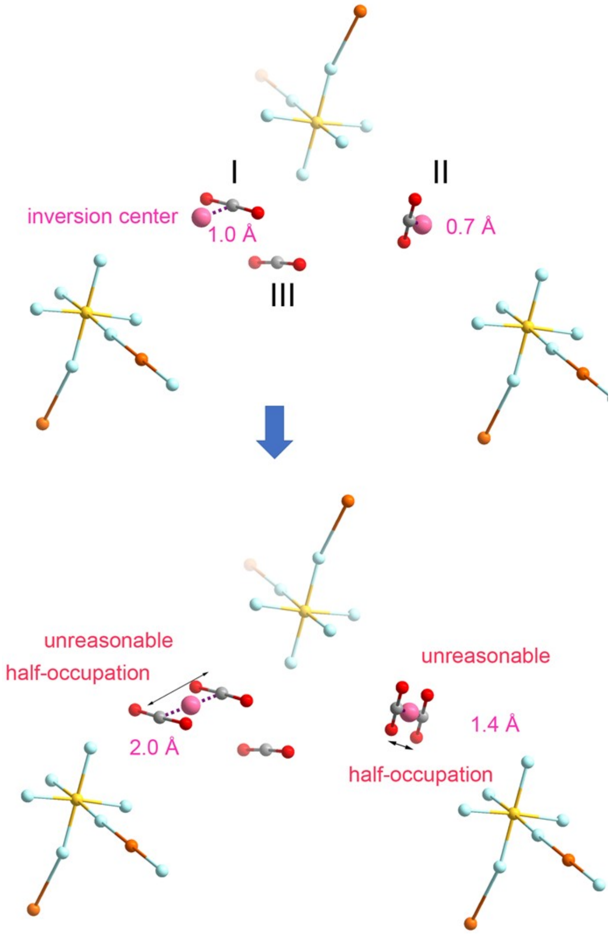

Figure S53 The CO_2 molecules at binding site I and II have half occupancy, as they are too close to the inversion centers between two SiF_6^{2-} anions (the light red balls) which will cause unreasonable close contact between two symmetric-related equivalents (bottom). This observation is in consistence with the results from CO_2 sorption isotherm.

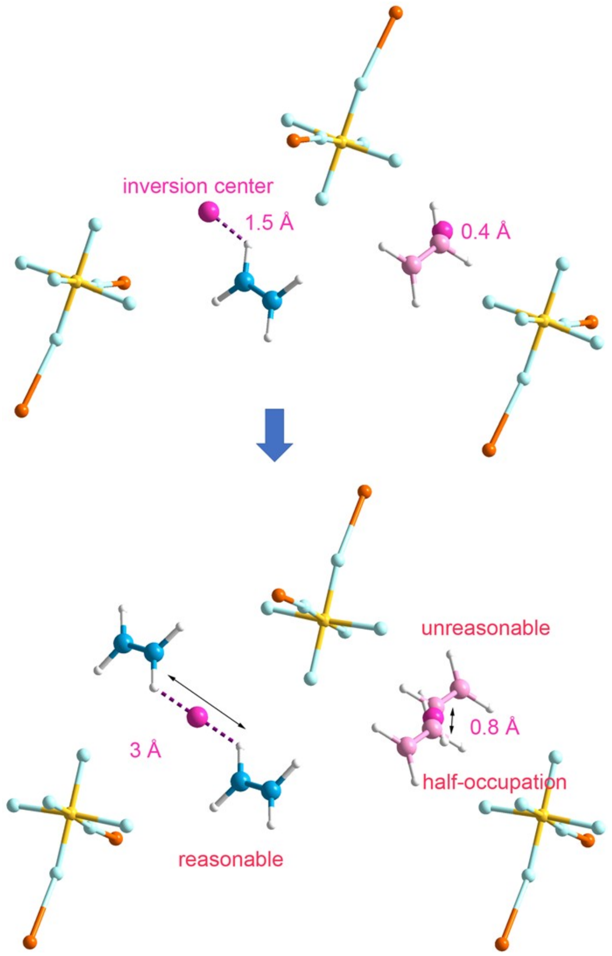

Figure S54 The C_2H_4 molecule at binding site II (pink) has half occupancy, as it is close to one inversion center between two SiF_6^{2-} anions (the light red ball) which will cause unreasonable close contact between two symmetric-related equivalents (bottom). This observation is in consistence with the results from C_2H_4 sorption isotherm.

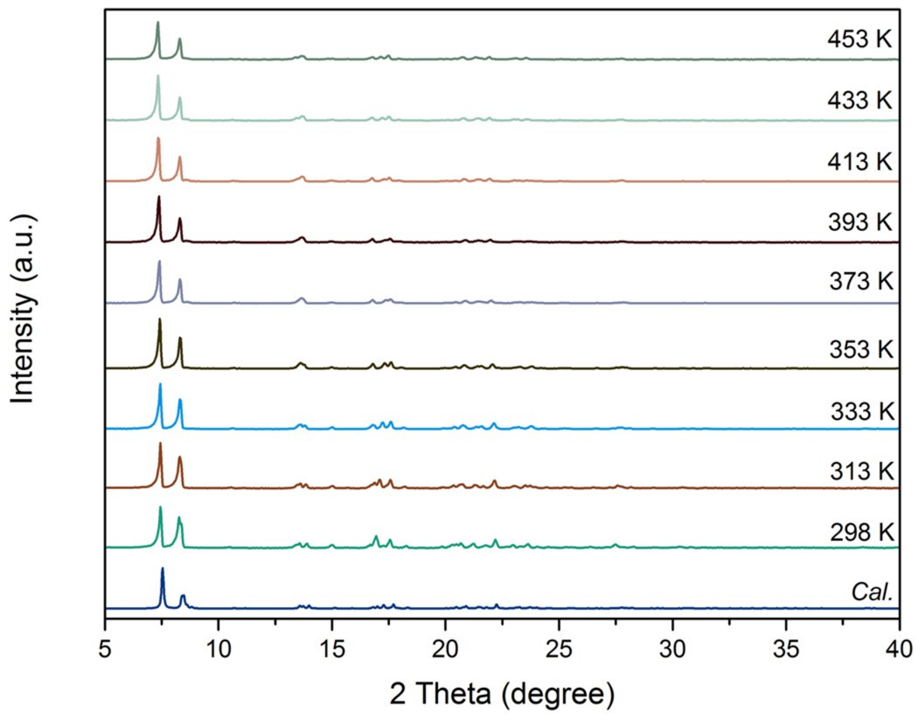

Figure S55 VT-PXRD patterns of sample in water for one year.

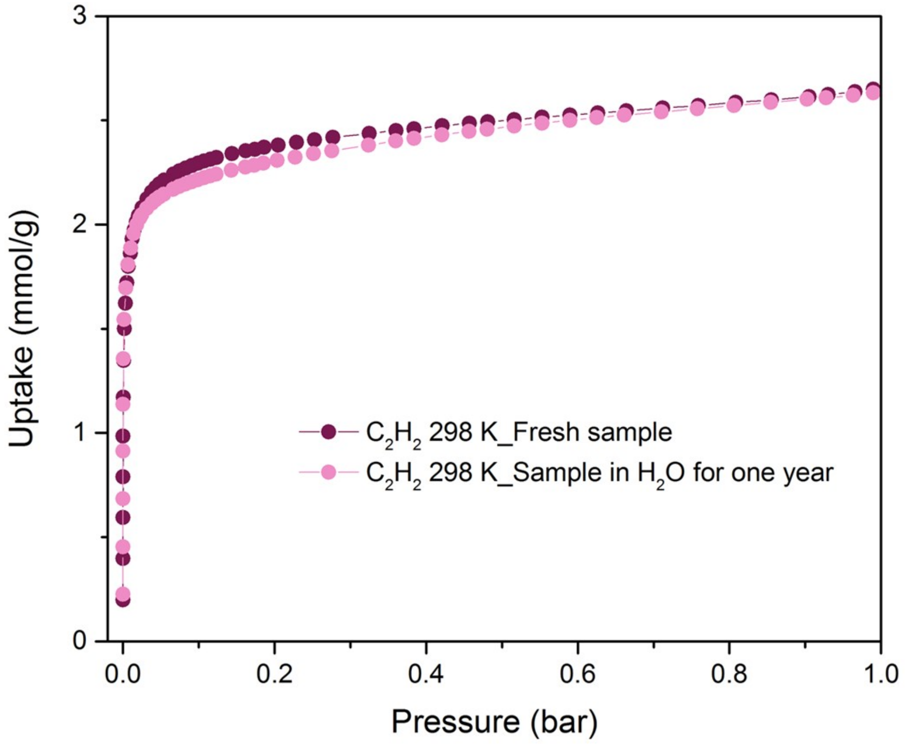

Figure S56 Comparison of C_2H_2 adsorption isotherm at 298 K of as-synthesized sample with that of sample in water for one year.

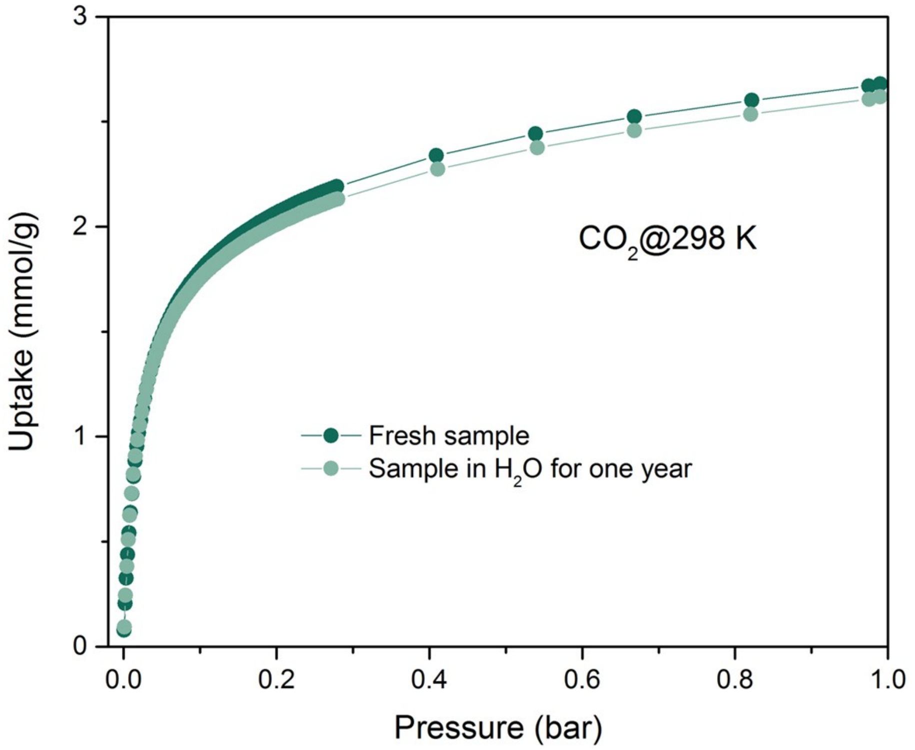

Figure S57 Comparison of CO_2 adsorption isotherm at 298 K of as-synthesized sample with that of sample in water for one year.

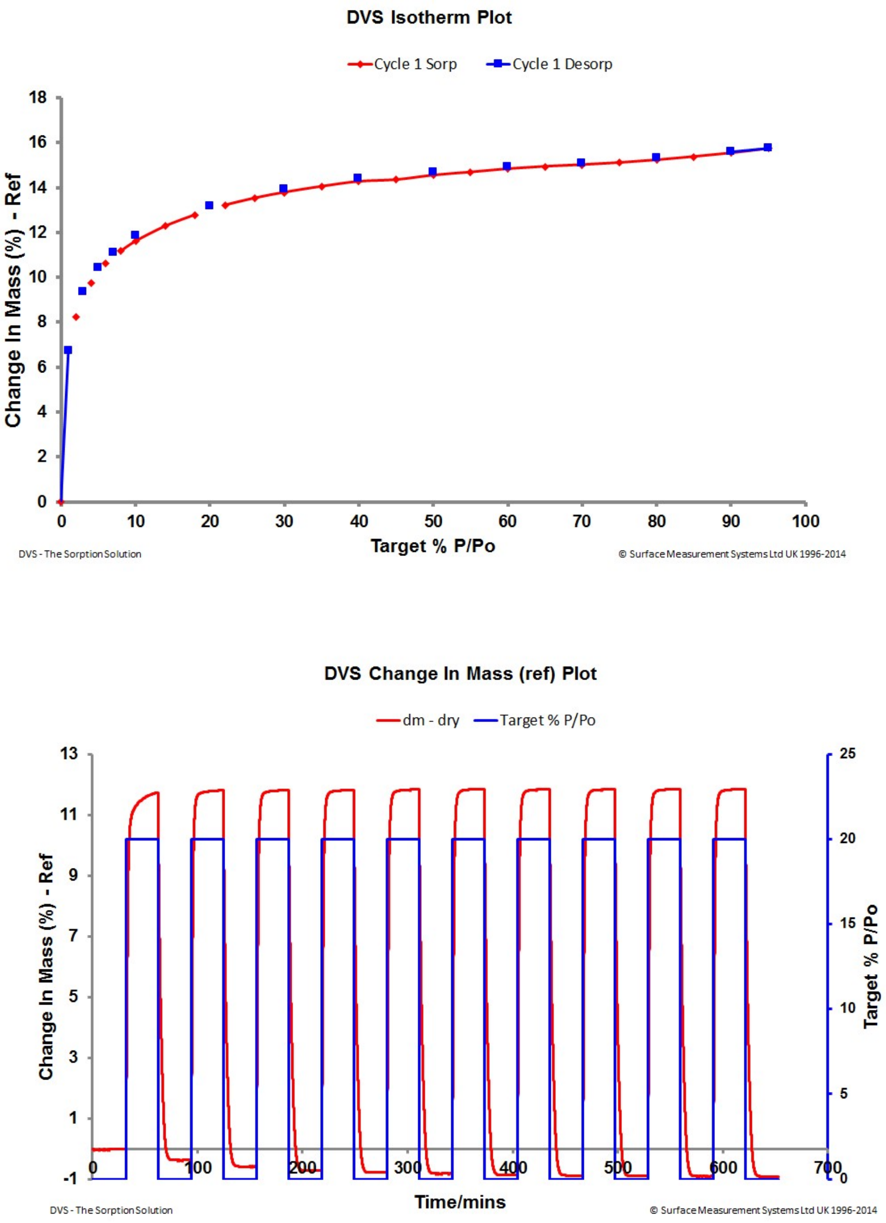

Figure S58 (Top) The water sorption isotherm at 298 K for SIFSIX-bidmb-Cu'. (Bottom) Ten continuous cycles of water adsorption-desorption at 298 K.

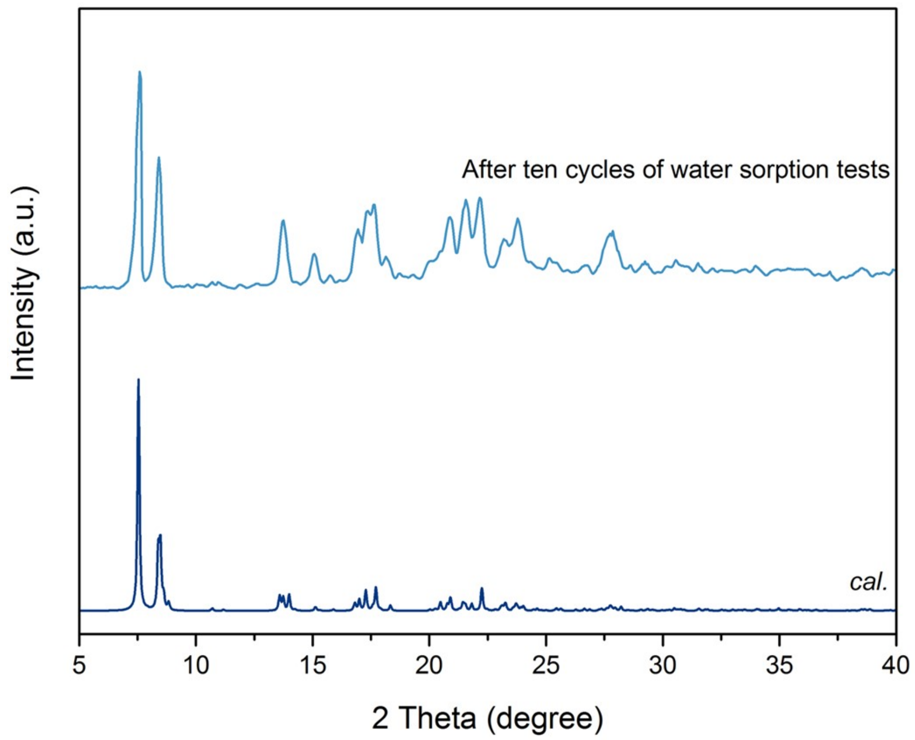

Figure S59 Comparison of PXRD calculated from crystal structure of SIFSIX-bidmb-Cu with that measured for sample after ten cycles of water sorption experiments.

4. Supporting Tables

Table S1 Crystallographic data and structure refinement summary for different structures.

Compound	SIFSIX-bidmb-Cu	SIFSIX-bidmb-Cu'	C_2H_2@SIFSIX-bidmb-Cu'[a]	CO_2@SIFSIX-bidmb-Cu'[b]
Formula	$C_{28}H_{28}CuF_6N_8Si$	$C_{28}H_{28}CuF_6N_8Si$	$C_{31}H_{29}CuF_6N_8Si$	$C_{28}H_{28}CuF_6N_8Si$
Formula weight	682.21	682.21	719.25	682.21
Temperature (K)	150.0	298 (2)	99.98(10)	100.00(10)
Crystal system	triclinic	triclinic	triclinic	triclinic
Space group	*P*-1	*P*-1	*P*-1	*P*-1
a (Å)	12.7494(5)	12.5516(7)	12.5344(2)	12.5027(2)
b (Å)	13.2904(6)	13.1903(7)	13.1083(3)	13.1215(2)
c (Å)	13.4525(5)	13.1903(7)	13.1083(3)	13.3760(2)
α (deg)	107.3570(10)	61.077(3)	60.939(2)	61.138(2)
β (deg)	101.7380(10)	77.461(3)	77.661(2)	77.6840(10)
γ (deg)	118.1650(10)	62.358(3)	62.396(2)	62.477(2)
V (Å^3)	1750.18(12)	1729.48(17)	1712.38(7)	1704.18(6)
Z	2	2	2	2
D_c (g·cm^{-3})	1.295	1.321	1.395	1.326
$\mu(mm^{-1})$	0.719	0.729	1.824	1.801
R_{int}	0.0165	0.0595	0.0231	0.0189
Data collected/unique	16744/7915	40059/6606	24859/6668	22113/6739
R_1 [$I > 2\sigma$ (I)][c]	0.0276	0.0474	0.0459	0.0416
wR_2 [all data][d]	0.0709	0.1378	0.1259	0.1078
GOF	1.042	1.054	1.066	1.046

**Compound**	**C_2H_2@SIFSIX-bidmb-Cu'_in-situ[e]**	**C_2H_4@SIFSIX-bidmb-Cu'_in-situ[f]**	**CO_2@SIFSIX-bidmb-Cu'_in-situ[g]**
Formula	$C_{28}H_{28}CuF_6N_8Si$	$C_{28}H_{28}CuF_6N_8Si$	$C_{28}H_{28}CuF_6N_8Si$
Formula weight	682.21	682.21	682.21
Temperature (K)	298 (2)	298 (2)	298 (2)
Crystal system	triclinic	triclinic	triclinic
Space group	*P*-1	*P*-1	*P*-1
a (Å)	12.9047(4)	12.7993(4)	12.6837(5)
b (Å)	13.3170(4)	13.3073(5)	13.3315(9)
c (Å)	13.4147(4)	13.4067(5)	13.4082(5)
α (deg)	107.9420(10)	107.373(2)	107.043(3)
β (deg)	101.0710(10)	101.567(2)	101.918(2)
γ (deg)	118.1910(10)	118.290(2)	118.388(3)
V (Å^3)	1769.51(9)	1752.89(11)	1738.32(16)
Z	2	2	2
D_c (g·cm^{-3})	1.28	1.293	1.308
$\mu(mm^{-1})$	0.712	0.718	0.725
R_{int}	0.0430	0.0488	0.0519
Data collected/unique	56836/8801	55897/8713	42742/8627
R_1 [$I > 2\sigma(I)$][a]	0.0380	0.0443	0.0604
wR_2 [all data][b]	0.0897	0.1150	0.1362
GOF	1.016	1.034	1.045

[a],[b]Gas loading at 298 K but SCXRD at 100K. For CO_2-loaded structure, the guests are highly disordered, thus, the relating electron density was removed to provide the guest-free structure.

[c]$R_1=\Sigma||F_o|-|F_c||/\Sigma|F_o|$. [d]$wR_2= |\Sigma w(|F_o|^2-|F_c|^2)|/\Sigma|w(F_o^2)^2|^{1/2}$

[e],[f],[g]In-situ gas loading and SCXRD at 298 K. The guests are highly disordered, thus, the relating electron density was removed to provide the guest-free structure.

Table S2 Comparison of SIFSIX-bidmb-Cu with the reported materials for C_2H_2/CO_2 separation.

Porous Materials	C_2H_2 uptake @ 0.01 bar (mmol/g)	CO_2 uptake @ 0.01 bar (mmol/g)	IAST (C_2H_2/CO_2) (50/50)	Qst (C_2H_2 kJ/mol)	Qst (CO_2 kJ/mol)	α_{AC}	Ref
**C_2H_2/CO_2 adsorption by molecular sieving mechanism**							
CPL-1-NH_2	0.2	< 0.1	119	50.0	33.0	-	[19]
SIFSIX-dps-Cu	0.75	0.11	1786.6	60.5	-	-	[20]
GeFSIX-dps-Cu	0.72	0.45	171.9	56.3	-	-	[20]
SIFSIX-dpm-Cu	1.74	0.41	10086	-	-	-	[21]
GeFSIX-dpm-Cu	1.71	0.36	3499	-	-	-	[21]
UTSA-300a	< 0.1	0.15	743	57.6	-	-	[22]
NTU-65	0.45	< 0.1	-	-	-	-	[23]
SOFOUR-TEPE-Zn	1.61	0.22	16833	45.6	26.3	-	[24]
HOF-FJU-1	< 0.5	< 0.1	6675	46.73	28.44	7.1	[25]
HOF-FJU-100	< 0.5	< 0.1	201	31.42	4.50	6.1	[26]
**C_2H_2/CO_2 adsorption by thermodynamic mechanism**							
SIFSIX-bidmb-Cu	1.86	0.54	20.3	55.7	20.1	7.8	This work
BNF-1	< 0.2	< 0.1	4.1[f]	30.2	24.3	4.1	[27]
BUT-316-a	0.27	0.14	17.2	35.4	14.0	-	[28]
Mn_3Mn_6-bco-tpbz	< 0.5	< 0.2	6.23	~ 32.5	~ 24.0	-	[29]
Co_3Co_6-bco-tppy	< 0.5	< 0.2	7.22	~ 35.0	~ 24.0	-	[29]
Co_3Co_6-bco-tpbz	< 0.5	< 0.2	6.80	~ 33.5	~ 24.0	-	[29]
Mn_3-bco-tpt	< 0.5	< 0.2	4.45	~ 30.0	~ 24.0	-	[29]
Co_3-bco-tpt	< 0.5	< 0.2	4.19	~ 31.5	~ 24.0	-	[29]
V-bdc-tpt	< 0.5	< 0.2	3.3	~ 29.0	~ 20.0	-	[30]
V-bcp-tppy	< 0.5	< 0.2	11.0	~ 44.0	~ 29.0	-	[30]
V-bcp-tpbz	< 0.5	< 0.2	10.1	~ 39.0	~ 37.0	-	[30]
V-chdc-tph	< 0.5	< 0.2	7.3	~ 41.0	~ 32.5	-	[30]
FJU-112a	< 0.5	< 0.2	4.2	32.1	23.1	3.0	[31]
HIAM-111	< 0.5	< 0.2	3	43.8	12.4	-	[32]
Ni(4-DPDS)$_2$$MoO_4$	2.04	< 0.5	67.7	75.4	37.0	6.7	[33]
Ni-Pz	< 0.5	< 0.2	10.8	41.9	29.2	-	[34]
Pt/Cu-PDA-ht	1.28	< 0.5	18[b]	36.5	21.8	-	[35]
Pd/Cu-PDA-ht	1.60	< 0.5	10[b]	54.9	26.3	-	[35]
SOFOUR-2-Zn	< 0.5	< 0.2	8.2	33.3	22.1	2.05	[36]
TIFSIX-Cu-POZ	< 0.2	< 0.1	4.35	30.3	25.3	-	[37]
UPC-COF-1	-	-	4.5	34.9	25.4	-	[38]
ZNU-12	1.05	0.21	13.4	36.1	31.9	-	[39]
ZNU-9	1.45	0.36	10.3	33.1	26.6	-	[40]

ZNU-8	0.34	0.21	3.74	27.2	23.4	-	[40]
BSF-1	< 0.5	< 0.2	3.3	31	22	-	[41]
BSF-2	< 0.5	< 0.2	5.1	37.3	28.7	-	[42]
BSF-3	< 0.8	< 0.5	16.3	42.7	22.4	16	[43]
BSF-3-Co	< 0.8	< 0.5	12.7	-	-	-	[43]
BSF-4	< 0.2	< 0.1	9.8	35	-	-	[44]
BSF-10	-	-	5.86	34.8	22.9	2.8	[45]
ZNU-1	1.22	0.05	56.6	54.0	44.0	49	[46]
ZNU-4	< 0.5	< 0.2	8.9	50.28	34.57	5.4	[12a]
UTSA-74a	< 0.5	< 0.2	9	32	25	20.1	[47]
UTSA-50[a]	< 0.5	< 0.2	13.3	39.4	27.8	-	[48]
UTSA-68[a]	< 0.2	< 0.1	3.3	25.8	-	-	[49]
UTSA-98	< 0.2	< 0.1	5.2	22.8	18.3	-	[50]
UTSA-222a[a]	< 0.5	< 0.2	2	26	17	-	[51]
PCP-31	< 0.5	< 0.2	7	53	30	-	[52]
PCP-32	< 0.5	< 0.2	8	36	26	-	[52]
PCP-33	< 0.5	< 0.2	6	27.5	26	-	[53]
iMOF-5C	< 0.1	< 0.1	6	35.5	-	-	[54]
iMOF-6C	< 0.1	< 0.1	8	38	-	-	[54]
iMOF-7C	< 0.2	< 0.2	4	35	-	-	[54]
JCM-1	< 1	< 0.2	13.7	36.9	33.4	4.4	[55]
SIFSIX-Cu-TPA	1.56	1.21	5.3	39.1	25.7	-	[56]
SIFSIX-21-Ni	< 1	< 0.2	27.7	37.9	19.8	27.7	[57]
TIFSIX-2-Ni-i	1.15	0.61	6.1	40	34	-	[58]
SIFSIX-17-Ni	0.55	0.2	11.7	44.2	40.2	-	[59]
TIFSIX-17-Ni	0.54	0.32	20.9	48.3	37.8	-	[59]
UPC-110	< 0.5	< 0.5	5.1	24.6	13	-	[60]
MUF-15[b]	< 0.3	< 0.5	4.2	24.2	28.3	-	[61]
MUF-17[b]	1.39	0.86	6.01	49.5	31.1	-	[62]
TCuI	0.15	-	5.3	38.4	26.6	33.4	[63]
TCuBr	0.21	-	-	36.6	30.2	104.5	[63]
TCuCl	0.51	-	16.9	41	30.1	143.1	[63]
CAU-10-H[a]	< 0.5	< 0.2	4	27	25	3.4	[64]
CAU-10-NH_2	< 0.5	< 0.2	10.8	31.3	24.5	-	[65]
MIL-160	1.10	0.43	10	31.8	26.9	-	[66]
CAU-23	< 0.5	< 0.2	3.8	26.7	20	-	[66]
MOF-NH2	< 0.5	< 0.2	12.6	16.7	24.2	-	[67]
MOF-OH	< 0.5	< 0.2	25	17.5	20.6	-	[67]
SNNU-37(Fe)	< 0.5	< 0.2	9.9	35	33.6	-	[68]
SNNU-37(Sc)	< 0.5	< 0.2	2.7	34.4	33.4	-	[68]
SNNU-45	0.89	< 0.5	8.5	39.9	27.1	2.9	[69]
SNNU-63	< 0.5	< 0.2	3.3	21.6	21.95	-	[70]
SNNU-65-Cu-Sc	< 0.5	< 0.2	13.5	44.9	22.2	-	[71]

SNNU-65-Cu-Fe	< 0.5	< 0.2	6.7	28.2	21.8	-	[71]
SNNU-65-Cu-Ga	< 0.5	< 0.2	18.7	31.7	20.5	-	[71]
SNNU-65-Cu-In	< 0.5	< 0.2	7	23.4	24.9	-	[71]
SNNU-150-Al	< 0.2	< 0.1	7.27	29	24.8	1.97	[72]
SNNU-150-Ga	< 0.2	< 0.1	4.93	33	28	-	[72]
SNNU-150-In	< 0.2	< 0.1	5.57	36	32	-	[72]
ZJU-60[a]	< 0.5	< 0.2	6.7	17.6	15.2	-	[73]
ZJU-74a[a]	2.18	< 0.05	36.5	44.5	30	4.3	[74]
ZJU-199[a]	< 0.2	< 0.2	4	38.5	29	-	[75]
ZJUT-2[a]	< 1	< 0.5	10	41.5	36.1	-	[76]
PCM-48[a]	< 0.2	< 0.2	4.3	23.6	15.4	-	[77]
JXNU-12	< 0.5	< 0.2	2	21.3	19.9	-	[78]
JXNU-12(F)	< 0.5	< 0.2	4.1	28	19.7	-	[78]
NbU-8[b]	< 0.5	< 0.2	5	34.6	30.3	-	[79]
NbU-10[b]	< 0.2	< 0.2	6.5	34.6	27.6	-	[80]
FJU-6-TATB[a]	< 0.5	< 0.2	3.1	29	26	2.3	[81]
FJU-89a	< 0.5	< 0.2	4.3	31	27.8	3.0	[82]
FJU-90a	< 1	< 0.5	4.3	25.1	20.7	2.1	[83]
ZJU-195	< 0.5	< 0.2	4.7	29.9	20.7	-	[84]
Cu^{I}@UiO-66-$(COOH)_2$	0.9	0.03	185	74.5	28.9	3.4	[85]
Cu-CPAH	< 0.5	< 0.2	3.6	35.4	31.5	-	[86]
FeNi-M'MOF	< 1	< 0.3	24	27	24.5	1.7	[87]
NKMOF-1-Ni	1.74	0.45	25	60.3	40.9	2.6	[88]
IPM-101	1.53	0.25	12.3	43.7	30.7	22.5	[89]
DICRO-4-Ni-i	< 0.5	< 0.2	13.9	37.7	33.9	-	[90]
CPM-107op	< 0.5	< 0.2	5.7	32	24	-	[91]
$[Ni_3(HCOO)_6]$	1.01	0.23	22	40.9	24.5	-	[92]
NTU-54[c]	< 0.1	< 0.1	6.3	38	35	-	[93]
NTU-55	< 0.5	< 0.2	4.5	25.5	22	-	[94]
NTU-66-Cu	< 0.5	< 0.2	6	32.3	21.7	-	[95]
HOF-3[a]	< 0.5	< 0.2	21	42	19	2	[95]
[Ni(dpip)]·2.5DMF·H_2O	< 0.5	< 0.2	2.9	41.7	30.3	-	[95]
[Ni(tzba)0.5(F)(bpy)]	< 0.5	< 0.2	3	36.7	25.6	4.5	[96]
M′MOF-2a[d]	< 0.5	< 0.2	1.89	37.7	32.5	-	[97]
M′MOF-3a[d]	< 0.5	< 0.2	8.41	27.1	40.5	-	[97]
$Cu_2(ade)_2(PA)_2$	< 0.5	< 0.2	4.1	26.8	23.6	-	[32]
JNU-1	< 1	< 0.5	3	13	-	-	[98]
JNU-2	< 0.5	< 0.2	3.5	15.8	13.5	-	[98]
JNU-4	< 0.5	< 0.2	5.4	26.8	19.7	12.8	[99]
UPC-200(Al)-F-BIM	< 0.5	< 0.2	2.25	20.5	14	-	[100]
SDU-CP-1[c]	< 0.5	< 0.2	2.5	27.9	21.4	-	[101]
MPM-1-Cl	< 0.5	< 0.2	3.21	27.3	23.1	-	[102]
$Ca(dtztp)_{0.5}(DMA)\cdot 2H_2O$	< 0.5	< 0.2	1.8	28.8	19.2	-	[103]

Cu(BDC-Br)	< 0.5	< 0.2	3.9	26.1	25.6	2.8	[104]
JXNU-5a	< 0.5	< 0.2	5	32.9	25.2	9.9	[105]
ATC-Cu	1.61	0.43	53.6	79.1	-	-	[106]
FJU-22a	< 0.5	< 0.2	-	23	-	-	[12b]
FJU-36	< 0.5	< 0.2	2.8	32.9	31.1	8.7	[107]
ZJNU-13	< 0.5	< 0.2	5.64	33.5	22.5	-	[108]
[Ni_2(BTEC)$(bipy)_3$]	< 0.5	< 0.1	33.5	19.8	25	-	[109]
Zn_2(Pydc)$(Ata)_2$	< 0.5	< 0.2	3..9	43.1	32.1	-	[110]
MAF-2	< 0.5	< 0.2	-	29.8	25.8	-	[111]
sql-16-Cu-NO_3	< 0.5	< 0.2	78	38.6	25.6	78	[112]
a: At temperature of 296 K; b: At temperature of 293 K; c: IAST(C_2H_2/CO_2=2:1); d: At temperature of 295 K; e: At temperature of 273 K; - = Not available; f: Selectivity from breakthrough experiments.							

Table S3 Comparison of SIFSIX-bidmb-Cu with the reported materials for C_2H_2/C_2H_4 separation.

Porous Materials	C_2H_2 uptake @ 0.01 bar (mmol/g)	C_2H_4 uptake @ 0.01 bar (mmol/g)	IAST (C_2H_2/ C_2H_4) (1/99)	Qst (C_2H_2 kJ/mol)	Qst (C_2H_4 kJ/mol)	α_{AE}	Ref
**C_2H_2/C_2H_4 adsorption by molecular sieving mechanism**							
SIFSIX-14-Cu-i	1.80	0.03	6320	56	27	91	[113]
UTSA-300-Cu	0.73	0.04	7291	60.5	-	-	[114]
**C_2H_2/C_2H_4 adsorption by thermodynamic mechanism**							
SIFSIX-bidmb-Cu	1.86	0.23	140.2	55.7	38.1	105.7	This work
a-dCoM	< 0.2	< 0.2	-	-	-	-	[115]
g-dCoM	< 0.2	< 0.2	-	-	-	-	[115]
HOF-TDCPB	< 0.1	< 0.1	1.51	24.8	21.3	-	[116]
MOF-303[a]	< 0.5	< 0.2	2.4	31.7	24.3	-	[117]
PMOF-2	< 1	< 0.5	-	-	-	-	[118]
ZJU-300a[a]	3.23	< 0.5	1672	61.1	39.7	264	[119]
ZNU-9	1.45	0.11	11.64	33.1	25.75	-	[40]
$Ni_3(pzdc)_2(7Hade)_2$	1.43	0.26	168	44.5	35.7	25	[120]
ZNU-8	0.34	0.04	6.86	27.2	22.5	-	[40]
BSF-3	< 0.5	< 0.3	8.1	42.7	27.4	-	[43]
BSF-3-Co	0.78	0.19	8.5	-	-	-	[43]
SIFSIX-1-Cu	0.44	0.02	11	30	23.5	12.4	[121]
Fe-MOF-74	1.15	< 0.2	2.08	47	-	-	[122]
Co-MOF-74	1.01	< 0.2	1.7	45	-	-	[122]
Mg-MOF-74	1.13	< 0.2	2.18	41	-	-	[122]
UTSA-100a[a]	0.76	0.28	10.72	22	-	35	[123]
ZUL-100	2.96	0.25	175	65.3	< 40	-	[124]
ZUL-200	2.23	0.15	100	57.6	< 40	-	[124]
NOTT-300[c]	0.29	0.11	2.17	32	16	-	[125]
SIFSIX-2-Cu-i	1.64	0.07	55	41.9	30.7	50	[126]
TIFSIX-2-Cu-i	1.69	0.1	55	46	36	-	[126]
NbOFFIVE-2-Cu-i	1.52	0.04	29	52	34	-	[126]
SIFSIX-2-Cu	0.15	0.02	6	26.3	20.8	-	[121]
SIFSIX-3-Zn	0.8	0.05	8.8	31	28.8	10	[121]
SIFSIX-3-Ni	0.51	0.04	5.03	30.5	20.3	-	[121]
M'MOF-3a[b]	0.23	0.02	24	27.1	-	-	[97]
ZJU-280a[a]	1.52	0.23	44.5	50.6	32.9	-	[127]
UTSA-220	< 1	< 0.5	10	29	24	-	[128]
ZNU-4	0.85	0.21	11.8[d]	50.3	36.7	-	[12a]
a: At temperature of 296 K; b: At temperature of 295 K; c: At temperature of 293 K; d: 50/50; - = not available.							

Table S4 Comparison of SIFSIX-bidmb-Cu with the reported materials for both C_2H_2/CO_2 and C_2H_2/C_2H_4 separation.

Porous Materials	C_2H_2 uptake @ 0.01 bar (mmol/g)	IAST (C_2H_2/ CO_2) (50/50)	IAST (C_2H_2/ C_2H_4) (1/99)	Qst (C_2H_2 kJ·mol^{-1})	Qst (CO_2 kJ·mol^{-1})	Qst (C_2H_4 kJ·mol^{-1})	Ref
SIFSIX-bidmb-Cu	1.86	20.3	140.2	55.7	20.1	38.1	This work
ZNU-9[a]	1.45	10.3	11.64	33.1	25.75	26.6	[40]
ZNU-8[a]	0.34	3.74	6.86	27.2	23.4	22.5	[40]
BSF-3	0.49	16.29	8.1	42.7	22.4	28.1	[43]
CdMOF[a]	0.33	2.3	2.5	21.2	19.6	18.8	[129]
Cr-btc-tpt[a]	0.47	4.1	2.3	~ 30	~ 35	~ 31	[130]
Cu(bpy)NP	1.00	47.2	28.5	40.8	14.6	31.3	[131]
Cu-DIN-SO4	0.37	15.5	30.5	58.11	34.72	28.66	[132]
$CuSnF_6$-dpds-cds	2.17	243.9	62.9	42.2	35.5	35	[133]
FJI-H36	0.89	3.7	2.4	36.1	29.2	29.9	[134]
FJUT-1	1.25	4.06	4.07	43.75	37.39	31.01	[135]
HIAM-111[a]	0.51	2.42	1.62	43.8	12.4	25.5	[32]
iMOF-5C	0.33	5	14	35.5	-	-	[54]
iMOF-6C	0.41	10	25	38	-	-	[54]
iMOF-7C	0.24	4	8	35	-	-	[54]
In-L6-IPA[a]	0.37	4.3	2.9	32.2	28.3	30.3	[136]
MUF-17[b]	1.39	6.01	8.73	49.5	33.8	31.1	[62]
NbU-8[b]	0.24	5.4	15.3	34.5	30	25.1	[79]
NbU-10[b]	0.32	6.5	2.8	31.26	27.58	34.61	[80]
NKMOF-1	1.74	22	44	60.3	44.9	40.9	[88]
$[Cu_{1.5}F(SiF_6)(L)_{2.5}]$[a]	0.8	9.3	26.4	42.3	39.6	34.4	[137]
NUM-15[a]	0.31	2.8	2.4	37.4	33.2	34.2	[138]
QDU-MOF-1	0.55	2.95	2.67	64.14	52.26	46.89	[139]
SIFSIX-DPA-Cu-i	0.89	9.34	8.93	46.53	26.35	27.21	[140]
SIFSIX-TEPE-Cu	3.48	382.9	615.2	45.7	36.9	28.5	[141]
SNNU-98-Mn	1.56	22.7	66.1	60.2	29.2	30.8	[142]
SIFSIX-17[a]	0.91	11.7	506.4	44.2	40.2	-	[59]
TIFSIX-2-Ni-i	1.15	6.2	22.7	40	34	31	[58]
UiO-66-$(CF_3)_2$	0.73	16	18	43	-	-	[143]
UPC-80[a]	-	6.34	4.87	20.84	17.64	18.37	[144]
ZJU-74a[c]	2.19	76	45	45	30	39	[74]
ZJU-280a	1.52	18.1	44.5	50.6	38.8	32.9	[127]
ZNU-11	1.0	8.2	30.3	36.1	33.9	32.9	[145]
ZrT-1-tetrazol	0.43	2.83	4.05	33.27	29.89	29.87	[146]
a: IAST (C_2H_2/C_2H_4:50/50); b: 293 K; c: 296 K							

5. Supporting References

[1] a) B.-Q. Song, Q.-Y. Yang, S.-Q. Wang, M. Vandichel, A. Kumar, C. Crowley, N. Kumar, C.-H. Deng, V. GasconPerez, M. Lusi, *J. Am. Chem. Soc.* **2020**, *142*, 6896-6901; b) G.-S. Yang, H.-Y. Zang, Y.-Q. Lan, X.-L. Wang, C.-J. Jiang, Z.-M. Su, L.-D. Zhu, *CrystEngComm* **2011**, *13*, 1461-1466; c) J. Fan, L. Gan, H. Kawaguchi, W.-Y. Sun, K.-B. Yu, W.-X. Tang, *Chem. - Eur. J.* **2003**, *9*, 3965-3973.

[2] Version 2017.3-0 ed., APEX3. Bruker AXS Inc., Madison, Wisconsin, USA, **2017**.

[3] Version 2014/4 ed., SADABS. Bruker AXS Inc., Madison, Wisconsin, USA, **2014**.

[4] Version 2014/2 ed., XPREP. Bruker AXS Inc., Madison, Wisconsin, USA, **2014**.

[5] G. Sheldrick, *Acta Cryst. A* **2015**, *71*, 3-8.

[6] G. Sheldrick, *Acta Cryst. C* **2015**, *71*, 3-8.

[7] O. V. Dolomanov, L. J. Bourhis, R. J. Gildea, J. A. K. Howard, H. Puschmann, *J. Appl. Crystallogr.* **2009**, *42*, 339-341.

[8] A. Spek, *Acta Cryst. C* **2015**, *71*, 9-18.

[9] A. L. Spek, *Acta Cryst. D* **2009**, *65*, 148-155.

[10] SAINT and SADABS. Bruker AXS Inc, Madison, WI, USA, **2016**.

[11] a) L. J. Barbour, *J. Appl. Crystallogr.* **2020**, *53*, 1141-1146; b) L. J. Barbour, *J. Supramol. Chem.* **2001**, *1*, 189-191.

[12] a) N. Xu, J. Hu, L. Wang, D. Luo, W. Sun, Y. Hu, D. Wang, X. Cui, H. Xing, Y. Zhang, *Chem. Eng. J.* **2022**, *450*, 138034; b) Z. Yao, Z. Zhang, L. Liu, Z. Li, W. Zhou, Y. Zhao, Y. Han, B. Chen, R. Krishna, S. Xiang, *Chem. - Eur. J.* **2016**, *22*, 5676-5683.

[13] K.-J. Chen, Hayley S. Scott, David G. Madden, T. Pham, A. Kumar, A. Bajpai, M. Lusi, Katherine A. Forrest, B. Space, John J. Perry, Michael J. Zaworotko, *Chem* **2016**, *1*, 753-765.

[14] A. L. Myers, J. M. Prausnitz, *AIChE J.* **1965**, *11*, 121-127.

[15] J. P. Perdew, K. Burke, M. Ernzerhof, *Phys. Rev. Lett.* **1997**, *78*, 1396-1396.

[16] a) S. J. Clark, M. D. Segall, C. J. Pickard, P. J. Hasnip, M. I. J. Probert, K. Refson, M. C. Payne, *Zeitschrift für Kristallographie - Crystalline Materials* **2005**, *220*, 567-570; b) M. D. Segall, J. D. L. Philip, M. J. Probert, C. J. Pickard, P. J. Hasnip, S. J. Clark, M. C. Payne, *J. Phys.: Condens. Matter* **2002**, *14*, 2717.

[17] S. Bhattacharya, K. E. Gubbins, *Langmuir* **2006**, *22*, 7726-7731.

[18] T. Jacobs, G. O. Lloyd, J.-A. Gertenbach, K. K. Müller-Nedebock, C. Esterhuysen, L. J. Barbour, *Angew. Chem. Int. Ed.* **2012**, *51*, 4913-4916.

[19] L. Yang, L. Yan, Y. Wang, Z. Liu, J. He, Q. Fu, D. Liu, X. Gu, P. Dai, L. Li, X. Zhao, *Angew. Chem. Int. Ed.* **2021**, *60*, 4570-4574.

[20] J. Wang, Y. Zhang, Y. Su, X. Liu, P. Zhang, R.-B. Lin, S. Chen, Q. Deng, Z. Zeng, S. Deng, B. Chen, *Nat. Commun.* **2022**, *13*, 200.

[21] X. Zhu, T. Ke, J. Zhou, Y. Song, Q. Xu, Z. Zhang, Z. Bao, Y. Yang, Q. Ren, Q. Yang, *J. Am. Chem. Soc.* **2023**, *145*, 9254-9263.

[22] R.-B. Lin, L. Li, H. Wu, H. Arman, B. Li, R.-G. Lin, W. Zhou, B. Chen, *J. Am. Chem. Soc.* **2017**, *139*, 8022-8028.

[23] Q. Dong, X. Zhang, S. Liu, R.-B. Lin, Y. Guo, Y. Ma, A. Yonezu, R. Krishna, G. Liu, J. Duan,

R. Matsuda, W. Jin, B. Chen, *Angew. Chem. Int. Ed.* **2020**, *59*, 22756-22762.

[24] X. Liu, P. Zhang, H. Xiong, Y. Zhang, K. Wu, J. Liu, R. Krishna, J. Chen, S. Chen, Z. Zeng, S. Deng, J. Wang, *Adv. Mater.* **2023**, *35*, 2210415.

[25] Y. Yang, H. Zhang, Z. Yuan, J.-Q. Wang, F. Xiang, L. Chen, F. Wei, S. Xiang, B. Chen, Z. Zhang, *Angew. Chem. Int. Ed.* **2022**, *61*, e202207579.

[26] F. Yuan, Y. Li, Z. Yuan, L. Li, C. Chen, L. He, H. Lin, X. Fan, B. Chen, S. Xiang, Z. Zhang, *Angew. Chem. Int. Ed.* **2024**, *n/a*, e202414215.

[27] H. Zhang, Y. Li, L. Chen, Y. Yang, H. Lin, S. Xiang, B. Chen, Z. Zhang, *Chem* **2023**, *9*, 242-252.

[28] X.-Q. Wu, T. He, P.-D. Zhang, J. Yu, J.-R. Li, *Chem. Eng. J.* **2024**, *482*, 149115.

[29] Y. Xiao, Y. Chen, W. Wang, X. Bu, P. Feng, *Angew. Chem. Int. Ed.* **2024**, *63*, e202403698.

[30] W. Wang, Y. Chen, P. Feng, X. Bu, *Adv. Mater.* **2024**, *36*, 2403834.

[31] F. Xiang, H. Zhang, Y. Yang, L. Li, Z. Que, L. Chen, Z. Yuan, S. Chen, Z. Yao, J. Fu, S. Xiang, B. Chen, Z. Zhang, *Angew. Chem. Int. Ed.* **2023**, *62*, e202300638.

[32] J. Miao, W. Graham, J. Liu, E. C. Hill, L.-L. Ma, S. Ullah, H.-L. Xia, F.-A. Guo, T. Thonhauser, D. M. Proserpio, J. Li, H. Wang, *J. Am. Chem. Soc.* **2024**, *146*, 84-88.

[33] F. Zheng, R. Chen, Z. Ding, Y. Liu, Z. Zhang, Q. Yang, Y. Yang, Q. Ren, Z. Bao, *J. Am. Chem. Soc.* **2023**, *145*, 19903-19911.

[34] W. Lou, J. Li, W. Sun, Y. Hu, L. Wang, R. F. Neumann, M. Steiner, Z. Gu, B. Luan, Y. Zhang, *Chem. Eng. J.* **2023**, *452*, 139296.

[35] Y.-X. Tan, J. Lin, Q.-H. Li, L. Li, R. Anil Borse, W. Lu, Y. Wang, D. Yuan, *Angew. Chem. Int. Ed.* **2023**, *62*, e202302882.

[36] D. J. O'Hearn, D. Sensharma, A. Raza, A. A. Bezrukov, M. Vandichel, S. Mukherjee, M. J. Zaworotko, *Chem. Sci.* **2024**, *15*, 17937-17943.

[37] L. Li, F. Li, W. Xu, M. Guo, P. Zhu, T. Xing, Z. Li, M. Wang, M. Wu, *Chem. - Eur. J.* **2024**, *n/a*, e202403340.

[38] X. Wang, H. Liu, M. Sun, F. Gao, X. Feng, M. Xu, W. Fan, D. Sun, *Angew. Chem. Int. Ed.* **2024**, *n/a*, e202420801.

[39] Y. Zhang, Y. Han, B. Luan, L. Wang, W. Yang, Y. Jiang, T. Ben, Y. He, B. Chen, *J. Am. Chem. Soc.* **2024**, *146*, 17220-17229.

[40] Y. Zhang, W. Sun, B. Luan, J. Li, D. Luo, Y. Jiang, L. Wang, B. Chen, *Angew. Chem. Int. Ed.* **2023**, *62*, e202309925.

[41] Y. Zhang, L. Yang, L. Wang, S. Duttwyler, H. Xing, *Angew. Chem. Int. Ed.* **2019**, *58*, 8145-8150.

[42] Y. Zhang, L. Yang, L. Wang, X. Cui, H. Xing, *J. Mater. Chem. A* **2019**, *7*, 27560-27566.

[43] Y. Zhang, J. Hu, R. Krishna, L. Wang, L. Yang, X. Cui, S. Duttwyler, H. Xing, *Angew. Chem. Int. Ed.* **2020**, *59*, 17664-17669.

[44] Y. Zhang, L. Wang, J. Hu, S. Duttwyler, X. Cui, H. Xing, *CrystEngComm* **2020**, *22*, 2649-2655.

[45] W. Sun, Y. Jin, Y. Wu, W. Lou, Y. Yuan, S. Duttwyler, L. Wang, Y. Zhang, *Inorganic Chemistry Frontiers* **2022**, *9*, 5140-5147.

[46] L. Wang, W. Sun, Y. Zhang, N. Xu, R. Krishna, J. Hu, Y. Jiang, Y. He, H. Xing, *Angew. Chem. Int. Ed.* **2021**, *60*, 22865-22870.

[47] F. Luo, C. Yan, L. Dang, R. Krishna, W. Zhou, H. Wu, X. Dong, Y. Han, T.-L. Hu, M. O'Keeffe, *J. Am. Chem. Soc.* **2016**, *138*, 5678-5684.

[48] H. Xu, Y. He, Z. Zhang, S. Xiang, J. Cai, Y. Cui, Y. Yang, G. Qian, B. Chen, *J. Mater. Chem. A*

2013, *1*, 77-81.
[49] G. Chang, B. Li, H. Wang, T. Hu, Z. Bao, B. Chen, *Chem. Commun.* **2016**, *52*, 3494-3496.
[50] X. Wang, B. Wang, X. Zhang, Y. Xie, H. Arman, B. Chen, *Inorg. Chem.* **2021**, *60*, 18816-18821.
[51] J.-x. Ma, J. Guo, H. Wang, B. Li, T. Yang, B. Chen, *Inorg. Chem.* **2017**, *56*, 7145-7150.
[52] J. Duan, M. Higuchi, J. Zheng, S.-i. Noro, I. Y. Chang, K. Hyeon-Deuk, S. Mathew, S. Kusaka, E. Sivaniah, R. Matsuda, S. Sakaki, S. Kitagawa, *J. Am. Chem. Soc.* **2017**, *139*, 11576-11583.
[53] J. Duan, W. Jin, R. Krishna, *Inorg. Chem.* **2015**, *54*, 4279-4284.
[54] S. Dutta, S. Mukherjee, O. T. Qazvini, A. K. Gupta, S. Sharma, D. Mahato, R. Babarao, S. K. Ghosh, *Angew. Chem. Int. Ed.* **2022**, *61*, e202114132.
[55] J. Lee, C. Y. Chuah, J. Kim, Y. Kim, N. Ko, Y. Seo, K. Kim, T. H. Bae, E. Lee, *Angew. Chem. Int. Ed.* **2018**, *57*, 7869-7873.
[56] H. Li, C. Liu, C. Chen, Z. Di, D. Yuan, J. Pang, W. Wei, M. Wu, M. Hong, *Angew. Chem. Int. Ed.* **2021**, *60*, 7547-7552.
[57] N. Kumar, S. Mukherjee, N. C. Harvey-Reid, A. A. Bezrukov, K. Tan, V. Martins, M. Vandichel, T. Pham, L. M. van Wyk, K. Oyekan, A. Kumar, K. A. Forrest, K. M. Patil, L. J. Barbour, B. Space, Y. Huang, P. E. Kruger, M. J. Zaworotko, *Chem* **2021**, *7*, 3085-3098.
[58] M. Jiang, X. Cui, L. Yang, Q. Yang, Z. Zhang, Y. Yang, H. Xing, *Chem. Eng. J.* **2018**, *352*, 803-810.
[59] S. Mukherjee, N. Kumar, A. A. Bezrukov, K. Tan, T. Pham, K. A. Forrest, K. A. Oyekan, O. T. Qazvini, D. G. Madden, B. Space, M. J. Zaworotko, *Angew. Chem. Int. Ed.* **2021**, *60*, 10902-10909.
[60] W. Fan, X. Wang, X. Liu, B. Xu, X. Zhang, W. Wang, X. Wang, Y. Wang, F. Dai, D. Yuan, D. Sun, *ACS Sustain. Chem. Eng.* **2019**, *7*, 2134-2140.
[61] O. T. Qazvini, L. K. Macreadie, S. G. Telfer, *Chem. Mater.* **2020**, *32*, 6744-6752.
[62] O. T. Qazvini, R. Babarao, S. G. Telfer, *Chem. Mater.* **2019**, *31*, 4919-4926.
[63] S. Mukherjee, Y. He, D. Franz, S.-Q. Wang, W.-R. Xian, A. A. Bezrukov, B. Space, Z. Xu, J. He, M. J. Zaworotko, *Chem. - Eur. J.* **2020**, *26*, 4923-4929.
[64] J. Pei, H.-M. Wen, X.-W. Gu, Q.-L. Qian, Y. Yang, Y. Cui, B. Li, B. Chen, G. Qian, *Angew. Chem. Int. Ed.* **2021**, *60*, 25068-25074.
[65] X. Zhang, R.-B. Lin, H. Wu, Y. Huang, Y. Ye, J. Duan, W. Zhou, J.-R. Li, B. Chen, *Chem. Eng. J.* **2022**, *431*, 134184.
[66] Y. Ye, S. Xian, H. Cui, K. Tan, L. Gong, B. Liang, T. Pham, H. Pandey, R. Krishna, P. C. Lan, K. A. Forrest, B. Space, T. Thonhauser, J. Li, S. Ma, *J. Am. Chem. Soc.* **2022**, *144*, 1681-1689.
[67] W. Gong, H. Cui, Y. Xie, Y. Li, X. Tang, Y. Liu, Y. Cui, B. Chen, *J. Am. Chem. Soc.* **2021**, *143*, 14869-14876.
[68] S.-C. Fan, Y.-T. Li, Y. Wang, J.-W. Wang, Y.-Y. Xue, H.-P. Li, S.-N. Li, Q.-G. Zhai, *Inorg. Chem.* **2021**, *60*, 18473-18482.
[69] Y.-P. Li, Y. Wang, Y.-Y. Xue, H.-P. Li, Q.-G. Zhai, S.-N. Li, Y.-C. Jiang, M.-C. Hu, X. Bu, *Angew. Chem. Int. Ed.* **2019**, *58*, 13590-13595.
[70] Y.-T. Li, J.-W. Zhang, H.-J. Lv, M.-C. Hu, S.-N. Li, Y.-C. Jiang, Q.-G. Zhai, *Inorg. Chem.* **2020**, *59*, 10368-10373.
[71] J.-W. Zhang, M.-C. Hu, S.-N. Li, Y.-C. Jiang, P. Qu, Q.-G. Zhai, *Chem. Commun.* **2018**, *54*, 2012-2015.
[72] H.-J. Lv, Y.-P. Li, Y.-Y. Xue, Y.-C. Jiang, S.-N. Li, M.-C. Hu, Q.-G. Zhai, *Inorg. Chem.* **2020**,

59, 4825-4834.

[73] X. Duan, Q. Zhang, J. Cai, Y. Yang, Y. Cui, Y. He, C. Wu, R. Krishna, B. Chen, G. Qian, *J. Mater. Chem. A* **2014**, *2*, 2628-2633.

[74] J. Pei, K. Shao, J.-X. Wang, H.-M. Wen, Y. Yang, Y. Cui, R. Krishna, B. Li, G. Qian, *Adv. Mater.* **2020**, *32*, 1908275.

[75] L. Zhang, C. Zou, M. Zhao, K. Jiang, R. Lin, Y. He, C.-D. Wu, Y. Cui, B. Chen, G. Qian, *Cryst. Growth Des.* **2016**, *16*, 7194-7197.

[76] H.-M. Wen, C. Liao, L. Li, L. Yang, J. Wang, L. Huang, B. Li, B. Chen, J. Hu, *Chem. Commun.* **2019**, *55*, 11354-11357.

[77] J. E. Reynolds, K. M. Walsh, B. Li, P. Kunal, B. Chen, S. M. Humphrey, *Chem. Commun.* **2018**, *54*, 9937-9940.

[78] X.-P. Fu, Y.-L. Wang, X.-F. Zhang, R. Krishna, C.-T. He, Q.-Y. Liu, B. Chen, *Chem. Eng. J.* **2022**, *432*, 134433.

[79] Q. Li, N. Wu, J. Li, D. Wu, *Inorg. Chem.* **2020**, *59*, 13005-13008.

[80] J. Zhao, Q. Li, X.-c. Zhu, J. Li, D. Wu, *Inorg. Chem.* **2020**, *59*, 14424-14431.

[81] L. Liu, Z. Yao, Y. Ye, Y. Yang, Q. Lin, Z. Zhang, M. O'Keeffe, S. Xiang, *J. Am. Chem. Soc.* **2020**, *142*, 9258-9266.

[82] Y. Ye, S. Chen, L. Chen, J. Huang, Z. Ma, Z. Li, Z. Yao, J. Zhang, Z. Zhang, S. Xiang, *ACS Appl. Mater. Interfaces* **2018**, *10*, 30912-30918.

[83] Y. Ye, Z. Ma, R.-B. Lin, R. Krishna, W. Zhou, Q. Lin, Z. Zhang, S. Xiang, B. Chen, *J. Am. Chem. Soc.* **2019**, *141*, 4130-4136.

[84] L. Zhang, K. Jiang, Y. Li, D. Zhao, Y. Yang, Y. Cui, B. Chen, G. Qian, *Cryst. Growth Des.* **2017**, *17*, 2319-2322.

[85] L. Zhang, K. Jiang, L. Yang, L. Li, E. Hu, L. Yang, K. Shao, H. Xing, Y. Cui, Y. Yang, B. Li, B. Chen, G. Qian, *Angew. Chem. Int. Ed.* **2021**, *60*, 15995-16002.

[86] L. Meng, L. Yang, C. Chen, X. Dong, S. Ren, G. Li, Y. Li, Y. Han, Z. Shi, S. Feng, *ACS Appl. Mater. Interfaces* **2020**, *12*, 5999-6006.

[87] J. Gao, X. Qian, R.-B. Lin, R. Krishna, H. Wu, W. Zhou, B. Chen, *Angew. Chem. Int. Ed.* **2020**, *59*, 4396-4400.

[88] Y. L. Peng, T. Pham, P. Li, T. Wang, Y. Chen, K. J. Chen, K. A. Forrest, B. Space, P. Cheng, M. J. Zaworotko, *Angew. Chem. Int. Ed.* **2018**, *57*, 10971-10975.

[89] S. Sharma, S. Mukherjee, A. V. Desai, M. Vandichel, G. K. Dam, A. Jadhav, G. Kociok-Köhn, M. J. Zaworotko, S. K. Ghosh, *Chem. Mater.* **2021**, *33*, 5800-5808.

[90] H. S. Scott, M. Shivanna, A. Bajpai, D. G. Madden, K.-J. Chen, T. Pham, K. A. Forrest, A. Hogan, B. Space, J. J. Perry Iv, M. J. Zaworotko, *ACS Appl. Mater. Interfaces* **2017**, *9*, 33395-33400.

[91] H. Yang, T. X. Trieu, X. Zhao, Y. Wang, Y. Wang, P. Feng, X. Bu, *Angew. Chem. Int. Ed.* **2019**, *58*, 11757-11762.

[92] L. Zhang, K. Jiang, J. Zhang, J. Pei, K. Shao, Y. Cui, Y. Yang, B. Li, B. Chen, G. Qian, *ACS Sustain. Chem. Eng.* **2019**, *7*, 1667-1672.

[93] S. Liu, Y. Huang, Q. Dong, H. Wang, J. Duan, *Inorg. Chem.* **2020**, *59*, 9569-9578.

[94] Q. Dong, Y. Guo, H. Cao, S. Wang, R. Matsuda, J. Duan, *ACS Appl. Mater. Interfaces* **2020**, *12*, 3764-3772.

[95] S. Chen, N. Behera, C. Yang, Q. Dong, B. Zheng, Y. Li, Q. Tang, Z. Wang, Y. Wang, J. Duan,

Nano Res. **2021**, *14*, 546-553.
[96] G.-D. Wang, H.-H. Wang, W.-J. Shi, L. Hou, Y.-Y. Wang, Z. Zhu, *J. Mater. Chem. A* **2021**, *9*, 24495-24502.
[97] S.-C. Xiang, Z. Zhang, C.-G. Zhao, K. Hong, X. Zhao, D.-R. Ding, M.-H. Xie, C.-D. Wu, M. C. Das, R. Gill, K. M. Thomas, B. Chen, *Nat. Commun.* **2011**, *2*, 204.
[98] H. Zeng, M. Xie, Y.-L. Huang, Y. Zhao, X.-J. Xie, J.-P. Bai, M.-Y. Wan, R. Krishna, W. Lu, D. Li, *Angew. Chem. Int. Ed.* **2019**, *58*, 8515-8519.
[99] H. Zeng, X.-J. Xie, Y. Wang, D. Luo, R.-J. Wei, W. Lu, D. Li, *Chem. Sci.* **2022**, *13*, 12876-12882.
[100] W. Fan, S. Yuan, W. Wang, L. Feng, X. Liu, X. Zhang, X. Wang, Z. Kang, F. Dai, D. Yuan, D. Sun, H.-C. Zhou, *J. Am. Chem. Soc.* **2020**, *142*, 8728-8737.
[101] T. Li, P. Cui, D. Sun, *Inorg. Chem.* **2022**, *61*, 4251-4256.
[102] K. A. Forrest, T. Pham, K.-J. Chen, X. Jiang, D. G. Madden, D. M. Franz, A. Hogan, M. J. Zaworotko, B. Space, *Langmuir* **2021**, *37*, 13838-13845.
[103] G.-D. Wang, Y.-Z. Li, W.-F. Zhang, L. Hou, Y.-Y. Wang, Z. Zhu, *ACS Appl. Mater. Interfaces* **2021**, *13*, 58862-58870.
[104] H. Cui, Y. Ye, H. Arman, Z. Li, A. Alsalme, R.-B. Lin, B. Chen, *Cryst. Growth Des.* **2019**, *19*, 5829-5835.
[105] H. Li, Z. Ji, C. Chen, Z. Di, Y. Liu, M. Wu, *Cryst. Growth Des.* **2021**, *21*, 2277-2282.
[106] Z. Niu, X. Cui, T. Pham, G. Verma, P. C. Lan, C. Shan, H. Xing, K. A. Forrest, S. Suepaul, B. Space, A. Nafady, A. M. Al-Enizi, S. Ma, *Angew. Chem. Int. Ed.* **2021**, *60*, 5283-5288.
[107] L. Liu, Z. Yao, Y. Ye, L. Chen, Q. Lin, Y. Yang, Z. Zhang, S. Xiang, *Inorg. Chem.* **2018**, *57*, 12961-12968.
[108] T. Xu, Z. Jiang, P. Liu, H. Chen, X. Lan, D. Chen, L. Li, Y. He, *ACS Applied Nano Materials* **2020**, *3*, 2911-2919.
[109] Y. Du, Y. Chen, Y. Wang, C. He, J. Yang, L. Li, J. Li, *Sep. Purif. Technol.* **2021**, *256*, 117749.
[110] N. Xu, Y. Jiang, W. Sun, J. Li, L. Wang, Y. Jin, Y. Zhang, D. Wang, S. Duttwyler, *Molecules* **2021**, *26*, 5121.
[111] J.-P. Zhang, X.-M. Chen, *J. Am. Chem. Soc.* **2009**, *131*, 5516-5521.
[112] N. Kumar, S. Mukherjee, A. A. Bezrukov, M. Vandichel, M. Shivanna, D. Sensharma, A. Bajpai, V. Gascón, K.-i. Otake, S. Kitagawa, M. J. Zaworotko, *SmartMat* **2020**, *1*, e1008.
[113] B. Li, X. Cui, D. O'Nolan, H.-M. Wen, M. Jiang, R. Krishna, H. Wu, R.-B. Lin, Y.-S. Chen, D. Yuan, H. Xing, W. Zhou, Q. Ren, G. Qian, M. J. Zaworotko, B. Chen, *Adv. Mater.* **2017**, *29*, 1704210.
[114] J. Wang, Y. Zhang, P. Zhang, J. Hu, R.-B. Lin, Q. Deng, Z. Zeng, H. Xing, S. Deng, B. Chen, *J. Am. Chem. Soc.* **2020**, *142*, 9744-9751.
[115] J. Li, J.-X. Wu, T. Liu, J. Yang, M.-L. Wei, C. Yang, Q. Dong, Z. Yin, M. Kurmoo, M.-H. Zeng, *Angew. Chem. Int. Ed.* **2024**, *n/a*, e202411150.
[116] Z. Ji, Q. Li, Y. Zhou, R. Krishna, M. Hong, M. Wu, *Angew. Chem. Int. Ed.* **2024**, *63*, e202411175.
[117] H.-M. Wen, C. Yu, M. Liu, C. Lin, B. Zhao, H. Wu, W. Zhou, B. Chen, J. Hu, *Angew. Chem. Int. Ed.* **2023**, *62*, e202309108.
[118] L.-Z. Cai, X.-Y. Yu, M.-S. Wang, D.-Q. Yuan, W.-F. Chen, M.-Y. Wu, G.-C. Guo, *Angew. Chem. Int. Ed.* **2024**, *n/a*, e202417072.

[119] X.-W. Gu, E. Wu, J.-X. Wang, H.-M. Wen, B. Chen, B. Li, G. Qian, *Sci. Adv.* **2023**, *9*, eadh0135.
[120] Z. Zhang, S. B. Peh, Y. Wang, C. Kang, W. Fan, D. Zhao, *Angew. Chem. Int. Ed.* **2020**, *59*, 18927-18932.
[121] X. Cui, K. Chen, H. Xing, Q. Yang, R. Krishna, Z. Bao, H. Wu, W. Zhou, X. Dong, Y. Han, B. Li, Q. Ren, M. J. Zaworotko, B. Chen, *Science* **2016**, *353*, 141-144.
[122] L. Wang, H. Huang, X. Zhang, H. Zhao, F. Li, Y. Gu, *Coord. Chem. Rev.* **2023**, *484*, 215111.
[123] T.-L. Hu, H. Wang, B. Li, R. Krishna, H. Wu, W. Zhou, Y. Zhao, Y. Han, X. Wang, W. Zhu, Z. Yao, S. Xiang, B. Chen, *Nat. Commun.* **2015**, *6*, 7328.
[124] J. Shen, X. He, T. Ke, R. Krishna, J. M. van Baten, R. Chen, Z. Bao, H. Xing, M. Dincă, Z. Zhang, Q. Yang, Q. Ren, *Nat. Commun.* **2020**, *11*, 6259.
[125] S. Yang, A. J. Ramirez-Cuesta, R. Newby, V. Garcia-Sakai, P. Manuel, S. K. Callear, S. I. Campbell, C. C. Tang, M. Schröder, *Nat. Chem.* **2015**, *7*, 121-129.
[126] D. O'Nolan, A. Kumar, K.-J. Chen, S. Mukherjee, D. G. Madden, M. J. Zaworotko, *ACS Applied Nano Materials* **2018**, *1*, 6000-6004.
[127] Q.-L. Qian, X.-W. Gu, J. Pei, H.-M. Wen, H. Wu, W. Zhou, B. Li, G. Qian, *J. Mater. Chem. A* **2021**, *9*, 9248-9255.
[128] H. Li, L. Li, R.-B. Lin, G. Ramirez, W. Zhou, R. Krishna, Z. Zhang, S. Xiang, B. Chen, *ACS Sustain. Chem. Eng.* **2019**, *7*, 4897-4902.
[129] Y.-Z. Li, R. Krishna, F. Xu, W.-F. Zhang, Y. Sui, L. Hou, Y.-Y. Wang, Z. Zhu, *Sep. Purif. Technol.* **2023**, *306*, 122678.
[130] P. Ajayan, W. Wang, Y. Chen, X. Bu, P. Feng, *Adv. Mater.* **2024**, *36*, 2408042.
[131] Y. Liu, J. Liu, H. Xiong, J. Chen, S. Chen, Z. Zeng, S. Deng, J. Wang, *Nat. Commun.* **2022**, *13*, 5515.
[132] W. Zeng, X. Han, X. Li, Y. Situ, Y. Ying, J. Yang, Q. Yang, *Sep. Purif. Technol.* **2025**, *358*, 130308.
[133] H. Xiong, Y. Peng, X. Liu, P. Wang, P. Zhang, L. Yang, J. Liu, H. Shuai, L. Wang, Z. Deng, S. Chen, J. Chen, Z. Zhou, S. Deng, J. Wang, *Adv. Mater.* **2024**, *36*, 2401693.
[134] J. Tian, Q. Chen, F. Jiang, D. Yuan, M. Hong, *Angew. Chem. Int. Ed.* **2023**, *62*, e202215253.
[135] L. Zhang, T. Xiao, X. Zeng, J. You, Z. He, C.-X. Chen, Q. Wang, A. Nafady, A. M. Al-Enizi, S. Ma, *J. Am. Chem. Soc.* **2024**, *146*, 7341-7351.
[136] Y.-Z. Li, G.-D. Wang, F. Xu, Q. Yin, D. Zhao, J. Qi, Y. Sui, L. Hou, Y.-Y. Wang, *Nano Res.* **2023**, 1-8.
[137] D. Li, M.-Y. Gao, C.-H. Deng, G.-B. Li, S.-J. Qin, Q.-Y. Yang, B.-Q. Song, *Small* **2024**, *20*, 2402523.
[138] Q. Zhang, L. Zhou, P. Liu, L. Li, S.-Q. Yang, Z.-F. Li, T.-L. Hu, *Sep. Purif. Technol.* **2022**, *296*, 121404.
[139] H.-Y. Li, Z.-Z. Xue, S.-D. Han, G.-M. Wang, T. He, *Sep. Purif. Technol.* **2025**, *357*, 130094.
[140] J. You, H. Wang, T. Xiao, X. Wu, L. Zhang, C.-Z. Lu, *Chem. Eng. J.* **2023**, *477*, 147001.
[141] L. Wang, Y. Zhang, P. Zhang, X. Liu, H. Xiong, R. Krishna, J. Liu, H. Shuai, P. Wang, Z. Zhou, J. Chen, S. Chen, S. Deng, J. Wang, *AIChE J.* **2024**, *70*, e18396.
[142] J.-W. Wang, S.-C. Fan, H.-P. Li, X. Bu, Y.-Y. Xue, Q.-G. Zhai, *Angew. Chem. Int. Ed.* **2023**, *62*, e202217839.
[143] Y. Chen, Q. Xiong, Y. Wang, Y. Du, Y. Wang, J. Yang, L. Li, J. Li, *Chem. Eng. Sci.* **2021**, *237*,

116572.

[144] C. Jiang, C. Hao, X. Wang, H. Liu, X. Wei, H. Xu, Z. Wang, Y. Ouyang, W. Guo, F. Dai, D. Sun, *Chem. Eng. J.* **2023**, *453*, 139713.

[145] Y. Han, Y. Jiang, J. Hu, L. Wang, Y. Zhang, *Sep. Purif. Technol.* **2024**, *332*, 125777.

[146] W. Fan, S. B. Peh, Z. Zhang, H. Yuan, Z. Yang, Y. Wang, K. Chai, D. Sun, D. Zhao, *Angew. Chem. Int. Ed.* **2021**, *60*, 17338-17343.
